# Single Cell Analysis of Gastric Cancer Reveals Non-Defined Telomere Maintenance Mechanism

**DOI:** 10.3390/cells11213342

**Published:** 2022-10-23

**Authors:** Ji-Yong Sung, Jae-Ho Cheong

**Affiliations:** 1 Department of Laboratory Medicine, Yonsei University College of Medicine, Seoul 03722, Korea; 2 Department of Surgery, Yonsei University College of Medicine, Seoul 03722, Korea; 3 Yonsei Biomedical Research Institute, Yonsei University College of Medicine, Seoul 03722, Korea; 4 Department of Biochemistry & Molecular Biology, Yonsei University College of Medicine, Seoul 03722, Korea

**Keywords:** gastric cancer, non-defined telomere maintenance mechanism, NR4A1, parkin-dependent mitophagy

## Abstract

Telomere maintenance mechanisms (TMMs) are important for cell survival and homeostasis. However, most related cancer research studies have used heterogenous bulk tumor tissue, which consists of various single cells, and the cell type properties cannot be precisely recognized. In particular, cells exhibiting non-defined TMM (NDTMM) indicate a poorer prognosis than those exhibiting alternative lengthening of telomere (ALT)-like mechanisms. In this study, we used bioinformatics to classify TMMs by cell type in gastric cancer (GC) in single cells and compared the biological processes of each TMM. We elucidated the pharmacological vulnerabilities of NDTMM type cells, which are associated with poor prognosis, based on molecular mechanisms. We analyzed differentially expressed genes in cells exhibiting different TMMs in two single-cell GC cohorts and the pathways enriched in single cells. NDTMM type cells showed high stemness, epithelial–mesenchymal transition, cancer hallmark activity, and metabolic reprogramming with mitochondrial abnormalities. Nuclear receptor subfamily 4 group A member 1 (*NR4A1*) activated parkin-dependent mitophagy in association with tumor necrosis factor-alpha (TNFA) to maintain cellular homeostasis without TMM. *NR4A1* overexpression affected TNFA-induced GC cell apoptosis by inhibiting Jun N-terminal kinase/parkin-dependent mitophagy. Our findings also revealed that NR4A1 is involved in cell cycle mediation, inflammation, and apoptosis to maintain cell homeostasis, and is a novel potential therapeutic target in recalcitrant GC.

## 1. Background

Cell immortality, one of the main characteristics of cancer cells, is mediated through telomere maintenance mechanisms (TMMs) [1], which are activated dependently or independently of the telomerase (TEL) enzyme [2]. In approximately 85% of human tumors, TEL ensures telomere maintenance, whereas the ALT mechanism only occurs in 10–15% [3].

The mechanism underlying the maintenance of telomere has been widely investigated in pan-cancer studies [4]. A previous study using bulk RNA-sequencing (RNA-seq) analysis of pan-cancer data of the Cancer Genome Atlas (TCGA) to classify TMMs into four types was the first to identify non-defined TMM (NDTMM) [5]. NDTMM is associated with a poor prognosis in glioblastoma [6]. Although TMM is essential for the immortalization of cancer cells, NDTMM has be shown to frequently occur [7] where a TMM was not observed in the actual bulk tumor tissue. In particular, alternative lengthening of telomere (ALT) is associated with poor prognosis of gastric cancer (GC) and a stem-like molecular mechanism [8]. However, most previous studies used bulk RNA-seq, which limits their relevance and applicability because the specific cell types were not considered [5]. Each TMM differs in its immortalization process and progression into aggressive drug-resistant cancer cells. Although ALT-positive (ALT+) cells have been reported in fibroblasts, few studies have investigated cell types and TMM in other immune or stromal cells. Previous studies investigating the maintenance of telomere in ALT cells analyzed the proliferative potential and telomere dynamics of GM847 ALT cells (SV40 immortalized human skin fibroblasts) co-cultured with normal fibroblasts or TEL+ immortalized human cells [7]. The results revealed that ALT phenotypic repressors were present in normal and some TEL+ immortal cells [9]. Another study identified APBs in TERC+ keratinocytes and squamous cell carcinomas of mice, demonstrating that ALT and TEL coexist in the same cell (SCC) [10]. In the current study, we used a bioinformatics approach to comprehensively classify TMM by cell type at the single-cell level, and comparatively analyzed the biological processes of each TMM type. 

## 2. Methods

### 2.1. Data Preprocessing and Pathway Enrichment Analysis

Two GC single-cell cohorts were used in this study [11]. Data for eight original tumor samples from single cells collected from six patients with stomach cancer were downloaded from the Ji research group website (https://dna-discovery.stanford.edu/research/datasets/ (accessed on 1 January 2022) [11]. Seurat software v. 4.0.3 was used for the single-cell analysis [12]. Quality filters were applied to 12,422 cells from the eight samples. We screened cells that met the following criteria for downstream analysis: cells with a (1) unique feature count of ≥200 and (2) mitochondrial count >5%. 

We identified eight different cell types. Single-cell libraries were created using the Chromium Single-Cell 3 Library & Gel Bead kit v2 (10× Genomics) according to the manufacturer’s instructions, and then sequenced using Illumina sequencers with a single-cell RNA-seq (scRNAseq) technique (i.e., NovaSeq, HiSeq, and NextSeq). We examined 84 metabolic pathways from the Kyoto Encyclopedia of Genes and Genomes (KEGG) database to determine their activity. Markov affinity-based graph imputation of cells (MAGIC) was used to impute missing values and restore the structure of the single-cell data [13]. We used the single-sample gene set enrichment plugin in the R package gene set variation analysis (GSVA) [14] to acquire information regarding the pathway activity of each cell. 

We calculated the GSVA score to analyze the TMM pathway enrichment, and attempted 10,000,000 runs to judge the accuracy and significance (false discovery rate [FDR] < 0.01). The TMM-related gene signature was obtained from a previous study [15]. The second single-cell cohort (5927 cells) was downloaded from the Gene Express Omnibus (GEO) database (GSE150290) from tumor samples from 23 patients [16]. The method used to select the marker gene for cell type classification was described in a previous study [11,16]. 

MSigDB (http://software.broadinstitute.org/gsea/msigdb, accessed on 2 February 2022) includes epithelial–mesenchymal transition (EMT) signatures as well as 50 cancer hallmark gene sets [17]. We also used microarray data generated from bulk samples of 497 stomach cancer patients from the Yonsei cohort (registration number: GSE84437, accessed 2 February 2022) from the GEO database [18]. TCGA-stomach adenocarcinoma (STAD) was used for Gene Expression Profiling Interactive Analysis 2 (GEPIA2) [19] to validate patient survival.

### 2.2. Analysis of DEGs and Single-Cell Meta-Analysis

DESingle was used to examine the differentially expressed genes (DEGs) in cells based on their TMM type [20]. Pathways enriched with METASCAPE [21] for significantly affected genes selected using DEG, protein–protein interaction (PPI), molecular complex detection (MCODE), transcription factors (TF), and other software were analyzed. The R packages RaceID and StemID were used to identify single-cell stemness [22]. Signatures related to stemness were obtained from previous studies [23] and cell types were categorized from microarray Y497 bulk samples [18] using the CIBERSORT software [24]. 

### 2.3. Drug Target Analysis

In order to investigate gene regulatory interactions with genes overexpressed in the NDTMM type as input, we analyzed the DEGs [20] to generate a list of genes with high or low expression in each TMM type and ConsensusPathDB (CPDB; http://cpdb.molgen.mpg.de/ (accessed on 29 March 2022)) [25]. The target gene and drug were discovered through a search of the Cancer Cell Line Encyclopedia (CCLE) [26].

## 3. Results 

### 3.1. Single-Cell Analysis Revealed TMM Type in GC

We analyzed single-cell data from two GC cohorts (12,422 and 5927 cells). By comparing cell type categorization and TMM type in single-cell data subjected to imputation, we determined the TMM types of single cells. TMM types were classified into four categories in order to determine the most prevalent type in each cell, and six pathways were assessed using the gene profile from our previous pan-cancer TMM study [5].

**Figure 1:**
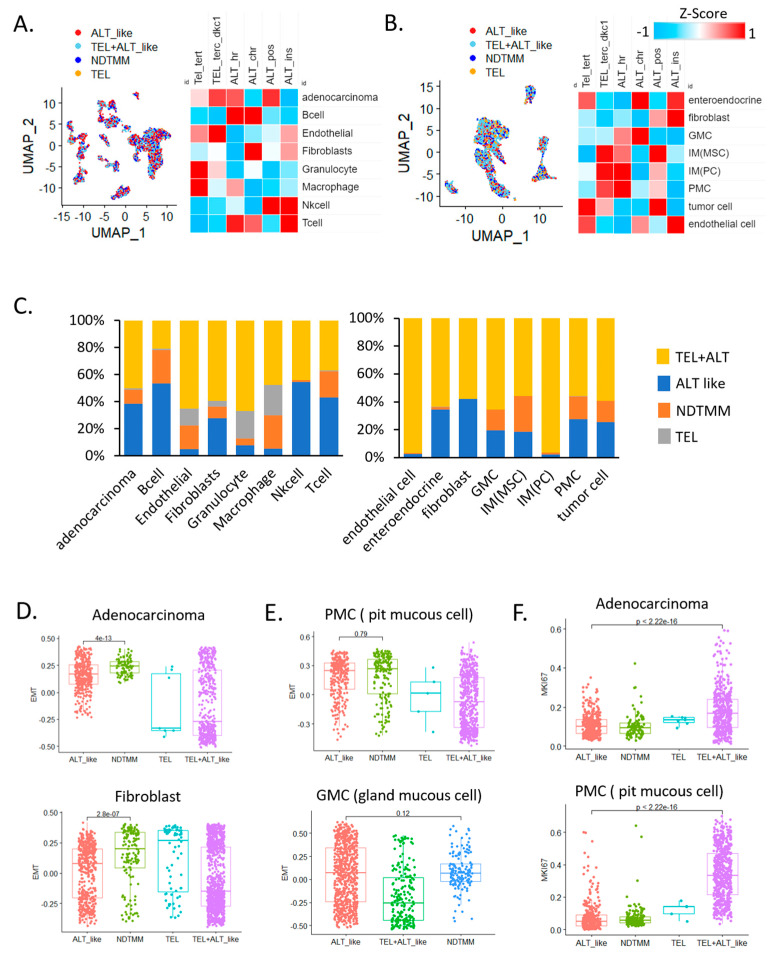
Single−cell analysis revealed telomere maintenance mechanisms (TMMs) in gastric cancer (GC). Uniform manifold approximation and projection (UMAP) and heat map of TMM activity of eight single−cell types in (**A**) adenocarcinoma, B cells, endothelial cells, fibroblasts, granulocytes, macrophages, natural killer (NK) cells, and T cells (12,422 cells, cohort 1) and (**B**) enteroendocrine cells, fibroblasts, gland mucous cells (GMC), mesenchymal stem cells, pit mucous cell (PMC), tumor cells, and endothelial cells (5927 cells, cohort 2). (**C**) Bar graph showing frequency of TMM types in eight single−cell types (12,422 cells, left); different cohort eight single−cell types (5927 cells, right). Boxplot of epithelial–mesenchymal transition (EMT) signature of (**D**) adenocarcinoma cells, fibroblasts, (**E**) PMC, and GMC. (**F**) Boxplot of MKI67 expression in adenocarcinoma cells (cohort 1) and PMCs.

In adenocarcinoma cells, telomerase (TEL) and ALT activity were observed simultaneously, and ALT activity was high in B, T, and NK cells, which are related to innate immunity. Telomerase activity was relatively higher in endothelial cells and granulocytes than it was in other cells (Figure 1A). This trend was similar to that observed in the second GC single-cell cohort (Figure 1B), and telomerase activity was higher in endothelial cells than it was in other cells (FDR < 0.01). In gland mucous cells (GMC) and fibroblasts, ALT activity was relatively high. According to analysis of the frequency of TMM types in different cells, 39% and 10% of adenocarcinoma cells showed ALT-like and 10% NDTMM, respectively, whereas very few cells exhibited telomerase activity (Figure 1C). 

This was similar to a study that reported approximately 30–40% and 30% ALT activity in a GC cohort and in tumor cells of another cohort, respectively [5]. Specifically, in endothelial cells, granulocytes, and macrophages, the number of cells exhibiting ALT and telomerase activities was low and relatively high, respectively. These results suggest that the TMM of each cell type was different and the preferred type was used for maintenance. In addition, the various cell types also included those with a previously unknown TMM (NDTMM). 

We also analyzed the relationship of the four TMM and specific cell types to EMT. NDTMM showed the highest EMT activity in adenocarcinoma cells. In fibroblasts from the same cohort, telomerase exhibited the highest EMT activity. In another cohort, pit mucous cells (PMC) showed a similar tendency to that of adenocarcinoma cells, and although it was not significantly different from the ALT-like type, EMT was highest in the NDTMM type. In GMCs, ALT-like and NDTMM types exhibited similar trends (Figure 1D,E). We considered TMM to be closely related to cell growth, maintenance, differentiation, and proliferation, and a clear difference existed in MKI67 expression. 

The number of ALT-like and NDTMM type cells was relatively low in adenocarcinoma; however, telomerase (TEL) and TEL+ALT-like type cells showed high proliferation. A similar trend was observed in PMC cells, and TEL+ALT-like type cells predominately showed high proliferation (Figure 1F). Next, we analyzed the correlation between immune cells and TMM in a 497 Yonsei Hospital cohort (Y497) from the bulk transcriptome data, and the correlation was clearly separated (positively/negatively). Our analysis revealed that ALT chromatin-like type-related cells were T regulatory, naïve B, and memory B cells, and the remaining the immune cells were positively correlated with both TEL and ALT pathways (Supplementary Figure S1A). 

We stratified patients by TMM type using bulk transcriptome data. In both the 497 Yonsei [18] and TCGA-STAD cohorts, the four TMM types were not significantly distinguishable; however, the same pattern showed the best prognosis in the TEL only group, and the poorest prognosis was observed in the NDTMM type patients (Supplementary Figure S1B). Therefore, these results indicate that the TMM of each cell type, which was not identified in the bulk sample, was detected at the single-cell level. Thus, TMM heterogeneity was observed by cell type.

### 3.2. Cancer Hallmark Activity and TMM at Single-Cell Level

**Figure 2:**
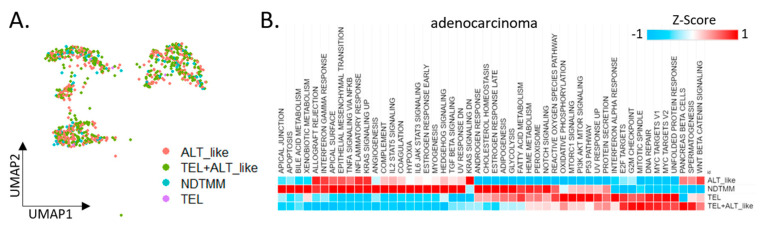

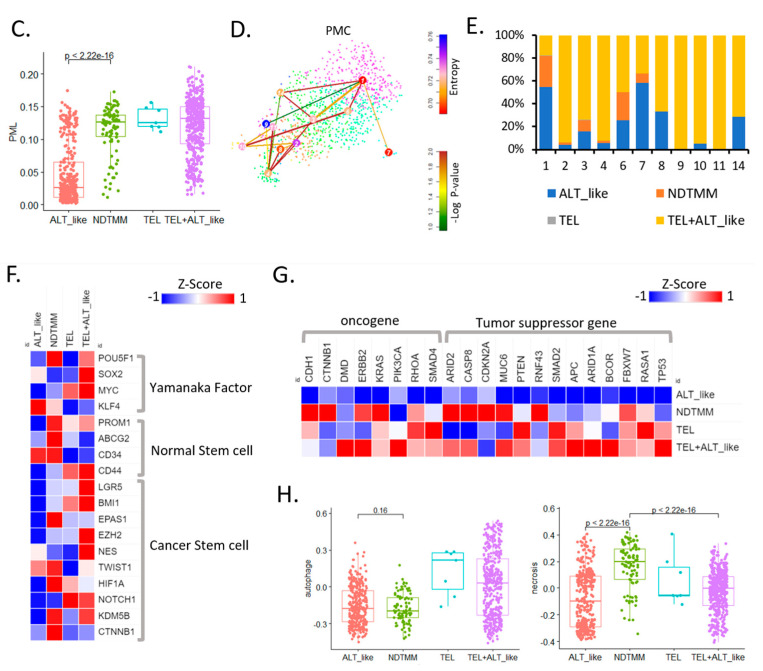
Cancer hallmark and telomere maintenance mechanism (TMM) at the single-cell level. (**A**) UMAP of adenocarcinoma cells with four TMM types (ALT-like, TEL+ALT-like, non-defined telomere maintenance mechanism [NDTMM], TEL). (**B**) Heat map of cancer hallmark of adenocarcinoma cells categorized by four TMM types. (**C**) Boxplot of PML expression in the adenocarcinoma cells categorized by four TMM types. (**D**) tSNE plot of PMC stemness by entropy. (**E**) Bar graph of frequency of TMM types by stemness cluster. (**F**) Heat map of stemness related genes of four TMM types in adenocarcinoma. (**G**) Heat map of oncogene and tumor suppressor gene by four TMM types in adenocarcinoma. (**H**) Boxplot of autophagy and necrosis signature of the four TMM types in adenocarcinoma.

We divided adenocarcinoma cells into four TMM types and analyzed the differences in each cell type with respect to the characteristics observed in the bulk (Figure 2A). Initially, 924 adenocarcinoma cells from the first cohort were classified into four TMM types, and 50 cancer hallmark pathways were analyzed. Contrary to our expectations, >60% of the cancer hallmark activity was confirmed in the NDTMM type cells. In the ALT-like type cells, both the KRAS and Wnt-catenin signaling pathways were highly enriched (Figure 2B). 

We predicted the telomere length based on the expression of the promyelocytic leukemia (*PML*) gene, and determined that the higher the *PML* expression, the shorter the telomere length and the higher the ability to maintain telomeres. In our adenocarcinoma data, TEL+ALT-like was the most common TMM type and, interestingly, the prevalence of the NDTMM type was higher than that of the ALT-like type. In the ALT-like type cells, the telomeres were predicted to be long, whereas they were assumed to be shortened in the NDTMM type cells (Figure 1C). 

We analyzed the stemness of cells in the PMC using StemID [22] through entropy calculation using the stemness analysis. Cluster 9 showed the highest entropy, whereas clusters 1 and 7 showed low entropy (FDR < 0.05, Figure 2D). The classification of the four TMM types for each cluster according to the degree of entropy revealed numerous TEL+ALT-like type cells in the high-entropy cluster, and ALT-like and NDTMM type cells were relatively common in clusters 1 and 7. In cluster 1, ALT-like and NDTMM type cells accounted for 50% and 20%, respectively (Figure 2E). 

We performed a stemness marker analysis of adenocarcinomas in order to determine the stemness marker genes associated with TMM. The Yamanaka factor was mainly high in the TEL+ALT-like type, whereas the expression level of the normal stem cell marker genes (*PROM1*, *ABCG2*, and *CD34*) was high in the NDTMM type. Furthermore, the expression of the cancer stem cell marker gene was high in the NDTMM and TEL+ALT-like types, with similar ratios (Figure 2F). Next, we analyzed the expression of the oncogenes and tumor suppressor genes. In the case of ALT-like type cells, most genes exhibited low activity. In the NDTMM, TEL, and TEL+ALT-like type cells, the opposite trend was observed (Figure 2G). 

TMM is related to cell survival, and autophagy signature analysis confirmed that the TEL type was high but the NDTMM type was low, similarly to findings with the ALT-like type (Figure 2H). However, with the necrosis signature, the NDTMM and ATL-like types showed the highest and lowest activity, respectively (Figure 2H). These results confirmed that the NDTMM type cells had short telomeres just before death, possessed high EMT and stemness, and were highly related to cancer hallmarks. Conversely, the ALT-like type cells possessed elongated telomeres, and exhibited low necrosis and entropy.

### 3.3. Differentially Expressed Biological Pathways for TMM Type

**Figure 3:**
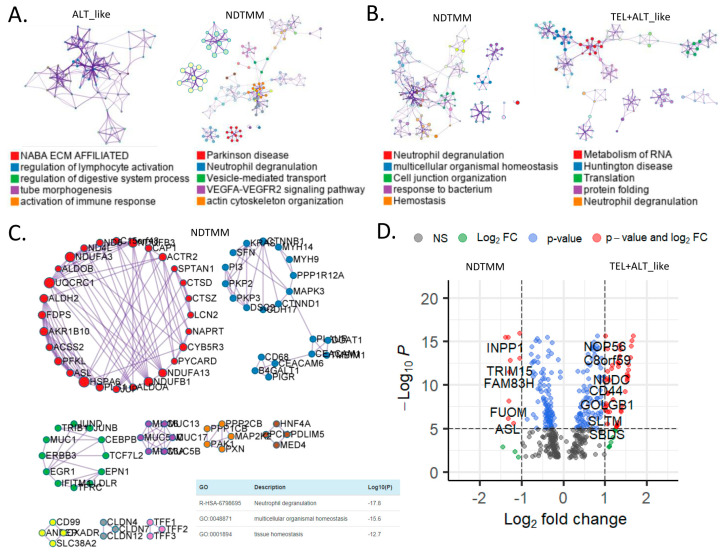

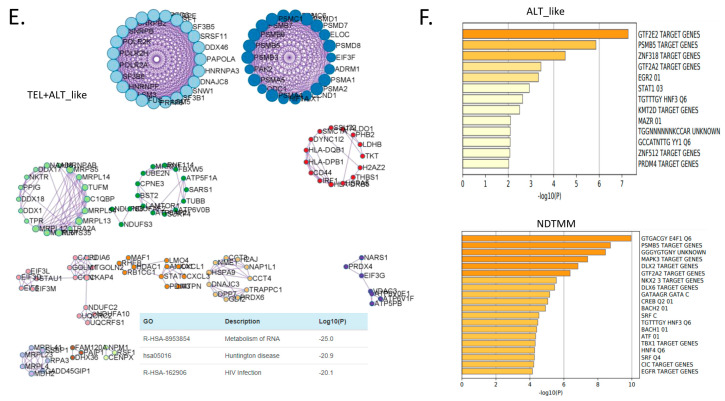
Biological pathways related to differentially expressed genes (DEGs) of different telomere maintenance mechanism (TMM) types in adenocarcinoma. Enriched biological network for (**A**) ALT-like (left) and NDTMM (right) and (**B**) NDTMM (left) and TEL+ALT-like (right) types of adenocarcinoma. (**C**) PPI network of nine clusters for NDTMM type analyzed using MCODE algorithm. (**D**) Volcano plot of DEGs between NDTMM and TEL+ALT-like types of adenocarcinoma. (**E**) PPI network of 12 clusters of TEL+ALT-like type cells analyzed using MCODE. (**F**) Bar graph of TF for ALT-like and NDTMM types.

Currently, information about other biological pathways for each TMM type in GC is limited, and we previously reported ALT-like type TMM and epigenetic characteristics on the basis of bulk RNA-seq analysis in GC [8]. In this study, differently enriched pathways for each TMM type at the adenocarcinoma single-cell level were analyzed. The ALT-like type was enriched for NABA ECM-affiliated pathways and regulation of lymphocyte activation, and the NDTMM type was enriched for Parkinson’s disease, neutrophil degranulation, and vascular endothelial growth factor-alpha (VEGFA) signaling pathways (Figure 3A). Interestingly, for NDTMM, pathways such as multicellular organismal and cellular maintenance homeostasis were enriched. 

In contrast, for the TEL+ALT-like type, pathways such as RNA metabolism, Huntington’s disease, and translation were enriched (Figure 3B). We analyzed the PPI of genes that were differently expressed between the NDTMM and ALT-like type using MCODE, and the genes were classified into nine MCODE clusters. Genes were mainly enriched in neutrophil degranulation, generation of precursor metabolites and energy, and ATP metabolism (Figure 3C). It has been recognized that these genes create an environment for NDTMM type cells to generate a large amount of energy for survival. The maintenance of the gastrointestinal epithelium and epithelial structure of adenocarcinoma cells to maintain survival in a TMM-deficient environment is also of interest. 

Gene ontology (GO) term analysis of the nine MCODE clusters revealed that neutrophil degradation, multicellular organismal homeostasis, and tissue homeostasis were enriched. *REG3A*, *H1F0*, and *KRT7* were highly expressed in the ALT-like type cells (Supplementary Table S1). We compared genes that were significantly different between NDTMM and TEL+ALT groups using DEG analysis. In the NDTMM type, the expression of *INPP1*, *TRIM15*, *FAM83H*, *FUOM*, and *ASL* was high, and in the TEL+ALT-like type, the expression of *NOP56*, *C8orf59*, *NUDC*, and *CD44* was high (Figure 3D). 

Based on these results, we conducted an MCODE analysis of the PPI of genes that were highly significantly expressed in the TEL+ALT-like type, which were classified into 12 clusters. These clusters were enriched for RNA metabolism, Huntington’s disease, and human immunodeficiency virus (HIV) infection (Figure 3E). We determined that the TFs in ALT-like and NDTMM types, general transcription factor IIE subunit 2 (GTF2E2), and PSMB5, were ALT-like, whereas proteasome 20S subunit beta 5 (PSMB5) was also found in NDTMM (Figure 3F). Specifically, early growth response 2 (EGR2) [27] was identified as a TEL inhibitory TF.

### 3.4. Landscape of Metabolic Reprogramming of TMM Types

In ALT+ cells, telomere length is increased and PGC-1, a key regulator of mitochondrial biogenesis and function, is amplified or overexpressed in ALT+ tumors, which are very sensitive to *PGC-1* or *SOD2* knockdown. In order to improve anti-TEL cancer therapy, genetic modeling of TEL elimination exposes vulnerabilities that accidentally suppress mitochondrial homeostasis and oxidative defense systems [28]. However, currently, few studies have reported metabolic reprogramming based on other TMM types. We systematically analyzed the metabolic reprogramming of adenocarcinoma and other cell types at the single-cell level using 84 metabolic pathways from KEGG [29]. 

In the ALT-like type of adenocarcinoma cells, linoleic acid metabolism, nitrogen, glycosphingolipid biosynthesis, and glycosaminoglycan-related pathway activities were high. Tricyclic acid (TCA) and oxidative phosphorylation (OXPOHS) showed high activity in the TEL type cells; whereas in the NDTMM type cells, high activity was observed in >50% of the pathways. The metabolic pathway activity was not as high as expected in the TEL+ALT-like group, which also showed higher activity in riboflavin metabolism, glycosaminoglycan biosynthesis, and heparan sulfate than in other processes. 

**Figure 4:**
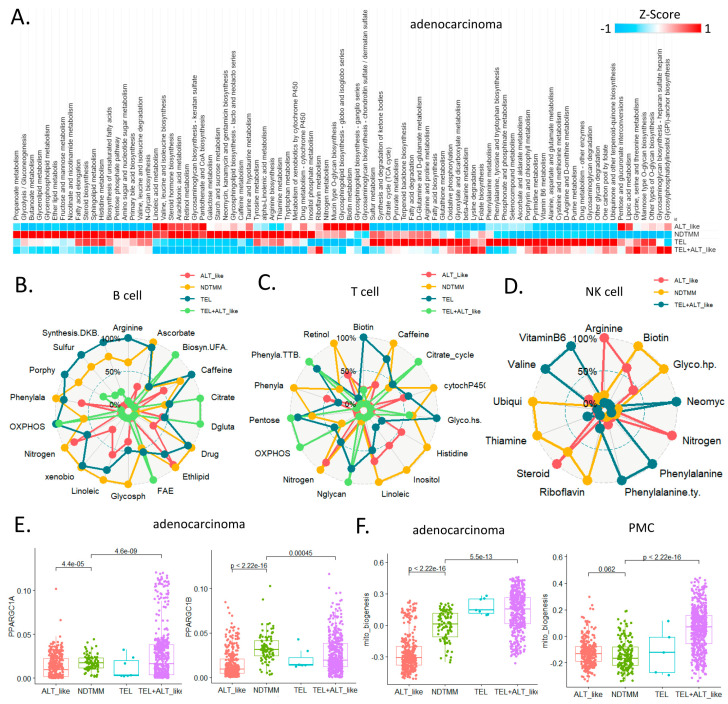

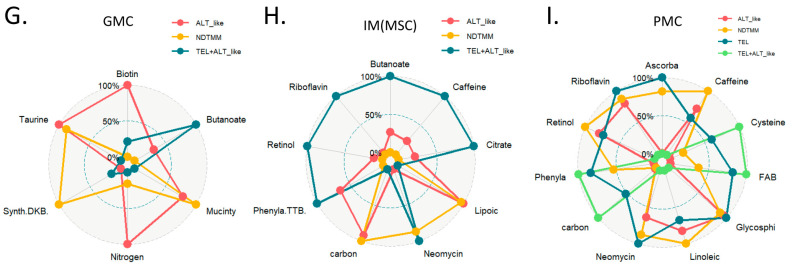
Landscape of metabolic reprogramming of and telomere maintenance mechanism (TMM) types. (**A**) Heatmap of metabolic reprogramming landscape of cells with four TMM types in adenocarcinoma. Spider plots of top enriched metabolic pathways for (**B**) B, (**C**) T, and (**D**) NK cells. (**E**) Box plot of *PPARGC1A*, *PPARGC1B* (mitochondria bioenergetics master regulators) for adenocarcinoma cells. (**F**) Boxplot of mitochondria bioenergetics signature score for adenocarcinoma cells and PMC. Spider plots of top enriched metabolic pathways for (**G**) GMC, (**H**) intestinal metaplasia (IM), metaplasia stem cell (MSC), and (**I**) PMC.

The innate immune cells also demonstrated a pattern of metabolic reprogramming that was clearly distinguished according to TMM type (Figure 4A). NDTMM and TEL type B cells showed high metabolic reprogramming and used various energy sources, whereas the ALT-like type cells showed high ether lipid metabolism activity (Figure 4B). NDTMM type T cells showed high nitrogen, linoleic acid, histidine, and phenylalanine metabolism, and the TEL+ALT-like type demonstrated high OXPHOS and TCA activity (Figure 4C). NK cells showed three types of TMM. 

In the ALT-like type cells, nitrogen metabolism, steroid metabolism, and arginine activity were high, and in the NDTMM type, glycosaminoglycan biosynthesis and heparan sulfate, thiamine, and biotin metabolism were high (Figure 4D). The master regulators of mitochondrial bioenergetics, *PGC1A* and *PGC1B*, showed the highest activity in NDTMM type adenocarcinoma cells (Figure 4E). However, in the mitochondrial bioenergetics signature analysis, the TEL+ALT-like type and TEL type showed high activity, whereas the ALT type exhibited the lowest activity. This trend was also observed in the PMC of other GC single-cell cohorts, showing the highest and lowest activity in the TEL+ALT-like and NDTMM types, respectively (Figure 4F). We analyzed the metabolic reprogramming of GMC, intestinal metaplasia (IM, MSC), and PMC in single-cell cohorts of different GCs. 

The ALT-like type GMC showed high nitrogen metabolism activity, similar to that of T and NK cells. Taurine and hypotaurine metabolism, as well as mucin type o-glycan biosynthesis, were high in both the ALT-like and NDTMM type cells (Figure 4G). In the IM (MSE), TCA was high in the TEL+ALT-like group, and the ALT-like and NDTMM groups showed similar trends (Figure 4H). In PMC, NDTMM and ALT-like types showed similar trends, and TEL+ALT-like type showed high activity in one-carbon metabolism, fatty acid biosynthesis, phenylalanine metabolism, and cysteine and methionine metabolism, unlike other types (Figure 4I). 

These results demonstrate that the energy metabolism pathways changed according to TMM type at the single-cell level, and differed within the same cell type. Cells select TMM to maintain cellular homeostasis and, thus, different metabolic reprogramming methods are used as energy sources for survival. In summary, these results may provide insights into cell type-specific TMM inhibition and the development of metabolic inhibitory drugs.

### 3.5. Vulnerabilities of NDTMM Type for Cancer Therapy

**Figure 5:**
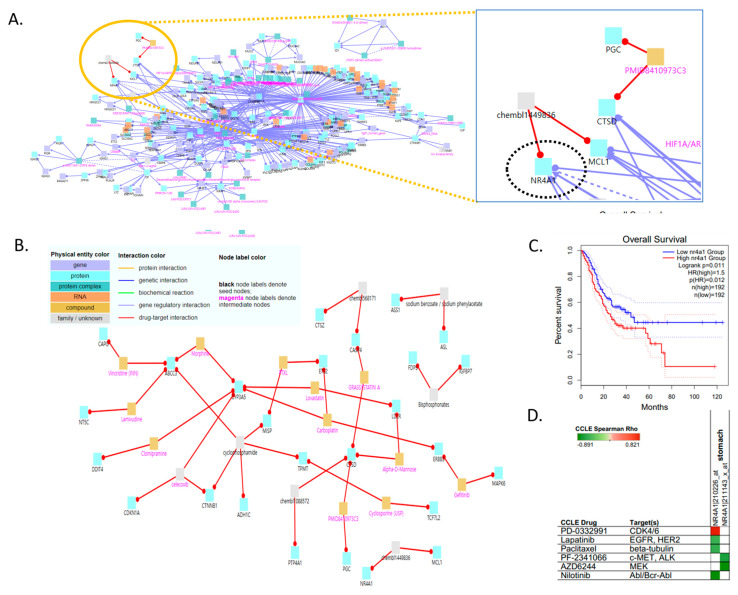
Vulnerabilities of non-defined telomere maintenance mechanism NDTMM type for cancer therapy. (**A**) Gene regulatory interaction network for NDTMM type of adenocarcinoma constructed using ConsensusPathDB. (CPDB) (**B**) Drug–target interaction for NDTMM type of adenocarcinoma. (**C**) Kaplan–Meier plots show overall survival rates for high and low *NR4A1* expression (*p* < 0.011). (**D**) Drug and target genes for gastric cell line molecular subtypes from the Cancer Cell Line Encyclopedia (CCLE). High drug sensitivity is indicated in green (negative correlation).

Our categorization of adenocarcinoma cells into four TMM types and examination of their features confirmed that NDTMM and ALT-like types were vital to the development of aggressive GC cells. We analyzed gene regulatory and drug–target interactions using the induced network modules of CPDB to identify a drug target for the NDTMM type [25] (Figure 5A).

When the intermediate node z-score threshold was set to 100, the drug targets and drugs at the upper level were as follows: chembl1449836, nuclear receptor subfamily 4 group A member 1 (NR4A1), MCL1/sodium benzoate, sodium phenylacetate, ASL, ASS1/GRASSYSTATIN A, PMID8410973C3, chembl1088572, CTSD, CASP4, PGC, and PTP4A1 (Figure 5B). We investigated NR4A1 as the drug target because it plays an important role in the development of mitochondrial abnormalities in NDTMM type cells, as well as in cell maintenance in the absence of TMM. We also confirmed the association of NR4A1 with survival in the TCGA-STAD dataset (Figure 5C). We also identified prospective therapeutics for *NR4A1* (nilotinib, AZD6244, PF-2341066, and paclitaxel; in stomach cancer cell line, Figure 5D).

Tumor necrosis factor (TNF) causes mitochondrial dysfunction in GC, as demonstrated by Cyt-c leakage, mitochondrial membrane potential (MMP) collapse, and energy metabolism disturbance, which all activate cellular death processes. TNF therapy resulted in GC cell death, but also induced protective mitophagy. TNF boosted the activity of c-JNK, which raised parkin expression, subsequently triggering mitophagy to remove damaged mitochondria and prevent cell death. Mitophagy was reduced by overexpression of NR4A1, making GC cells more susceptible to TNF-induced cell death [30].

## 4. Discussion

Telomere maintenance is essential for cancer cell survival and proliferation [31]. However, our study demonstrates that cancer cells maintain their own survival and proliferation by changing and using the cancer microenvironment [32] to their advantage, even where TMM is absent. We defined four types of TMM, but the current knowledge about the NDTMM type in GC is sparse. Although the ALT-like mechanism was associated with a poorer survival prognosis in GC, the NDTMM type was associated with a more deleterious condition than the ALT-like type was. Overall, the NDTMM type displayed strong cancer hallmark activity, as well as the highest EMT and stemness among the four TMM types. 

This feature of the NDTMM type led us to infer the length of telomeres based on PML expression. NDTMM type cells had telomeres that were shorter than those of the ALT-like type cells, but longer than those of the TEL+ALT-like type cells. NDTMM type cells were previously thought to be cancer cells immediately before death due to autophagy or high necrosis that could not maintain their telomeres. However, contrary to our hypotheses, the pathways that were enriched in this cell type were mostly metabolic and cellular homeostasis and maintenance pathways. Despite a lack of TMM, cancer cells can continuously attempt to avoid death. 

ALT-like cells were previously considered to possess a strong mitochondrial bioenergetic activity. However, our findings revealed that NDTMM type cells which were more active than ALT-like cells were present in more than half of the metabolic pathways. This tendency was observed in adenocarcinoma and other innate immune cells, and cell-specific metabolic reprogramming of the NDTMM type was exploited as an energy source. *NR4A1* was determined to be a potential therapeutic target based on our observation of the regulatory interactions of the differential genes that were substantially expressed in the NDTMM type cells. In TMM-free cancer cells, *NR4A1* prevents cell death via a JNK/parkin-dependent mitophagy. Overexpression of *NR4A1* causes a mitochondrial energy imbalance by suppressing the expression of the mitochondrial respiratory complex. 

Our findings also demonstrated that TNF therapy stimulated parkin-dependent mitophagy, which, in excess, inhibits mitochondrial apoptosis, reducing the cytotoxicity of TNF. In contrast, *NR4A1* overexpression reduced parkin-dependent mitophagy by inhibiting JNK. In addition, our study demonstrates the potential of *NR4A1* as a novel drug target for killing NDTMM type cancer cells by inhibiting JNK/parkin-dependent mitophagy, making GC cells more susceptible to TNF-induced apoptosis. To the best of our knowledge, this is the first study to show that the NDTMM type is more detrimental in GC than the ALT-like type. Our findings will contribute to the development of precision medicine strategies for patients with refractory GC and provide unique insights into the development of anticancer therapies.

## 5. Conclusions

This study demonstrates the survival, maintenance, and proliferation of GC cells in absence of a TMM. Cancer cells were classified into four types on based on TMM types, and the NDTMM type has been sparingly studied. We comparatively analyzed TMM activity, DEGs, PPI, and enriched GO terms among individual TMM type cells in GC, and demonstrated that the NDTMM type cells maintained their survival, proliferation, and homeostasis by altering their cell environments. Furthermore, we identified the *NR4A1* gene as a potential therapeutic target for the development of efficient anticancer therapies.

## Data Availability

All data are available in the main text or the supplementary materials.

## Supplementary Materials

The following supporting information can be downloaded at: https://www.mdpi.com/article/10.3390/cells11213342/s1, Figure S1: Correlation of immune cells and TMM pathway in Y497 cohort; Table S1: List of differential expressed genes (DEGs) between ALT-like type and NDTMM type.

## References

[1] Rhyu M.S. (1995). Telomeres, telomerase, and immortality. J. Natl. Cancer Inst..

[2] Colgin M.L., Reddel R.R. (1999). Telomere maintenance mechanisms and cellular immortalization. Curr. Opin. Genet. Dev..

[3] Barthel F.P., Wei W., Tang M., Martinez-Ledesma E., Hu X., Amin S.B., Akdemir K.C., Seth S., Song X., Wang Q. (2017). Systematic analysis of telomere length and somatic alterations in 31 cancer types. Nat. Genet..

[4] Sung J.-Y., Lim H.-W., Joung J.-G., Park W.-Y. (2020). Pan-Cancer Analysis of Alternative Lengthening of Telomere Activity. Cancers.

[5] Sung Y.J., Cheong J.H. (2021). Pan-Cancer Analysis of Clinical Relevance via Telomere Maintenance Mechanism. Int. J. Mol. Sci..

[6] Royds J.A., Al Nadaf S., Wiles A.K., Chen Y.-J., Ahn A., Shaw A., Bowie S., Lam F., Baguley B.C., Braithwaite A.W. (2011). The CDKN2A G500 allele is more frequent in GBM patients with no defined telomere maintenance mechanism tumors and is associated with poorer survival. PLoS ONE.

[7] Claude E., Decottignies A. (2020). Telomere maintenance mechanisms in cancer: Telomerase, ALT or lack thereof. Curr. Opin. Genet. Dev..

[8] Sung Y.J., Cheong J.H. (2021). Alternative lengthening of telomeres is mechanistically linked to potential therapeutic vulnerability in the stem-like subtype of gastric cancer. Clin. Transl. Med..

[9] Perrem K., Bryan T.M., Englezou A., Hackl T., Moy E.L., Reddel R.R. (1999). Repression of an alternative mechanism for lengthening of telomeres in somatic cell hybrids. Oncogene.

[10] Perrem K., Booth R.E., Jin Y., Zhou X., Crowe D.L. (2015). Alternative lengthening of telomeres in cancer stem cells in vivo. Oncogene.

[11] Sathe A., Grimes S.M., Lau B.T., Chen J., Suarez C., Huang R.J., A Poultsides G., Ji H.P. (2020). Single-Cell Genomic Characterization Reveals the Cellular Reprogramming of the Gastric Tumor Microenvironment. Clin. Cancer Res..

[12] Hao Y., Hao S., Andersen-Nissen E., Mauck W.M., Zheng S., Butler A., Lee M.J., Wilk A.J., Darby C., Zager M. (2021). Integrated analysis of multimodal single-cell data. Cell.

[13] van Dijk D., Sharma R., Nainys J., Yim K., Kathail P., Carr A.J., Burdziak C., Moon K.R., Chaffer C.L., Pattabiraman D. (2018). Recovering Gene Interactions from Single-Cell Data Using Data Diffusion. Cell.

[14] Hanzelmann S., Castelo R., Guinney J. (2013). GSVA: Gene set variation analysis for microarray and RNA-seq data. BMC Bioinform..

[15] Nersisyan L., Hopp L., Loeffler-Wirth H., Galle J., Loeffler M., Arakelyan A., Binder H. (2019). Telomere Length Maintenance and Its Transcriptional Regulation in Lynch Syndrome and Sporadic Colorectal Carcinoma. Front. Oncol..

[16] Kim J., Park C., Kim K.H., Kim E.H., Kim H., Woo J.K., Seong J.K., Nam K.T., Lee Y.C., Cho S.Y. (2022). Single-cell analysis of gastric pre-cancerous and cancer lesions reveals cell lineage diversity and intratumoral heterogeneity. NPJ Precis. Oncol..

[17] Hanahan D., Weinberg R.A. (2011). Hallmarks of cancer: The next generation. Cell.

[18] Cheong J.-H., Yang H.-K., Kim H., Kim W.H., Kim Y.-W., Kook M.-C., Park Y.-K., Kim H.-H., Lee H.S., Lee K.H. (2018). Predictive test for chemotherapy response in resectable gastric cancer: A multi-cohort, retrospective analysis. Lancet Oncol..

[19] Tang Z., Li C., Kang B., Gao G., Li C., Zhang Z. (2017). GEPIA: A web server for cancer and normal gene expression profiling and interactive analyses. Nucleic Acids Res..

[20] Miao Z., Deng K., Wang X., Zhang X. (2018). DEsingle for detecting three types of differential expression in single-cell RNA-seq data. Bioinformatics.

[21] Zhou Y., Zhou B., Pache L., Chang M., Khodabakhshi A.H., Tanaseichuk O., Benner C., Chanda S.K. (2019). Metascape provides a biologist-oriented resource for the analysis of systems-level datasets. Nat. Commun..

[22] Grün D., Muraro M.J., Boisset J.-C., Wiebrands K., Lyubimova A., Dharmadhikari G., Born M.V.D., van Es J., Jansen E., Clevers H. (2016). De Novo Prediction of Stem Cell Identity using Single-Cell Transcriptome Data. Cell Stem Cell.

[23] Malta T.M., Sokolov A., Gentles A.J., Burzykowski T., Poisson L., Weinstein J.N., Kamińska B., Huelsken J., Omberg L., Gevaert O. (2018). Machine Learning Identifies Stemness Features Associated with Oncogenic Dedifferentiation. Cell.

[24] Newman A.M., Steen C.B., Liu C.L., Gentles A.J., Chaudhuri A.A., Scherer F., Khodadoust M.S., Esfahani M.S., Luca B.A., Steiner D. (2019). Determining cell type abundance and expression from bulk tissues with digital cytometry. Nat. Biotechnol..

[25] Herwig R., Hardt C., Lienhard M., Kamburov A. (2016). Analyzing and interpreting genome data at the network level with ConsensusPathDB. Nat. Protoc..

[26] Qin Y., Conley A.P., Grimm E.A., Roszik J. (2017). A tool for discovering drug sensitivity and gene expression associations in cancer cells. PLoS ONE.

[27] Tyler E.J., del Arroyo A.G., Hughes B.K., Wallis R., Garbe J.C., Stampfer M.R., Koh J., Lowe R., Philpott M.P., Bishop C.L. (2021). Early growth response 2 (EGR2) is a novel regulator of the senescence programme. Aging Cell.

[28] Hu J., Hwang S.S., Liesa M., Gan B., Sahin E., Jaskelioff M., Ding Z., Ying H., Boutin A.T., Zhang H. (2012). Antitelomerase therapy provokes ALT and mitochondrial adaptive mechanisms in cancer. Cell.

[29] Xiao Z., Dai Z., Locasale J.W. (2019). Metabolic landscape of the tumor microenvironment at single cell resolution. Nat. Commun..

[30] Yan H., Xiao F., Zou J., Qiu C., Sun W., Gu M., Zhang L. (2018). NR4A1-induced increase in the sensitivity of a human gastric cancer line to TNFalpha-mediated apoptosis is associated with the inhibition of JNK/Parkin-dependent mitophagy. Int. J. Oncol..

[31] Bearss J.D., Hurley L.H., von Hoff D.D. (2000). Telomere maintenance mechanisms as a target for drug development. Oncogene.

[32] Sung J.Y., Cheong J.H. (2022). New Immunometabolic Strategy Based on Cell Type-Specific Metabolic Reprogramming in the Tumor Immune Microenvironment. Cells.

