# Supplementary material for: Single Cell Analysis of Gastric Cancer Reveals Non-Defined Telomere Maintenance Mechanism
Source: Cells. 2022 Oct 23;11(21):3342. doi: 10.3390/cells11213342 (PMC9657924; doi:10.3390/cells11213342)
Supplement: Supplementary file 1 [file cells-11-03342-s001.zip › Supplementary Table S1.pdf]

ID	theta_1	theta_2	mu_1	mu_2	size_1	size_2	prob_1	prob_2	total_meai	total_meai	foldChang	log2fc	norm_tota	norm_tota	norm_fold	chi2LR1	pvalue_LR:	pvalue_LR.	FDR_LR2	FDR_LR3	pvalue	pvalue.adj	Remark	Type	State
SLC22A18	4.07E-09	2.04E-10	0.132022	0.71134	5.51E+11	5.78E+09	1	1	0.132022	0.71134	0.185597	-0.73143	0.132022	0.71134	0.185597	78.71971	2.16E-05	8.17E-07	4.35E-05	1.65E-06	1.11E-16	6.22E-15	NA	DEg	down
PET100	4.01E-09	1.49E-10	0.168539	0.793814	3.36E+13	4.31E+09	1	1	0.168539	0.793814	0.212316	-0.67302	0.168539	0.793814	0.212316	78.47355	5.29E-13	0	2.30E-12	0	1.11E-16	6.22E-15	NA	DEg	down
TMEM256	4.01E-09	1.49E-10	0.168539	0.793814	3.36E+13	4.31E+09	1	1	0.168539	0.793814	0.212316	-0.67302	0.168539	0.793814	0.212316	78.47355	5.29E-13	0	2.30E-12	0	1.11E-16	6.22E-15	NA	DEg	down
HLA-F	4.79E-09	1.53E-10	0.160112	0.773196	3.92E+12	4.16E+09	1	1	0.157303	0.773196	0.203446	-0.69155	0.160112	0.773196	0.207079	78.10766	2.55E-10	0	7.76E-10	0	1.11E-16	6.22E-15	NA	DEg	down
RBM47	4.74E-09	1.73E-10	0.143258	0.731959	1.05E+12	5.77E+09	1	1	0.143258	0.731959	0.195719	-0.70837	0.143258	0.731959	0.195719	77.50174	1.02E-10	3.66E-15	3.19E-10	1.48E-14	1.11E-16	6.22E-15	NA	DEg	down
GHITM	3.50E-09	2.04E-10	0.134831	0.71134	8.49E+11	5.78E+09	1	1	0.134831	0.71134	0.189546	-0.72229	0.134831	0.71134	0.189546	77.40905	0.000207	1.30E-06	0.000366	2.59E-06	1.11E-16	6.22E-15	NA	DEg	down
FDFT1	3.21E-09	2.05E-10	0.176966	0.804124	1.49E+14	2.81E+09	1	1	0.176966	0.804124	0.220073	-0.65743	0.176966	0.804124	0.220073	77.36306	1.23E-12	0	4.87E-12	0	1.11E-16	6.22E-15	NA	DEg	down
IGHG2	3.31E-09	1.53E-10	0.162921	0.773196	6.20E+12	4.16E+09	1	1	0.162921	0.773196	0.210712	-0.67631	0.162921	0.773196	0.210712	76.91638	1.95E-12	0	7.54E-12	0	1.11E-16	6.22E-15	NA	DEg	down
MT-ND6	2.93E-09	1.63E-10	0.168539	0.783505	3.01E+12	4.8E+09	1	1	0.16573	0.783505	0.211524	-0.67464	0.168539	0.783505	0.215109	76.52956	1.26E-12	0	4.94E-12	0	2.22E-16	1.03E-14	NA	DEg	down
SREK1IP1	5.22E-09	1.73E-10	0.146067	0.731959	1.17E+12	5.77E+09	1	1	0.143258	0.731959	0.195719	-0.70837	0.146067	0.731959	0.199557	76.34015	7.56E-11	7.95E-07	2.42E-10	1.62E-06	2.22E-16	1.03E-14	NA	DEg	down
APP	3.61E-09	1.73E-10	0.146067	0.731959	1.92E+12	5.77E+09	1	1	0.146067	0.731959	0.199557	-0.69993	0.146067	0.731959	0.199557	76.32647	3.99E-11	2.59E-07	1.30E-10	5.43E-07	2.22E-16	1.03E-14	NA	DEg	down
TMEM141	3.61E-09	1.73E-10	0.146067	0.731959	1.92E+12	5.77E+09	1	1	0.146067	0.731959	0.199557	-0.69993	0.146067	0.731959	0.199557	76.32647	3.99E-11	2.59E-07	1.30E-10	5.43E-07	2.22E-16	1.03E-14	NA	DEg	down
NENF	1.71E-09	1.25E-10	0.227528	0.907216	3.69E+12	3.42E+09	1	1	0.227528	0.907216	0.250798	-0.60068	0.227528	0.907216	0.250798	76.285	5.55E-16	0	3.45E-15	0	2.22E-16	1.03E-14	NA	DEg	down
SCP2	4.38E-09	1.53E-10	0.16573	0.773196	4.28E+12	4.16E+09	1	1	0.16573	0.773196	0.214345	-0.66889	0.16573	0.773196	0.214345	75.77007	0.000643	0	0.001065	0	2.22E-16	1.03E-14	NA	DEg	down
ARPC1B	3.21E-09	1.49E-10	0.176966	0.793814	1.49E+14	4.31E+09	1	1	0.176966	0.793814	0.222932	-0.65183	0.176966	0.793814	0.222932	75.46993	0.022741	5.42E-09	0.030299	1.37E-08	3.33E-16	1.29E-14	NA	DEg	down
CTNND1	3.50E-09	2.17E-10	0.134831	0.701031	8.49E+11	5.9E+09	1	1	0.134831	0.701031	0.192333	-0.71595	0.134831	0.701031	0.192333	75.38888	2.74E-08	2.68E-08	7.23E-08	6.26E-08	3.33E-16	1.29E-14	NA	DEg	down
TMBIM1	1.29E-08	3.62E-10	0.109551	0.639175	8.7E+09	2.12E+10	1	1	0.106742	0.639175	0.166999	-0.77729	0.109551	0.639175	0.171394	75.18095	0.048087	2.76E-05	0.060193	4.72E-05	3.33E-16	1.29E-14	NA	DEg	down
GOLIM4	5.80E-09	2.45E-10	0.117978	0.659794	9.61E+10	1.08E+10	1	1	0.117978	0.659794	0.17881	-0.74761	0.117978	0.659794	0.17881	75.16644	0.00018	4.24E-08	0.000322	9.53E-08	3.33E-16	1.29E-14	NA	DEg	down
SLC40A1	5.80E-09	2.45E-10	0.117978	0.659794	9.61E+10	1.08E+10	1	1	0.117978	0.659794	0.17881	-0.74761	0.117978	0.659794	0.17881	75.16644	0.00018	4.24E-08	0.000322	9.53E-08	3.33E-16	1.29E-14	NA	DEg	down
MUC5B	3.96E-09	1.49E-10	0.176966	0.793814	3.20E+12	4.31E+09	1	1	0.174157	0.793814	0.219393	-0.65878	0.176966	0.793814	0.222932	75.03193	1.62E-12	0	6.31E-12	0	3.33E-16	1.29E-14	NA	DEg	down
CD9	5.65E-09	1.45E-10	0.342697	1.123711	7.86E+13	2.77E+09	1	1	0.320225	1.123711	0.284971	-0.5452	0.342697	1.123711	0.304969	74.97786	3.33E-15	0	1.94E-14	0	3.33E-16	1.29E-14	NA	DEg	down
CLDN12	4.07E-09	2.25E-10	0.132022	0.690722	5.51E+11	6.89E+09	1	1	0.132022	0.690722	0.191137	-0.71866	0.132022	0.690722	0.191137	74.65653	0.004214	2.38E-07	0.00639	5.01E-07	4.44E-16	1.68E-14	NA	DEg	down
CAPZB	3.61E-09	1.89E-10	0.146067	0.721649	1.92E+12	5.6E+09	1	1	0.146067	0.721649	0.202408	-0.69377	0.146067	0.721649	0.202408	74.26895	9.52E-11	6.24E-09	3.00E-10	1.56E-08	5.55E-16	1.92E-14	NA	DEg	down
ECHS1	4.36E-09	2.17E-10	0.13764	0.701031	7.80E+11	5.9E+09	1	1	0.13764	0.701031	0.19634	-0.70699	0.13764	0.701031	0.19634	74.1188	1.55E-05	2.51E-09	3.18E-05	6.51E-09	5.55E-16	1.92E-14	NA	DEg	down
NBL1	3.98E-09	1.63E-10	0.174157	0.783505	1.04E+14	4.8E+09	1	1	0.174157	0.783505	0.22228	-0.6531	0.174157	0.783505	0.22228	74.08801	1.40E-05	0	2.90E-05	0	5.55E-16	1.92E-14	NA	DEg	down
CDH17	2.26E-09	1.63E-10	0.171348	0.783505	3.38E+14	4.8E+09	1	1	0.171348	0.783505	0.218695	-0.66016	0.171348	0.783505	0.218695	74.00146	9.97E-13	0	4.07E-12	0	5.55E-16	1.92E-14	NA	DEg	down
MUC17	4.13E-09	2.33E-10	0.129213	0.680412	4.27E+11	7.86E+09	1	1	0.129213	0.680412	0.189905	-0.72146	0.129213	0.680412	0.189905	73.93021	0.009792	4.08E-06	0.013989	7.53E-06	6.66E-16	2.12E-14	NA	DEg	down
DAD1	4.38E-09	1.71E-10	0.16573	0.762887	4.28E+12	4.19E+09	1	1	0.16573	0.762887	0.217241	-0.66306	0.16573	0.762887	0.217241	73.85457	0.009963	1.17E-09	0.01421	3.15E-09	6.66E-16	2.12E-14	NA	DEg	down
SERINC2	5.56E-09	1.71E-10	0.16573	0.762887	2.41E+12	4.19E+09	1	1	0.160112	0.762887	0.209877	-0.67804	0.16573	0.762887	0.217241	73.81494	1.07E-11	3.33E-16	3.86E-11	1.57E-15	6.66E-16	2.12E-14	NA	DEg	down
CDH1	5.52E-09	2.94E-10	0.103933	0.618557	2.29E+10	3.23E+10	1	1	0.103933	0.618557	0.168024	-0.77463	0.103933	0.618557	0.168024	73.81337	6.65E-05	6.00E-05	0.000125	0.000101	6.66E-16	2.12E-14	NA	DEg	down
INSIG1	2.95E-09	2.12E-10	0.157303	0.742268	5.57E+12	3.92E+09	1	1	0.157303	0.742268	0.211923	-0.67382	0.157303	0.742268	0.211923	73.46959	1.70E-10	0	5.21E-10	0	7.77E-16	2.43E-14	NA	DEg	down
CAPNS1	3.31E-09	1.92E-10	0.162921	0.752577	6.20E+12	3.96E+09	1	1	0.162921	0.752577	0.216485	-0.66457	0.162921	0.752577	0.216485	73.06641	0.017087	8.28E-13	0.023288	2.74E-12	8.88E-16	2.67E-14	NA	DEg	down
COL17A1	5.68E-09	2.76E-10	0.109551	0.628866	3.81E+10	3.14E+10	1	1	0.109551	0.628866	0.174203	-0.75894	0.109551	0.628866	0.174203	73.06256	0.001614	1.51E-06	0.002563	2.97E-06	8.88E-16	2.67E-14	NA	DEg	down
STK24	2.93E-09	1.71E-10	0.168539	0.762887	3.01E+12	4.19E+09	1	1	0.16573	0.762887	0.217241	-0.66306	0.168539	0.762887	0.220923	72.7159	0.000675	0	0.001115	0	1.11E-15	3.22E-14	NA	DEg	down
VEGFA	1.16E-08	1.71E-10	0.168539	0.762887	5.39E+11	4.19E+09	1	1	0.160112	0.762887	0.209877	-0.67804	0.168539	0.762887	0.220923	72.71261	8.96E-11	3.33E-16	2.84E-10	1.57E-15	1.11E-15	3.22E-14	NA	DEg	down
MAPK3	7.59E-09	2.45E-10	0.123595	0.659794	7.05E+10	1.08E+10	1	1	0.120787	0.659794	0.183067	-0.73739	0.123596	0.659794	0.187324	72.48175	0.001488	2.10E-08	0.002384	4.93E-08	1.22E-15	3.48E-14	NA	DEg	down
RIOK3	5.46E-09	2.94E-10	0.106742	0.618557	3.07E+10	3.23E+10	1	1	0.106742	0.618557	0.172566	-0.76305	0.106742	0.618557	0.172566	72.3988	8.90E-08	1.53E-06	2.24E-07	3.00E-06	1.33E-15	3.55E-14	NA	DEg	down
SLC9A3R1	6.19E-09	3.62E-10	0.115169	0.639175	6.66E+10	2.12E+10	1	1	0.115169	0.639175	0.180183	-0.74429	0.115169	0.639175	0.180183	72.38968	1.96E-06	4.40E-13	4.38E-06	1.51E-12	1.33E-15	3.55E-14	NA	DEg	down
PTTG1IP	3.45E-09	1.53E-10	0.174157	0.773196	2.21E+12	4.16E+09	1	1	0.171348	0.773196	0.22161	-0.65441	0.174157	0.773196	0.225243	72.37579	0.000267	1.98E-11	0.000466	6.00E-11	1.33E-15	3.55E-14	NA	DEg	down
KRTCAP2	2.54E-09	2.12E-10	0.160112	0.742268	8.13E+12	3.92E+09	1	1	0.160112	0.742268	0.215707	-0.66614	0.160112	0.742268	0.215707	72.31308	0.022994	0	0.03059	0	1.33E-15	3.55E-14	NA	DEg	down
VAPA	2.72E-09	2.40E-10	0.199438	0.824742	2.36E+13	2.3E+09	1	1	0.199438	0.824742	0.241819	-0.61651	0.199438	0.824742	0.241819	71.94029	7.11E-10	0	2.08E-09	0	1.67E-15	4.29E-14	NA	DEg	down
IFI6	2.22E-09	0.574062	1.275281	0.992351	7.3E+09	11267.97	1	0.999912	1.014045	0.42268	2.399082	0.380045	1.275281	0.42268	3.017128	71.86209	0	0	0	0	1.67E-15	4.29E-14	NA	DEg	up
DMBT1	4.46E-09	2.50E-10	0.120787	0.649485	1.79E+11	1.29E+10	1	1	0.120787	0.649485	0.185973	-0.73055	0.120787	0.649485	0.185973	71.76278	1.59E-07	1.41E-05	3.93E-07	2.47E-05	1.78E-15	4.51E-14	NA	DEg	down
GPI	2.41E-09	1.63E-10	0.179775	0.783505	9.61E+13	4.8E+09	1	1	0.179775	0.783505	0.22945	-0.63931	0.179775	0.783505	0.22945	71.59547	1.51E-11	0	5.33E-11	0	2.00E-15	4.92E-14	NA	DEg	down
RHOF	3.47E-09	2.04E-10	0.148876	0.71134	2.49E+12	5.78E+09	1	1	0.148876	0.71134	0.20929	-0.67925	0.148876	0.71134	0.20929	71.11671	1.14E-10	5.73E-12	3.53E-10	1.82E-11	2.44E-15	5.75E-14	NA	DEg	down
S100A13	3.47E-09	2.04E-10	0.148876	0.71134	2.49E+12	5.78E+09	1	1	0.148876	0.71134	0.20929	-0.67925	0.148876	0.71134	0.20929	71.11671	1.14E-10	5.73E-12	3.53E-10	1.82E-11	2.44E-15	5.75E-14	NA	DEg	down
PSCA	0.030319	0.763907	0.915394	0.742325	41537.74	12109.44	0.999978	0.999939	0.648876	0.175258	3.702412	0.568485	0.88764	0.175258	5.064772	71.11347	0.000577	6.06E-13	0.00096	2.03E-12	2.44E-15	5.75E-14	NA	DEg	up
DNAJC15	2.68E-09	2.05E-10	0.191011	0.804124	1.56E+14	2.81E+09	1	1	0.188202	0.804124	0.234046	-0.6307	0.191011	0.804124	0.23754	71.05366	1.54E-13	1.10E-10	7.07E-13	3.17E-10	2.55E-15	5.92E-14	NA	DEg	down
MPC2	2.68E-09	2.40E-10	0.202247	0.824742	5.34E+13	2.3E+09	1	1	0.199438	0.824742	0.241819	-0.61651	0.202247	0.824742	0.245225	70.88176	2.74E-11	2.16E-09	9.36E-11	5.65E-09	2.78E-15	6.35E-14	NA	DEg	down
RAB1A	4.79E-09	1.73E-10	0.160112	0.731959	3.92E+12	5.77E+09	1	1	0.157303	0.731959	0.214907	-0.66775	0.160112	0.731959	0.218745	70.55484	3.15E-09	1.11E-16	8.66E-09	5.49E-16	3.22E-15	7.26E-14	NA	DEg	down
TSTD1	4.03E-09	1.53E-10	0.179775	0.773196	6.01E+12	4.16E+09	1	1	0.176966	0.773196	0.228876	-0.6404	0.179775	0.773196	0.232509	70.20672	1.00E-06	0	2.33E-06	0	3.89E-15	8.64E-14	NA	DEg	down
ANXA4	4.36E-09	2.33E-10	0.13764	0.680412	7.80E+11	7.86E+09	1	1	0.13764	0.680412	0.20229	-0.69403	0.13764	0.680412	0.20229	70.14991	3.30E-08	6.66E-15	8.69E-08	2.62E-14	4.00E-15	8.77E-14	NA	DEg	down
ETHE1	1.21E-08	2.31E-10	0.286517	1.010309	5.08E+14	6.57E+08	1	1	0.272472	1.010309	0.269692	-0.56913	0.286517	1.010309	0.283593	70.08245	1.54E-11	0	5.41E-11	0	4.11E-15	8.89E-14	NA	DEg	down
ERRFI1	3.21E-09	1.71E-10	0.176966	0.762887	1.49E+14	4.19E+09	1	1	0.176966	0.762887	0.231969	-0.63457	0.176966	0.762887	0.231969	69.86477	1.22E-07	0	3.02E-07	0	4.55E-15	9.73E-14	NA	DEg	down
JUP	5.49E-09	1.92E-10	0.171348	0.752577	2.97E+12	3.96E+09	1	1	0.16573	0.752577	0.220217	-0.65715	0.171348	0.752577	0.227682	69.76772	0.003474	5.52E-11	0.005313	1.62E-10	4.77E-15	1.01E-13	NA	DEg	down
TRAM1	2.41E-09	1.53E-10	0.179775	0.773196	9.61E+13	4.16E+09	1	1	0.179775	0.773196	0.232509	-0.63356	0.179775	0.773196	0.232509	69.74172	3.07E-11	0	1.03E-10	0	4.88E-15	1.02E-13	NA	DEg	down
SMIM24	1.33E-08	2.77E-10	0.191011	0.793814	4.06E+12	2.24E+09	1	1	0.182584	0.793814	0.230009	-0.63826	0.191011	0.793814	0.240625	69.66914	2.26E-11	1.78E-08	7.84E-11	4.24E-08	5.00E-15	1.03E-13	NA	DEg	down
PTP4A2	3.17E-09	2.05E-10	0.196629	0.804124	2.24E+13	2.81E+09	1	1	0.196629	0.804124	0.244526	-0.61167	0.196629	0.804124	0.244526	69.35886	3.43E-14	0	1.75E-13	0	5.88E-15	1.19E-13	NA	DEg	down
PPP1CB	2.68E-09	2.26E-10	0.202247	0.814433	5.34E+13	2.5E+09	1	1	0.199438	0.814433	0.24488	-0.61105	0.202247	0.814433	0.248329	69.08887	7.48E-13	0	3.14E-12	0	6.66E-15	1.34E-13	NA	DEg	down
BZW1	2.31E-09	1.63E-10	0.188202	0.783505	6.29E+13	4.8E+09	1	1	0.188202	0.783505	0.240206	-0.61942	0.188202	0.783505	0.240206	68.95371	0.000118	0	0.000216	0	7.11E-15	1.39E-13	NA	DEg	down
XBP1	1.46E-09	1.13E-10	0.244382	0.896907	5.50E+12	4.41E+09	1	1	0.241573	0.896907	0.26934	-0.5697	0.244382	0.896907	0.272472	68.88188	3.00E-15	0	1.76E-14	0	7.44E-15	1.44E-13	NA	DEg	down
CAMK2N1	0.403515	2.86E-10	0.654585	1.092784	19268.36	1.65E+09	0.999966	1	0.342697	1.092784	0.3136	-0.50362	0.390449	1.092784	0.357298	68.73653	2.63E-09	0.000327	7.27E-09	0.00052	7.99E-15	1.53E-13	NA	DEg	down
PGK1	1.92E-09	1.17E-10	0.219101	0.845361	7.37E+12	3.24E+09	1	1	0.219101	0.845361	0.259181	-0.5864	0.219101	0.845361	0.259181	68.6616	8.64E-13	0	3.61E-12	0	8.22E-15	1.55E-13	NA	DEg	down
CAPZA2	4.18E-09	3.62E-10	0.123596	0.639175	2.55E+11	2.12E+10	1	1	0.123596	0.639175	0.193367	-0.71362	0.123596	0.639175	0.193367	68.43813	0.003467	3.86E-06	0.005311	7.18E-06	9.21E-15	1.70E-13	NA	DEg	down
GSKIP	4.18E-09	3.62E-10	0.123596	0.639175	2.55E+11	2.12E+10	1	1	0.123596	0.639175	0.193367	-0.71362	0.123596	0.639175	0.193367	68.43813	0.003467	3.86E-06	0.005311	7.18E-06	9.21E-15	1.70E-13	NA	DEg	down
CES2	0.511945	2.67E-10	0.753967	0.979381	16013.08	1.97E+09	0.999953	1	0.325843	0.979381	0.332703	-0.47794	0.367978	0.979381	0.375724	68.36681	2.20E-11	7.45E-12	7.65E-11	2.33E-11	9.55E-15	1.71E-13	NA	DEg	down
IFITM1	2.26E-09	1.92E-10	0.171348	0.752577	3.38E+14	3.96E+09	1	1	0.171348	0.752577	0.227682	-0.64267	0.171348	0.752577	0.227682	68.34303	2.82E-11	0	9.47E-11	0	9.66E-15	1.71E-13	NA	DEg	down
MYDGF	2.26E-09	1.92E-10	0.171348	0.752577	3.38E+14	3.96E+09	1	1	0.171348	0.752577	0.227682	-0.64267	0.171348	0.752577	0.227682	68.34303	2.82E-11	0	9.47E-11	0	9.66E-15	1.71E-13	NA	DEg	down
H2AFY	2.72E-09	2.05E-10	0.199438	0.804124	2.36E+13	2.81E+09	1	1	0.199438	0.804124	0.248019	-0.60551	0.199438	0.804124	0.248019	68.34142	1.60E-13	0	7.25E-13	0	9.66E-15	1.71E-13	NA	DEg	down
SMDT1	2.07E-09	2.26E-10	0.205056	0.814433	2.42E+13	2.5E+09	1	1	0.205056	0.814433	0.251778	-0.59898	0.205056	0.814433	0.251778	68.2679	2.44E-12	0	9.34E-12	0	9.99E-15	1.74E-13	NA	DEg	down
BCL10	6.88E-09	2.85E-10	0.098315	0.57732	9.78E+09	5.01E+10	1	1	0.098315	0.57732	0.170295	-0.7688	0.098315	0.57732	0.170295	68.25289	8.64E-07	4.60E-11	2.01E-06	1.35E-10	1.01E-14	1.75E-13	NA	DEg	down
ALKBH7	4.38E-09	1.73E-10	0.16573	0.731959	4.28E+12	5.77E+09	1	1	0.16573	0.731959	0.22642	-0.64508	0.16573	0.731959	0.22642	68.18005	2.82E-11	1.11E-16	9.47E-11	5.49E-16	1.04E-14	1.78E-13	NA	DEg	down
AGPAT2	2.87E-09	1.53E-10	0.185393	0.773196	5.32E+13	4.16E+09	1	1	0.185393	0.773196	0.239775	-0.6202	0.185393	0.773196	0.239775	68.14398	3.97E-12	0	1.49E-11	0	1.07E-14	1.80E-13	NA	DEg	down
TMEM45B	4.13E-09	2.50E-10	0.129213	0.649485	4.27E+11	1.29E+10	1	1	0.129213	0.649485	0.198948	-0.70126	0.129213	0.649485	0.198948	67.91289	0.019376	8.99E-06	0.02601	1.61E-05	1.20E-14	2.01E-13	NA	DEg	down
NDUFA2	1.95E-09	2.33E-10	0.216292	0.835052	9.61E+12	1.74E+09	1	1	0.216292	0.835052	0.259017	-0.58667	0.216292	0.835052	0.259017	67.80487	1.05E-12	0	4.26E-12	0	1.25E-14	2.08E-13	NA	DEg	down
LAPTM4A	3.30E-09	1.63E-10	0.191011	0.783505	3.00E+13	4.8E+09	1	1	0.191011	0.783505	0.243791	-0.61298	0.191011	0.783505	0.243791	67.75253	6.67E-14	0	3.25E-13	0	1.29E-14	2.11E-13	NA	DEg	down
EIF1AX	2.14E-09	1.17E-10	0.22191	0.845361	4.76E+12	3.24E+09	1	1	0.22191	0.845361	0.262503	-0.58087	0.22191	0.845361	0.262503	67.6705	4.45E-13	0	1.95E-12	0	1.34E-14	2.18E-13	NA	DEg	down
MT-ATP8	3.00E-09	2.05E-10	0.202247	0.804124	1.67E+13	2.81E+09	1	1	0.202247	0.804124	0.251513	-0.59944	0.202247	0.804124	0.251513	67.4595	1.16E-12	1.77E-13	4.65E-12	6.14E-13	1.49E-14	2.36E-13	NA	DEg	down
ARHGDIA	3.50E-09	2.45E-10	0.134831	0.659794	8.49E+11	1.08E+10	1	1	0.134831	0.659794	0.204354	-0.68962	0.134831	0.659794	0.204354	67.43269	4.06E-08	1.71E-07	1.05E-07	3.64E-07	1.51E-14	2.36E-13	NA	DEg	down
CDA	3.50E-09	2.45E-10	0.134831	0.659794	8.49E+11	1.08E+10	1	1	0.134831	0.659794	0.204354	-0.68962	0.134831	0.659794	0.204354	67.43269	4.06E-08	1.71E-07	1.05E-07	3.64E-07	1.51E-14	2.36E-13	NA	DEg	down
HADHA	3.50E-09	2.45E-10	0.134831	0.659794	8.49E+11	1.08E+10	1	1	0.134831	0.659794	0.204354	-0.68962	0.134831	0.659794	0.204354	67.43269	4.06E-08	1.71E-07	1.05E-07	3.64E-07	1.51E-14	2.36E-13	NA	DEg	down
ATP5F1C	1.69E-09	1.36E-10	0.233146	0.865979	8.99E+10	2.27E+09	1	1	0.233146	0.865979	0.269228	-0.56988	0.233146	0.865979	0.269228	67.41254	3.72E-14	3.33E-16	1.88E-13	1.57E-15	1.53E-14	2.37E-13	NA	DEg	down
TRIB1	1.86E-09	1.59E-10	0.238764	0.876289	7.03E+12	1.94E+09	1	1	0.235955	0.876289	0.269266	-0.56982	0.238764	0.876289	0.272472	67.32002	1.55E-15	0	9.41E-15	0	1.60E-14	2.45E-13	NA	DEg	down
CAP1	4.43E-09	3.62E-10	0.126404	0.639175	3.04E+11	2.12E+10	1	1	0.126404	0.639175	0.197762	-0.70386	0.126404	0.639175	0.197762	67.16899	0.038424	1.38E-05	0.049134	2.43E-05	1.72E-14	2.61E-13	NA	DEg	down
NDUFS7	5.09E-09	1.53E-10	0.188202	0.773196	7.68E+12	4.16E+09	1	1	0.179775	0.773196	0.232509	-0.63356	0.188202	0.773196	0.243408	67.08688	0.010502	1.11E-16	0.014859	5.49E-16	1.80E-14	2.70E-13	NA	DEg	down
PCK1	2.27E-07	2.92E-10	0.109551	0.597938	2.34E+08	4.03E+10	1	1	0.103933	0.597938	0.173818	-0.7599	0.109551	0.597938	0.183214	66.8856	1.45E-05	0.000772	2.98E-05	0.001186	1.98E-14	2.94E-13	NA	DEg	down
FAM177A1	3.24E-09	1.63E-10	0.19382	0.783505	2.59E+13	4.8E+09	1	1	0.19382	0.783505	0.247376	-0.60664	0.19382	0.783505	0.247376	66.86892	9.69E-14	0	4.64E-13	0	2.00E-14	2.95E-13	NA	DEg	down
CFDP1	5.44E-09	1.49E-10	0.199438	0.793814	5.28E+12	4.31E+09	1	1	0.191011	0.793814	0.240625	-0.61866	0.199438	0.793814	0.25124	66.63487	0.001075	3.01E-10	0.001746	8.24E-10	2.24E-14	3.25E-13	NA	DEg	down
HMGCS1	5.44E-09	1.49E-10	0.199438	0.793814	5.28E+12	4.31E+09	1	1	0.191011	0.793814	0.240625	-0.61866	0.199438	0.793814	0.25124	66.63487	0.001075	3.01E-10	0.001746	8.24E-10	2.24E-14	3.25E-13	NA	DEg	down
SCNN1A	0.55734	2.39E-10	0.564767	0.670103	11114.69	9.14E+09	0.999949	1	0.191011	0.670103	0.285048	-0.54508	0.25	0.670103	0.373077	66.60717	4.86E-07	6.70E-09	1.16E-06	1.67E-08	2.28E-14	3.27E-13	NA	DEg	down
IQGAP1	1.58E-09	2.26E-10	0.210674	0.814433	2.37E+13	2.5E+09	1	1	0.210674	0.814433	0.258676	-0.58724	0.210674	0.814433	0.258676	66.30564	2.97E-07	0	7.14E-07	0	2.63E-14	3.75E-13	NA	DEg	down
PAK1	4.36E-09	2.45E-10	0.13764	0.659794	7.80E+11	1.08E+10	1	1	0.13764	0.659794	0.208611	-0.68066	0.13764	0.659794	0.208611	66.23357	0.017176	3.40E-08	0.023338	7.76E-08	2.73E-14	3.86E-13	NA	DEg	down
ARF4	2.09E-09	2.05E-10	0.205056	0.804124	6.69E+13	2.81E+09	1	1	0.202247	0.804124	0.251513	-0.59944	0.205056	0.804124	0.255006	66.18864	0.000592	0	0.000982	0	2.80E-14	3.92E-13	NA	DEg	down
UQCRC1	4.09E-09	1.53E-10	0.191011	0.773196	3.90E+12	4.16E+09	1	1	0.185393	0.773196	0.239775	-0.6202	0.191011	0.773196	0.247041	66.05246	1.46E-12	0	5.72E-12	0	2.99E-14	4.15E-13	NA	DEg	down
BHLHE40	4.74E-09	2.39E-10	0.143258	0.670103	1.05E+12	9.14E+09	1	1	0.143258	0.670103	0.213786	-0.67002	0.143258	0.670103	0.213786	65.81408	0.037798	3.00E-06	0.048473	5.67E-06	3.36E-14	4.63E-13	NA	DEg	down
PPP1CA	3.50E-09	2.50E-10	0.134831	0.649485	8.49E+11	1.29E+10	1	1	0.134831	0.649485	0.207598	-0.68278	0.134831	0.649485	0.207598	65.47489	1.18E-08	3.46E-07	3.14E-08	7.17E-07	3.97E-14	5.42E-13	NA	DEg	down
LINC0048:	7.98E-09	5.22E-10	0.095506	0.556701	5.92E+09	3.16E+10	1	1	0.095506	0.556701	0.171556	-0.76559	0.095506	0.556701	0.171556	65.40123	7.35E-06	0.000116	1.55E-05	0.000191	4.12E-14	5.47E-13	NA	DEg	down
ST14	7.98E-09	5.22E-10	0.095506	0.556701	5.92E+09	3.16E+10	1	1	0.095506	0.556701	0.171556	-0.76559	0.095506	0.556701	0.171556	65.40123	7.35E-06	0.000116	1.55E-05	0.000191	4.12E-14	5.47E-13	NA	DEg	down
CDC42EP5	3.29E-09	2.26E-10	0.213483	0.814433	1.98E+13	2.5E+09	1	1	0.210674	0.814433	0.258676	-0.58724	0.213483	0.814433	0.262125	65.38656	1.35E-13	0	6.23E-13	0	4.14E-14	5.47E-13	NA	DEg	down
FAM96B	3.29E-09	2.26E-10	0.213483	0.814433	1.98E+13	2.5E+09	1	1	0.210674	0.814433	0.258676	-0.58724	0.213483	0.814433	0.262125	65.38656	1.35E-13	0	6.23E-13	0	4.14E-14	5.47E-13	NA	DEg	down
CAPG	4.43E-09	2.76E-10	0.126404	0.628866	3.04E+11	3.14E+10	1	1	0.126404	0.628866	0.201004	-0.6968	0.126404	0.628866	0.201004	65.18994	0.036866	2.80E-06	0.047346	5.32E-06	4.56E-14	5.98E-13	NA	DEg	down
NDUFV2	3.24E-09	1.53E-10	0.19382	0.773196	2.59E+13	4.16E+09	1	1	0.19382	0.773196	0.250674	-0.60089	0.19382	0.773196	0.250674	65.08566	0.000305	0	0.000526	0	4.81E-14	6.25E-13	NA	DEg	down
MLEC	4.98E-09	2.45E-10	0.140449	0.659794	7.98E+11	1.08E+10	1	1	0.140449	0.659794	0.212869	-0.67189	0.140449	0.659794	0.212869	65.05254	0.021124	4.97E-09	0.028272	1.26E-08	4.88E-14	6.30E-13	NA	DEg	down
NET1	5.80E-09	2.80E-10	0.117978	0.608247	9.61E+10	3.89E+10	1	1	0.117978	0.608247	0.193963	-0.71228	0.117978	0.608247	0.193963	64.96237	2.50E-05	9.94E-06	4.95E-05	1.77E-05	5.11E-14	6.51E-13	NA	DEg	down
CD59	1.72E-07	1.73E-10	0.174157	0.731959	2.59E+11	5.77E+09	1	1	0.160112	0.731959	0.218745	-0.66006	0.174157	0.731959	0.237933	64.95559	6.52E-09	7.74E-08	1.75E-08	1.70E-07	5.13E-14	6.51E-13	NA	DEg	down
PRAP1	0.488866	1.67E-10	0.494604	0.835052	14667.73	3.74E+09	0.999966	1	0.224719	0.835052	0.269108	-0.57007	0.252809	0.835052	0.302747	64.93497	2.46E-05	7.19E-12	4.88E-05	2.25E-11	5.18E-14	6.53E-13	NA	DEg	down
SH3GLB1	2.46E-09	1.63E-10	0.199438	0.783505	7.09E+13	4.8E+09	1	1	0.196629	0.783505	0.250961	-0.60039	0.199438	0.783505	0.254546	64.8783	1.73E-06	7.54E-13	3.89E-06	2.51E-12	5.33E-14	6.66E-13	NA	DEg	down
DYNLT1	2.54E-09	2.17E-10	0.160112	0.701031	8.13E+12	5.9E+09	1	1	0.160112	0.701031	0.228396	-0.64131	0.160112	0.701031	0.228396	64.74933	6.66E-08	1.54E-10	1.70E-07	4.28E-10	5.67E-14	6.94E-13	NA	DEg	down
UFM1	2.54E-09	2.17E-10	0.160112	0.701031	8.13E+12	5.9E+09	1	1	0.160112	0.701031	0.228396	-0.64131	0.160112	0.701031	0.228396	64.74933	6.66E-08	1.54E-10	1.70E-07	4.28E-10	5.67E-14	6.94E-13	NA	DEg	down
PTBP1	2.07E-09	1.49E-10	0.205056	0.793814	2.42E+13	4.31E+09	1	1	0.205056	0.793814	0.258318	-0.58785	0.205056	0.793814	0.258318	64.74422	6.32E-12	0	2.33E-11	0	5.68E-14	6.94E-13	NA	DEg	down
TFRC	3.20E-09	1.49E-10	0.205056	0.793814	1.02E+13	4.31E+09	1	1	0.196629	0.793814	0.247702	-0.60607	0.205056	0.793814	0.258318	64.67123	0.000524	1.51E-11	0.000883	4.60E-11	5.90E-14	7.14E-13	NA	DEg	down
PRSS8	5.56E-09	2.04E-10	0.16573	0.71134	2.41E+12	5.78E+09	1	1	0.160112	0.71134	0.225085	-0.64765	0.16573	0.71134	0.232983	64.4586	0.011852	8.55E-15	0.016691	3.34E-14	6.55E-14	7.88E-13	NA	DEg	down
PDLIM5	4.18E-09	2.94E-10	0.123596	0.618557	2.55E+11	3.23E+10	1	1	0.123596	0.618557	0.199813	-0.69938	0.123596	0.618557	0.199813	64.44248	1.80E-05	3.87E-07	3.65E-05	7.96E-07	6.61E-14	7.89E-13	NA	DEg	down
RNF7	4.36E-09	2.50E-10	0.13764	0.649485	7.80E+11	1.29E+10	1	1	0.13764	0.649485	0.211923	-0.67382	0.13764	0.649485	0.211923	64.29402	0.00536	2.35E-06	0.008018	4.53E-06	7.11E-14	8.42E-13	NA	DEg	down
DAZAP2	2.62E-09	2.33E-10	0.227528	0.835052	1.09E+13	1.74E+09	1	1	0.224719	0.835052	0.269108	-0.57007	0.227528	0.835052	0.272472	64.17212	1.12E-11	0	4.02E-11	0	7.54E-14	8.87E-13	NA	DEg	down
MRPL33	4.13E-09	2.76E-10	0.129213	0.628866	4.27E+11	3.14E+10	1	1	0.129213	0.628866	0.205471	-0.68725	0.129213	0.628866	0.205471	63.9665	8.73E-06	1.81E-06	1.82E-05	3.53E-06	8.35E-14	9.71E-13	NA	DEg	down
CYB5R3	6.88E-09	5.22E-10	0.098315	0.556701	9.78E+09	3.16E+10	1	1	0.098315	0.556701	0.176602	-0.753	0.098315	0.556701	0.176602	63.9602	0.000314	1.73E-05	0.00054	3.02E-05	8.37E-14	9.71E-13	NA	DEg	down
CALM3	4.74E-09	2.45E-10	0.143258	0.659794	1.05E+12	1.08E+10	1	1	0.143258	0.659794	0.217126	-0.66329	0.143258	0.659794	0.217126	63.89707	0.000244	4.33E-06	0.000429	7.97E-06	8.64E-14	9.95E-13	NA	DEg	down
MPST	0.230089	1.71E-10	0.251744	0.762887	14047.57	4.19E+09	0.999982	1	0.171348	0.762887	0.224605	-0.64858	0.19382	0.762887	0.254062	63.88104	5.51E-05	1.46E-09	0.000105	3.88E-09	8.70E-14	9.95E-13	NA	DEg	down
CANX	1.28E-09	1.39E-10	0.255618	0.886598	5.31E+10	2.4E+09	1	1	0.255618	0.886598	0.288313	-0.54014	0.255618	0.886598	0.288313	63.70521	1.67E-15	1.20E-11	1.00E-14	3.68E-11	9.49E-14	1.08E-12	NA	DEg	down
LDLR	1.63E-09	1.49E-10	0.207865	0.793814	2.82E+13	4.31E+09	1	1	0.207865	0.793814	0.261856	-0.58194	0.207865	0.793814	0.261856	63.69406	5.25E-08	0	1.36E-07	0	9.55E-14	1.08E-12	NA	DEg	down
WDR45B	2.31E-09	1.92E-10	0.188202	0.752577	6.29E+13	3.96E+09	1	1	0.188202	0.752577	0.250077	-0.60193	0.188202	0.752577	0.250077	63.55531	0.04195	0	0.052958	0	1.02E-13	1.14E-12	NA	DEg	down
PEBP1	1.59E-09	2.33E-10	0.230337	0.835052	1.85E+13	1.74E+09	1	1	0.227528	0.835052	0.272472	-0.56468	0.230337	0.835052	0.275836	63.18075	6.60E-12	0	2.42E-11	0	1.23E-13	1.36E-12	NA	DEg	down
APLP2	3.13E-09	1.71E-10	0.19382	0.762887	1.47E+14	4.19E+09	1	1	0.191011	0.762887	0.25038	-0.6014	0.19382	0.762887	0.254062	63.17878	6.03E-05	0	0.000114	0	1.23E-13	1.36E-12	NA	DEg	down
RSL24D1	2.50E-09	2.05E-10	0.216292	0.804124	4.68E+13	2.81E+09	1	1	0.210674	0.804124	0.261992	-0.58171	0.216292	0.804124	0.268979	62.77919	4.16E-13	0	1.83E-12	0	1.50E-13	1.63E-12	NA	DEg	down
HBA2	3.91E-09	1.49E-10	0.210674	0.793814	3.70E+13	4.31E+09	1	1	0.205056	0.793814	0.258318	-0.58785	0.210674	0.793814	0.265395	62.65472	2.47E-06	0	5.46E-06	0	1.59E-13	1.72E-12	NA	DEg	down
MRPL20	3.30E-09	1.92E-10	0.191011	0.752577	3.00E+13	3.96E+09	1	1	0.191011	0.752577	0.253809	-0.59549	0.191011	0.752577	0.253809	62.39642	1.45E-09	4.12E-14	4.14E-09	1.51E-13	1.81E-13	1.94E-12	NA	DEg	down
CCL20	3.16E-09	2.33E-10	0.233146	0.835052	1.97E+13	1.74E+09	1	1	0.224719	0.835052	0.269108	-0.57007	0.233146	0.835052	0.2792	62.31207	6.25E-14	1.41E-10	3.07E-13	3.96E-10	1.88E-13	2.00E-12	NA	DEg	down

MISP	3.16E-09	2.33E-10	0.233146	0.835052	1.97E+13	1.74E+09	1	1	0.224719	0.835052	0.269108	-0.57007	0.233146	0.835052	0.2792	62.31207	6.25E-14	1.41E-10	3.07E-13	3.96E-10	1.88E-13	2.00E-12	NA	DEg	down
SCAF11	1.12E-08	1.49E-10	0.213483	0.793814	2.04E+12	4.31E+09	1	1	0.196629	0.793814	0.247702	-0.60607	0.213483	0.793814	0.268933	61.84456	1.00E-07	7.74E-14	2.49E-07	2.74E-13	2.37E-13	2.50E-12	NA	DEg	down
PLS1	2.67E-09	2.05E-10	0.219101	0.804124	2.63E+14	2.81E+09	1	1	0.210674	0.804124	0.261992	-0.58171	0.219101	0.804124	0.272472	61.83315	3.85E-13	1.47E-09	1.70E-12	3.92E-09	2.38E-13	2.50E-12	NA	DEg	down
LRP10	3.31E-09	2.25E-10	0.162921	0.690722	6.20E+12	6.89E+09	1	1	0.162921	0.690722	0.235871	-0.62733	0.162921	0.690722	0.235871	61.81686	2.16E-11	1.89E-15	7.54E-11	7.96E-15	2.40E-13	2.50E-12	NA	DEg	down
EMP1	4.28E-09	2.26E-10	0.224719	0.814433	3.19E+13	2.5E+09	1	1	0.216292	0.814433	0.265574	-0.57581	0.224719	0.814433	0.275921	61.72175	4.40E-10	3.03E-11	1.30E-09	9.06E-11	2.52E-13	2.59E-12	NA	DEg	down
HNRNPM	4.28E-09	2.26E-10	0.224719	0.814433	3.19E+13	2.5E+09	1	1	0.216292	0.814433	0.265574	-0.57581	0.224719	0.814433	0.275921	61.72175	4.40E-10	3.03E-11	1.30E-09	9.06E-11	2.52E-13	2.59E-12	NA	DEg	down
ZFAND2A	4.01E-09	2.17E-10	0.168539	0.701031	3.36E+13	5.9E+09	1	1	0.168539	0.701031	0.240416	-0.61904	0.168539	0.701031	0.240416	61.59373	1.05E-07	6.66E-16	2.61E-07	2.99E-15	2.68E-13	2.74E-12	NA	DEg	down
TAPBP	5.05E-09	2.40E-10	0.230337	0.824742	1.48E+13	2.3E+09	1	1	0.216292	0.824742	0.262254	-0.58128	0.230337	0.824742	0.279284	61.57111	2.15E-07	0	5.22E-07	0	2.71E-13	2.75E-12	NA	DEg	down
ITGA2	4.98E-09	3.62E-10	0.140449	0.639175	7.98E+11	2.12E+10	1	1	0.140449	0.639175	0.219735	-0.6581	0.140449	0.639175	0.219735	61.22525	0.000472	4.24E-06	0.000798	7.82E-06	3.22E-13	3.25E-12	NA	DEg	down
EIF3H	1.07E-09	1.25E-10	0.275281	0.907216	6.50E+11	3.42E+09	1	1	0.275281	0.907216	0.303435	-0.51793	0.275281	0.907216	0.303435	61.16396	0	0	0	0	3.32E-13	3.32E-12	NA	DEg	down
CLDN18	8.50E-10	0.628587	0.893258	0.638405	4.88E+09	11572.27	1	0.999945	0.688202	0.237113	2.902418	0.46276	0.893258	0.237113	3.76722	61.0491	0.000372	2.96E-10	0.000633	8.16E-10	3.51E-13	3.50E-12	NA	DEg	up
TMCO1	5.80E-09	3.05E-10	0.117978	0.587629	9.61E+10	4.14E+10	1	1	0.117978	0.587629	0.200769	-0.6973	0.117978	0.587629	0.200769	60.97898	6.01E-07	7.75E-06	1.42E-06	1.40E-05	3.63E-13	3.60E-12	NA	DEg	down
LAMTOR5	2.09E-09	1.53E-10	0.205056	0.773196	6.69E+13	4.16E+09	1	1	0.202247	0.773196	0.261573	-0.58241	0.205056	0.773196	0.265206	60.96131	1.88E-11	0	6.58E-11	0	3.66E-13	3.60E-12	NA	DEg	down
CEBPB	2.65E-09	2.26E-10	0.227528	0.814433	2.28E+13	2.5E+09	1	1	0.22191	0.814433	0.272472	-0.56468	0.227528	0.814433	0.27937	60.81359	1.16E-12	0	4.65E-12	0	3.94E-13	3.85E-12	NA	DEg	down
KRAS	7.98E-09	5.81E-10	0.092697	0.525773	4.33E+09	2.87E+10	1	1	0.092697	0.525773	0.176305	-0.75373	0.092697	0.525773	0.176305	60.63119	8.27E-07	2.60E-05	1.94E-06	4.49E-05	4.31E-13	4.18E-12	NA	DEg	down
ITGA6	6.70E-09	5.91E-10	0.08427	0.505155	9.65E+09	2.4E+10	1	1	0.08427	0.505155	0.16682	-0.77775	0.08427	0.505155	0.16682	60.62354	1.45E-05	1.08E-06	2.98E-05	2.16E-06	4.33E-13	4.18E-12	NA	DEg	down
PSMB1	1.28E-09	1.36E-10	0.255618	0.865979	5.31E+10	2.27E+09	1	1	0.255618	0.865979	0.295178	-0.52992	0.255618	0.865979	0.295178	60.45189	2.22E-16	0	1.43E-15	0	4.71E-13	4.52E-12	NA	DEg	down
UPP1	9.98E-09	5.59E-10	0.073034	0.474227	2.03E+09	1.54E+10	1	1	0.073034	0.474227	0.154006	-0.81246	0.073034	0.474227	0.154006	60.22167	5.09E-05	2.04E-07	9.73E-05	4.33E-07	5.27E-13	5.04E-12	NA	DEg	down
CAPZA1	2.29E-09	1.89E-10	0.182584	0.721649	8.99E+13	5.6E+09	1	1	0.182584	0.721649	0.25301	-0.59686	0.182584	0.721649	0.25301	60.14388	0.048279	1.11E-16	0.060349	5.49E-16	5.48E-13	5.20E-12	NA	DEg	down
PFDN1	5.46E-09	5.22E-10	0.106742	0.556701	3.07E+10	3.16E+10	1	1	0.106742	0.556701	0.191739	-0.71729	0.106742	0.556701	0.191739	60.01872	6.23E-06	5.49E-06	1.32E-05	1.00E-05	5.82E-13	5.50E-12	NA	DEg	down
AP2B1	1.59E-09	2.26E-10	0.230337	0.814433	1.85E+13	2.5E+09	1	1	0.227528	0.814433	0.27937	-0.55382	0.230337	0.814433	0.282819	59.83775	1.60E-12	0	6.24E-12	0	6.37E-13	5.98E-12	NA	DEg	down
SLC2A1	3.31E-08	8.74E-10	0.025281	0.329897	378924	1.99E+10	1	1	0.025281	0.329897	0.076633	-1.11559	0.025281	0.329897	0.076633	59.81711	0.004431	0.039306	0.006695	0.050046	6.43E-13	6.00E-12	NA	DEg	down
SMIM26	2.47E-09	1.53E-10	0.207865	0.773196	1.70E+14	4.16E+09	1	1	0.202247	0.773196	0.261573	-0.58241	0.207865	0.773196	0.268839	59.79079	8.39E-11	1.11E-16	2.67E-10	5.49E-16	6.52E-13	6.05E-12	NA	DEg	down
SYAP1	1.72E-09	2.33E-10	0.241573	0.835052	1.75E+13	1.74E+09	1	1	0.235955	0.835052	0.282563	-0.54888	0.241573	0.835052	0.289291	59.72388	3.44E-15	0	1.98E-14	0	6.73E-13	6.21E-12	NA	DEg	down
PTMS	1.91E-09	1.17E-10	0.247191	0.845361	1.92E+13	3.24E+09	1	1	0.238764	0.845361	0.28244	-0.54907	0.247191	0.845361	0.292409	59.66779	4.12E-12	0	1.54E-11	0	6.92E-13	6.35E-12	NA	DEg	down
SNX3	2.72E-09	1.92E-10	0.199438	0.752577	2.36E+13	3.96E+09	1	1	0.199438	0.752577	0.265007	-0.57674	0.199438	0.752577	0.265007	59.5591	1.67E-09	0	4.74E-09	0	7.30E-13	6.66E-12	NA	DEg	down
TSC22D1	3.98E-09	2.17E-10	0.174157	0.701031	1.04E+14	5.9E+09	1	1	0.174157	0.701031	0.24843	-0.6048	0.174157	0.701031	0.24843	59.3744	9.06E-08	9.99E-16	2.28E-07	4.34E-15	8.00E-13	7.25E-12	NA	DEg	down
SLC6A8	2.12E-08	1.63E-10	0.216292	0.783505	8.91E+12	4.8E+09	1	1	0.196629	0.783505	0.250961	-0.60039	0.216292	0.783505	0.276057	59.28354	2.31E-09	9.99E-16	6.46E-09	4.34E-15	8.36E-13	7.54E-12	NA	DEg	down
MYH9	5.80E-09	2.85E-10	0.117978	0.57732	9.61E+10	5.01E+10	1	1	0.117978	0.57732	0.204354	-0.68962	0.117978	0.57732	0.204354	59.0227	8.66E-08	3.38E-06	2.19E-07	6.33E-06	9.51E-13	8.48E-12	NA	DEg	down
NAPRT	4.27E-09	2.26E-10	0.233146	0.814433	3.64E+13	2.5E+09	1	1	0.216292	0.814433	0.265574	-0.57581	0.233146	0.814433	0.286268	58.95341	1.27E-10	3.07E-08	3.94E-10	7.09E-08	9.84E-13	8.73E-12	NA	DEg	down
YWHAH	1.45E-09	1.66E-10	0.255618	0.85567	1.07E+11	1.95E+09	1	1	0.252809	0.85567	0.295451	-0.52951	0.255618	0.85567	0.298734	58.84326	4.44E-16	0	2.78E-15	0	1.04E-12	9.16E-12	NA	DEg	down
MALL	6.48E-09	1.49E-10	0.224719	0.793814	8.66E+12	4.31E+09	1	1	0.207865	0.793814	0.261856	-0.58194	0.224719	0.793814	0.283088	58.31153	1.48E-10	9.61E-12	4.56E-10	2.97E-11	1.35E-12	1.18E-11	NA	DEg	down
BRK1	2.17E-09	1.49E-10	0.224719	0.793814	3.47E+12	4.31E+09	1	1	0.224719	0.793814	0.283088	-0.54808	0.224719	0.793814	0.283088	58.30673	2.57E-11	0	8.79E-11	0	1.35E-12	1.18E-11	NA	DEg	down
HSD17B11	7.98E-09	5.91E-10	0.092697	0.515464	4.33E+09	2.65E+10	1	1	0.092697	0.515464	0.179831	-0.74513	0.092697	0.515464	0.179831	58.28	0.000102	2.10E-09	0.000188	5.51E-09	1.37E-12	1.19E-11	NA	DEg	down
RPL17	0.165043	0	0.921799	1.649499	17203.32	290.0092	0.999946	0.994344	0.671348	1.649485	0.407005	-0.3904	0.769663	1.649485	0.466608	58.06436	0	8.04E-06	0	1.45E-05	1.52E-12	1.32E-11	NA	DEg	down
ZG16B	5.46E-09	5.44E-10	0.106742	0.546392	3.07E+10	3.15E+10	1	1	0.106742	0.546392	0.195357	-0.70917	0.106742	0.546392	0.195357	58.04525	1.01E-06	4.26E-11	2.34E-06	1.26E-10	1.54E-12	1.32E-11	NA	DEg	down
RAB5IF	1.96E-09	1.66E-10	0.258427	0.85567	1.48E+13	1.95E+09	1	1	0.247191	0.85567	0.288886	-0.53927	0.258427	0.85567	0.302017	58.01274	4.22E-15	0	2.39E-14	0	1.56E-12	1.34E-11	NA	DEg	down
RTN4	8.72E-09	5.91E-10	0.089888	0.505155	2.82E+09	2.4E+10	1	1	0.089888	0.505155	0.177941	-0.74972	0.089888	0.505155	0.177941	57.99322	0.000102	8.01E-05	0.000188	0.000135	1.58E-12	1.34E-11	NA	DEg	down
TMPRSS2	8.82E-09	5.44E-10	0.106742	0.546392	1E+10	3.15E+10	1	1	0.103933	0.546392	0.190216	-0.72075	0.106742	0.546392	0.195357	57.96983	3.92E-06	1.36E-10	8.49E-06	3.85E-10	1.60E-12	1.35E-11	NA	DEg	down
ATP1A1	2.80E-09	2.26E-10	0.233146	0.814433	2.38E+14	2.5E+09	1	1	0.22191	0.814433	0.272472	-0.56468	0.233146	0.814433	0.286268	57.94261	2.43E-11	0	8.35E-11	0	1.62E-12	1.36E-11	NA	DEg	down
MORF4L2	3.50E-09	2.80E-10	0.134831	0.608247	8.49E+11	3.89E+10	1	1	0.134831	0.608247	0.221672	-0.65429	0.134831	0.608247	0.221672	57.78914	4.17E-05	7.76E-13	8.01E-05	2.58E-12	1.74E-12	1.46E-11	NA	DEg	down
ALDH2	4.01E-09	5.22E-10	0.11236	0.556701	9.07E+10	3.16E+10	1	1	0.11236	0.556701	0.201831	-0.69501	0.11236	0.556701	0.201831	57.47909	0.003819	2.36E-08	0.00582	5.53E-08	2.03E-12	1.69E-11	NA	DEg	down
TOMM20	4.98E-09	2.94E-10	0.140449	0.618557	7.98E+11	3.23E+10	1	1	0.140449	0.618557	0.22706	-0.64386	0.140449	0.618557	0.22706	57.4559	0.0012	8.70E-06	0.001945	1.57E-05	2.05E-12	1.70E-11	NA	DEg	down
ITM2C	6.70E-09	5.65E-10	0.08427	0.484536	9.65E+09	1.86E+10	1	1	0.08427	0.484536	0.173918	-0.75965	0.08427	0.484536	0.173918	57.4167	1.30E-05	0.001008	2.70E-05	0.001538	2.09E-12	1.72E-11	NA	DEg	down
CXCL2	2.15E-09	1.63E-10	0.22191	0.783505	4.36E+13	4.8E+09	1	1	0.216292	0.783505	0.276057	-0.559	0.22191	0.783505	0.283227	57.41218	3.46E-11	0	1.15E-10	0	2.10E-12	1.72E-11	NA	DEg	down
CHMP2B	4.43E-09	3.05E-10	0.126404	0.587629	3.04E+11	4.14E+10	1	1	0.126404	0.587629	0.215109	-0.66734	0.126404	0.587629	0.215109	57.39802	1.60E-07	3.80E-08	3.93E-07	8.60E-08	2.11E-12	1.72E-11	NA	DEg	down
FLNB	0.004147	2.05E-10	0.234117	0.804124	20251.83	2.81E+09	0.999988	1	0.207865	0.804124	0.258499	-0.58754	0.233146	0.804124	0.289938	57.38587	1.83E-08	1.55E-15	4.86E-08	6.59E-15	2.13E-12	1.73E-11	NA	DEg	down
SEC62	5.11E-09	2.40E-10	0.244382	0.824742	1.14E+14	2.3E+09	1	1	0.230337	0.824742	0.279284	-0.55395	0.244382	0.824742	0.296313	57.37158	7.28E-12	0	2.66E-11	0	2.14E-12	1.73E-11	NA	DEg	down
PLCG2	0.379473	1.48E-10	1.638693	0.865979	17856.63	3.25E+09	0.999908	1	0.766854	0.865979	0.885534	-0.05279	1.016854	0.865979	1.174224	57.34435	0	3.33E-16	0	1.57E-15	2.17E-12	1.74E-11	NA	DEg	up
TOP1	2.51E-09	1.36E-10	0.266854	0.865979	9.42E+12	2.27E+09	1	1	0.252809	0.865979	0.291934	-0.53472	0.266854	0.865979	0.308153	57.2447	1.07E-13	0	5.05E-13	0	2.28E-12	1.82E-11	NA	DEg	down
C4orf3	2.73E-09	1.17E-10	0.255618	0.845361	1.07E+13	3.24E+09	1	1	0.244382	0.845361	0.289086	-0.53897	0.255618	0.845361	0.302377	57.21007	7.44E-15	0	4.11E-14	0	2.32E-12	1.85E-11	NA	DEg	down
HSPA6	1.29E-08	5.44E-10	0.109551	0.546392	8.7E+09	3.15E+10	1	1	0.106742	0.546392	0.195357	-0.70917	0.109551	0.546392	0.200498	56.69169	2.15E-06	0.000271	4.79E-06	0.000436	2.99E-12	2.37E-11	NA	DEg	down
KIF5B	2.31E-09	2.04E-10	0.188202	0.71134	6.29E+13	5.78E+09	1	1	0.188202	0.71134	0.264574	-0.57745	0.188202	0.71134	0.264574	56.54324	2.40E-08	5.55E-16	6.35E-08	2.51E-15	3.22E-12	2.54E-11	NA	DEg	down
BRI3	2.79E-09	1.39E-10	0.280899	0.886598	2.89E+12	2.4E+09	1	1	0.266854	0.886598	0.300986	-0.52145	0.280899	0.886598	0.316828	56.47423	8.88E-16	0	5.49E-15	0	3.33E-12	2.61E-11	NA	DEg	down
PDXDC1	5.28E-09	2.94E-10	0.143258	0.618557	9.21E+11	3.23E+10	1	1	0.140449	0.618557	0.22706	-0.64386	0.143258	0.618557	0.231601	56.44524	0.041027	2.30E-05	0.051866	3.98E-05	3.38E-12	2.64E-11	NA	DEg	down
FDPS	6.19E-09	5.22E-10	0.115169	0.556701	6.66E+10	3.16E+10	1	1	0.115169	0.556701	0.206877	-0.68429	0.115169	0.556701	0.206877	56.27347	0.000116	5.62E-06	0.000214	1.02E-05	3.67E-12	2.85E-11	NA	DEg	down
DPM3	4.13E-09	3.05E-10	0.129213	0.587629	4.27E+11	4.14E+10	1	1	0.129213	0.587629	0.21989	-0.6578	0.129213	0.587629	0.21989	56.25311	0.025245	2.59E-06	0.033385	4.95E-06	3.71E-12	2.87E-11	NA	DEg	down
OPTN	8.82E-09	5.65E-10	0.106742	0.536082	1E+10	3.05E+10	1	1	0.103933	0.536082	0.193874	-0.71248	0.106742	0.536082	0.199114	55.9119	7.51E-06	1.49E-10	1.58E-05	4.16E-10	4.39E-12	3.38E-11	NA	DEg	down
NAMPT	5.12E-09	1.17E-10	0.258427	0.845361	1.22E+14	3.24E+09	1	1	0.238764	0.845361	0.28244	-0.54907	0.258427	0.845361	0.3057	55.83042	1.14E-13	0	5.37E-13	0	4.57E-12	3.50E-11	NA	DEg	down
ANPEP	0.470907	1.69E-10	0.525597	0.814433	17558.01	4.54E+09	0.99997	1	0.244382	0.814433	0.300064	-0.52279	0.27809	0.814433	0.341452	55.78317	1.16E-09	7.16E-09	3.33E-09	1.78E-08	4.67E-12	3.56E-11	NA	DEg	down
ENY2	1.46E-09	2.26E-10	0.244382	0.814433	1.65E+11	2.5E+09	1	1	0.244382	0.814433	0.300064	-0.52279	0.244382	0.814433	0.300064	55.69491	1.04E-09	1.11E-16	3.01E-09	5.49E-16	4.88E-12	3.70E-11	NA	DEg	down
STARD10	2.27E-09	1.36E-10	0.272472	0.865979	1.60E+12	2.27E+09	1	1	0.264045	0.865979	0.304909	-0.51583	0.272472	0.865979	0.31464	55.67434	1.44E-15	5.85E-13	8.86E-15	1.97E-12	4.93E-12	3.72E-11	NA	DEg	down
MTIF3	4.43E-09	2.85E-10	0.126404	0.57732	3.04E+11	5.01E+10	1	1	0.126404	0.57732	0.218951	-0.65965	0.126404	0.57732	0.218951	55.4865	1.19E-07	1.51E-06	2.96E-07	2.97E-06	5.41E-12	4.06E-11	NA	DEg	down
C4orf48	7.32E-09	1.13E-10	0.317416	0.917526	1.06E+15	3.9E+09	1	1	0.280899	0.917526	0.306148	-0.51407	0.317416	0.917526	0.345947	55.40261	1.21E-14	3.60E-08	6.49E-14	8.19E-08	5.63E-12	4.22E-11	NA	DEg	down
LYPLA1	9.35E-09	5.65E-10	0.087079	0.484536	5.56E+09	1.86E+10	1	1	0.087079	0.484536	0.179716	-0.74541	0.087079	0.484536	0.179716	55.1901	2.13E-05	9.19E-06	4.28E-05	1.64E-05	6.25E-12	4.66E-11	NA	DEg	down
HSPA1B	0.232568	1.92E-10	0.772312	1.134021	12770.24	2.91E+09	0.99994	1	0.446629	1.134021	0.393846	-0.40467	0.592697	1.134021	0.522651	54.95633	3.33E-16	2.44E-15	2.12E-15	1.02E-14	7.02E-12	5.20E-11	NA	DEg	down
MSMO1	7.98E-09	5.91E-10	0.095506	0.505155	5.92E+09	2.4E+10	1	1	0.095506	0.505155	0.189062	-0.7234	0.095506	0.505155	0.189062	54.8987	0.000194	6.96E-07	0.000344	1.42E-06	7.22E-12	5.33E-11	NA	DEg	down
MYH14	0.283694	1.49E-10	0.337249	0.793814	13003.26	4.31E+09	0.999974	1	0.205056	0.793814	0.258318	-0.58785	0.241573	0.793814	0.304319	54.86571	0.018426	6.99E-14	0.024884	2.52E-13	7.33E-12	5.39E-11	NA	DEg	down
TCEAL9	2.07E-09	1.39E-10	0.289326	0.886598	1.54E+12	2.4E+09	1	1	0.286517	0.886598	0.323164	-0.49058	0.289326	0.886598	0.326333	54.24823	0	0	0	0	9.93E-12	7.23E-11	NA	DEg	down
CTSB	7.59E-09	1.66E-10	0.272472	0.85567	5.73E+13	1.95E+09	1	1	0.247191	0.85567	0.288886	-0.53927	0.272472	0.85567	0.318431	54.18017	1.65E-14	0	8.67E-14	0	1.03E-11	7.45E-11	NA	DEg	down
DSC2	1.24E-08	1.49E-10	0.238764	0.793814	1.76E+13	4.31E+09	1	1	0.216292	0.793814	0.272472	-0.56468	0.238764	0.793814	0.300781	54.12829	2.82E-12	8.88E-16	1.07E-11	3.91E-15	1.05E-11	7.60E-11	NA	DEg	down
MXD1	7.41E-08	2.26E-10	0.25	0.814433	1.17E+13	2.5E+09	1	1	0.22191	0.814433	0.272472	-0.56468	0.25	0.814433	0.306962	54.0201	1.84E-09	5.97E-13	5.19E-09	2.01E-12	1.11E-11	7.98E-11	NA	DEg	down
PKP3	0.335537	1.73E-10	0.321287	0.731959	14525.04	5.77E+09	0.999978	1	0.182584	0.731959	0.249446	-0.60302	0.213483	0.731959	0.29166	53.74909	0.032977	3.06E-05	0.042535	5.22E-05	1.27E-11	9.08E-11	NA	DEg	down
SLC35A3	4.01E-09	5.65E-10	0.11236	0.536082	9.07E+10	3.05E+10	1	1	0.11236	0.536082	0.209594	-0.67862	0.11236	0.536082	0.209594	53.59076	1.12E-06	8.94E-09	2.58E-06	2.20E-08	1.37E-11	9.77E-11	NA	DEg	down
HEPH	1.23E-08	5.65E-10	0.11236	0.536082	1.25E+10	3.05E+10	1	1	0.109551	0.536082	0.204354	-0.68962	0.11236	0.536082	0.209594	53.52214	1.85E-06	2.56E-10	4.13E-06	7.06E-10	1.42E-11	1.00E-10	NA	DEg	down
DUSP1	1.60E-09	1.39E-10	0.292135	0.886598	2.02E+12	2.4E+09	1	1	0.27809	0.886598	0.31366	-0.50354	0.292135	0.886598	0.329501	53.52043	0	0	0	0	1.42E-11	1.00E-10	NA	DEg	down
NPW	1.83E-08	0.756265	0.662921	0.549863	1.93E+14	18247.08	1	0.99997	0.567416	0.134021	4.233794	0.62673	0.662921	0.134021	4.946413	53.4853	1.43E-11	1.30E-08	5.08E-11	3.12E-08	1.44E-11	1.02E-10	NA	DEg	up
SELENOS	4.23E-09	1.66E-10	0.275281	0.85567	6.98E+12	1.95E+09	1	1	0.255618	0.85567	0.298734	-0.52471	0.275281	0.85567	0.321714	53.40426	1.27E-14	1.13E-14	6.75E-14	4.36E-14	1.50E-11	1.05E-10	NA	DEg	down
SRRM1	1.82E-09	1.63E-10	0.235955	0.783505	8.00E+12	4.8E+09	1	1	0.233146	0.783505	0.297568	-0.52641	0.235955	0.783505	0.301153	53.35726	4.06E-10	9.99E-16	1.22E-09	4.34E-15	1.54E-11	1.07E-10	NA	DEg	down
ASS1	4.31E-08	2.26E-10	0.252809	0.814433	3.44E+13	2.5E+09	1	1	0.224719	0.814433	0.275921	-0.55922	0.252809	0.814433	0.310411	53.27895	6.33E-10	7.77E-16	1.86E-09	3.48E-15	1.60E-11	1.11E-10	NA	DEg	down
AL603756.	2.86E-09	2.40E-10	0.252809	0.824742	5.76E+14	2.3E+09	1	1	0.235955	0.824742	0.286096	-0.54349	0.252809	0.824742	0.306531	53.16953	9.75E-14	0	4.64E-13	0	1.69E-11	1.17E-10	NA	DEg	down
REG3A	1.39E-09	7.04E-08	0.457865	0.041237	5.21E+11	14475.98	1	0.999997	0.396067	0.041237	9.604635	0.982481	0.457865	0.041237	11.10323	53.13198	2.56E-05	3.33E-08	5.04E-05	7.64E-08	1.72E-11	1.18E-10	NA	DEg	up
PDLIM1	1.62E-09	1.13E-10	0.300562	0.896907	8.43E+12	4.41E+09	1	1	0.294944	0.896907	0.328845	-0.48301	0.300562	0.896907	0.335109	52.86633	0	2.66E-15	0	1.10E-14	1.96E-11	1.34E-10	NA	DEg	down
CXCL8	8.20E-09	2.04E-10	0.199438	0.71134	1.03E+12	5.78E+09	1	1	0.185393	0.71134	0.260625	-0.58398	0.199438	0.71134	0.28037	52.84807	2.87E-10	5.58E-14	8.69E-10	2.03E-13	1.98E-11	1.35E-10	NA	DEg	down
PPP2CB	6.70E-09	6.63E-10	0.08427	0.463918	9.65E+09	3.72E+09	1	1	0.08427	0.463918	0.181648	-0.74077	0.08427	0.463918	0.181648	52.69189	3.38E-06	4.31E-08	7.35E-06	9.66E-08	2.13E-11	1.45E-10	NA	DEg	down
IFITM2	1.95E-09	2.12E-10	0.216292	0.742268	9.61E+12	3.92E+09	1	1	0.216292	0.742268	0.291394	-0.53552	0.216292	0.742268	0.291394	52.6084	3.72E-05	1.11E-16	7.20E-05	5.49E-16	2.22E-11	1.50E-10	NA	DEg	down
PRELID1	0.116007	1.78E-10	0.527484	1.164948	11177.13	2.12E+09	0.999953	1	0.421348	1.164948	0.361688	-0.44167	0.466292	1.164948	0.400268	52.40095	0.000126	0	0.00023	0	2.46E-11	1.66E-10	NA	DEg	down
RNASET2	1.00E-08	7.41E-10	0.067416	0.42268	9.86E+08	8.68E+09	1	1	0.067416	0.42268	0.159496	-0.79725	0.067416	0.42268	0.159496	52.35701	0.000251	0.005911	0.00044	0.008417	2.51E-11	1.69E-10	NA	DEg	down
CYC1	2.87E-09	2.33E-10	0.185393	0.680412	5.32E+13	7.86E+09	1	1	0.185393	0.680412	0.272472	-0.56468	0.185393	0.680412	0.272472	52.30486	2.82E-11	4.11E-15	9.47E-11	1.65E-14	2.58E-11	1.71E-10	NA	DEg	down
MAPK6	3.43E-08	1.40E-09	0.02809	0.309278	889536.3	5.5E+09	1	1	0.02809	0.309278	0.090824	-1.0418	0.02809	0.309278	0.090824	52.30333	0.007131	0.00067	0.010542	0.001033	2.58E-11	1.71E-10	NA	DEg	down
NUDT4	3.43E-08	1.40E-09	0.02809	0.309278	889536.3	5.5E+09	1	1	0.02809	0.309278	0.090824	-1.0418	0.02809	0.309278	0.090824	52.30333	0.007131	0.00067	0.010542	0.001033	2.58E-11	1.71E-10	NA	DEg	down
GPA33	0.16966	3.05E-10	0.169147	0.587629	12535.94	4.14E+10	0.999987	1	0.129213	0.587629	0.21989	-0.6578	0.140449	0.587629	0.23901	52.05952	8.18E-05	0.000524	0.000153	0.00082	2.91E-11	1.92E-10	NA	DEg	down
PGAM1	8.72E-09	5.59E-10	0.089888	0.474227	2.82E+09	1.54E+10	1	1	0.089888	0.474227	0.189546	-0.72229	0.089888	0.474227	0.189546	51.81064	2.31E-05	2.64E-05	4.60E-05	4.54E-05	3.29E-11	2.16E-10	NA	DEg	down
MRPS21	1.15E-09	1.15E-10	0.323034	0.927835	3.14E+11	3.48E+09	1	1	0.323034	0.927835	0.348159	-0.45822	0.323034	0.927835	0.348159	51.69975	0	0	0	0	3.47E-11	2.27E-10	NA	DEg	down
EIF4A1	3.12E-09	1.13E-10	0.317416	0.917526	7.27E+11	3.74E+09	1	1	0.292135	0.917526	0.318394	-0.49703	0.317416	0.917526	0.345947	51.61674	2.22E-16	0	1.43E-15	0	3.61E-11	2.36E-10	NA	DEg	down
ENO1	1.42E-09	1.94E-10	0.525281	1.257732	1.42E+10	1.51E+09	1	1	0.485955	1.257732	0.386374	-0.41299	0.525281	1.257732	0.417641	51.57852	1.30E-11	0	4.63E-11	0	3.68E-11	2.39E-10	NA	DEg	down
PLP2	1.56E-09	1.59E-10	0.294944	0.876289	1.77E+11	1.94E+09	1	1	0.294944	0.876289	0.336583	-0.47291	0.294944	0.876289	0.336583	51.31564	0	3.18E-14	0	1.18E-13	4.19E-11	2.71E-10	NA	DEg	down
PCBD1	5.80E-09	5.65E-10	0.117978	0.536082	9.61E+10	3.05E+10	1	1	0.117978	0.536082	0.220073	-0.65743	0.117978	0.536082	0.220073	51.251	6.58E-07	9.53E-06	1.55E-06	1.70E-05	4.33E-11	2.79E-10	NA	DEg	down
ARF1	1.65E-09	1.26E-10	0.337079	0.948454	4.62E+11	3.3E+09	1	1	0.311798	0.948454	0.328743	-0.48314	0.337079	0.948454	0.355398	51.22256	0	0	0	0	4.39E-11	2.82E-10	NA	DEg	down
VPS28	3.12E-09	1.66E-10	0.283708	0.85567	2.43E+13	1.95E+09	1	1	0.261236	0.85567	0.3053	-0.51527	0.283708	0.85567	0.331562	51.13921	6.63E-14	0	3.24E-13	0	4.57E-11	2.92E-10	NA	DEg	down
GLRX	8.72E-09	6.63E-10	0.089888	0.463918	2.82E+09	3.72E+09	1	1	0.089888	0.463918	0.193758	-0.71274	0.089888	0.463918	0.193758	50.63681	0.006831	1.90E-06	0.010149	3.69E-06	5.85E-11	3.72E-10	NA	DEg	down
NDUFC1	4.50E-09	1.17E-10	0.280899	0.845361	4.43E+13	3.24E+09	1	1	0.258427	0.845361	0.3057	-0.5147	0.280899	0.845361	0.332283	50.62084	2.04E-13	0	9.21E-13	0	5.89E-11	3.72E-10	NA	DEg	down
RAB11A	1.25E-09	1.59E-10	0.297753	0.876289	2.72E+11	1.94E+09	1	1	0.297753	0.876289	0.339789	-0.46879	0.297753	0.876289	0.339789	50.61477	3.44E-15	0	1.98E-14	0	5.91E-11	3.72E-10	NA	DEg	down
IGHG1	2.92E-09	1.59E-10	0.297753	0.876289	1.15E+12	1.94E+09	1	1	0.280899	0.876289	0.320555	-0.4941	0.297753	0.876289	0.339789	50.61279	3.52E-11	0	1.16E-10	0	5.92E-11	3.72E-10	NA	DEg	down
ARPC3	2.25E-09	1.84E-10	0.485955	1.185567	9.39E+11	2.02E+09	1	1	0.441011	1.185567	0.371983	-0.42948	0.485955	1.185567	0.409893	50.34681	0	0	0	0	6.74E-11	4.23E-10	NA	DEg	down
CHMP4B	3.85E-09	1.25E-10	0.317416	0.907216	1.84E+12	3.42E+09	1	1	0.289326	0.907216	0.318916	-0.49632	0.317416	0.907216	0.349879	50.18654	0	0	0	0	7.29E-11	4.55E-10	NA	DEg	down
RER1	1.00E-08	6.60E-10	0.067416	0.412371	9.86E+08	1.08E+10	1	1	0.067416	0.412371	0.163483	-0.78653	0.067416	0.412371	0.163483	50.15962	0.000183	0.01517	0.000327	0.020835	7.39E-11	4.60E-10	NA	DEg	down
DSG2	5.69E-09	1.17E-10	0.280899	0.845361	1.09E+14	3.24E+09	1	1	0.252809	0.845361	0.299055	-0.52425	0.280899	0.845361	0.332283	50.03474	2.20E-12	0	8.48E-12	0	7.85E-11	4.85E-10	NA	DEg	down
BAX	5.22E-09	3.05E-10	0.146067	0.587629	1.17E+12	4.14E+10	1	1	0.143258	0.587629	0.243791	-0.61298	0.146067	0.587629	0.248571	49.87857	1.77E-05	1.96E-08	3.61E-05	4.62E-08	8.48E-11	5.22E-10	NA	DEg	down
ATP5F1B	8.65E-10	1.25E-10	0.367978	0.989691	2.38E+11	9.35E+09	1	1	0.359551	0.989691	0.363296	-0.43974	0.367978	0.989691	0.371811	49.76837	0	2.22E-16	0	1.09E-15	8.95E-11	5.48E-10	NA	DEg	down
GCC2	4.01E-09	5.91E-10	0.11236	0.515464	9.07E+10	2.65E+10	1	1	0.11236	0.515464	0.217978	-0.66159	0.11236	0.515464	0.217978	49.75252	6.69E-06	8.77E-05	1.42E-05	0.000147	9.02E-11	5.51E-10	NA	DEg	down
CDHR5	3.06E-08	6.63E-10	0.089888	0.463918	2.58E+08	3.72E+09	1	1	0.087079	0.463918	0.187703	-0.72653	0.089888	0.463918	0.193758	49.63308	0.018574	0.004502	0.025047	0.006505	9.56E-11	5.82E-10	NA	DEg	down
HNRNPK	1.34E-09	1.03E-10	0.356742	0.969072	1.67E+11	2.87E+09	1	1	0.351124	0.969072	0.36233	-0.4409	0.356742	0.969072	0.368127	49.51992	0	0	0	0	1.01E-10	6.13E-10	NA	DEg	down
RBCK1	1.53E-08	2.80E-10	0.157303	0.608247	4.96E+11	3.89E+10	1	1	0.148876	0.608247	0.244763	-0.61125	0.157303	0.608247	0.258617	49.50314	0.048393	8.18E-07	0.060406	1.65E-06	1.02E-10	6.15E-10	NA	DEg	down
CFLAR	9.35E-09	7.59E-10	0.087079	0.453608	5.56E+09	1.8E+09	1	1	0.087079	0.453608	0.191969	-0.71677	0.087079	0.453608	0.191969	49.3359	2.45E-05	9.21E-05	4.88E-05	0.000154	1.11E-10	6.65E-10	NA	DEg	down
SNU13	1.06E-09	1.26E-10	0.345506	0.948454	2.09E+11	3.3E+09	1	1	0.337079	0.948454	0.355398	-0.44928	0.345506	0.948454	0.364283	49.28453	0	0	0	0	1.13E-10	6.80E-10	NA	DEg	down
CD99	5.52E-09	5.80E-10	0.103933	0.494845	2.29E+10	2.14E+10	1	1	0.103933	0.494845	0.21003	-0.67772	0.103933	0.494845	0.21003	49.24855	7.67E-05	6.12E-06	0.000144	1.11E-05	1.15E-10	6.89E-10	NA	DEg	down
SNHG9	4.74E-09	2.85E-10	0.143258	0.57732	1.05E+12	5.01E+10	1	1	0.143258	0.57732	0.248144	-0.6053	0.143258	0.57732	0.248144	49.08721	1.34E-06	3.02E-05	3.05E-06	5.15E-05	1.25E-10	7.44E-10	NA	DEg	down
ATP5PD	1.10E-09	1.25E-10	0.365169	0.979381	1.66E+11	2.12E+09	1	1	0.359551	0.979381	0.36712	-0.43519	0.365169	0.979381	0.372856	49.02396	0	0	0	0	1.29E-10	7.64E-10	NA	DEg	down
RAB2A	1.15E-09	2.40E-10	0.275281	0.824742	5.45E+10	2.3E+09	1	1	0.272472	0.824742	0.330372	-0.481	0.275281	0.824742	0.333778	48.88061	3.33E-16	5.55E-16	2.12E-15	2.51E-15	1.38E-10	8.17E-10	NA	DEg	down
CBX3	2.77E-09	1.25E-10	0.323034	0.907216	1.87E+12	3.42E+09	1	1	0.294944	0.907216	0.325109	-0.48797	0.323034	0.907216	0.356071	48.85851	2.22E-16	0	1.43E-15	0	1.40E-10	8.23E-10	NA	DEg	down
SMS	1.16E-08	6.60E-10	0.070225	0.412371	1.24E+09	1.08E+10	1	1	0.070225	0.412371	0.170295	-0.7688	0.070225	0.412371	0.170295	48.75124	0.009717	3.17E-06	0.013904	5.97E-06	1.47E-10	8.60E-10	NA	DEg	down
MUC6	1.56E-08	1.84E-10	0.300562	0.865979	1.55E+14	1.77E+09	1	1	0.266854	0.865979	0.308153	-0.51123	0.300562	0.865979	0.347077	48.74483	5.31E-14	2.22E-16	2.66E-13	1.09E-15	1.48E-10	8.60E-10	NA	DEg	down
KDELR1	3.30E-09	2.39E-10	0.191011	0.670103	3.00E+13	9.14E+09	1	1	0.191011	0.670103	0.285048	-0.54508	0.191011	0.670103	0.285048	48.71833	3.60E-11	1.03E-14	1.18E-10	4.01E-14	1.50E-10	8.69E-10	NA	DEg	down
ITGB1	2.86E-09	1.36E-10	0.300562	0.865979	3.14E+12	2.27E+09	1	1	0.27809	0.865979	0.321128	-0.49332	0.300562	0.865979	0.347077	48.48308	2.22E-16	0	1.43E-15	0	1.68E-10	9.71E-10	NA	DEg	down
NDUFA6	4.46E-09	5.81E-10	0.120787	0.525773	1.79E+11	2.87E+10	1	1	0.120787	0.525773	0.229731	-0.63878	0.120787	0.525773	0.229731	48.2663	8.55E-07	1.60E-08	2.00E-06	3.83E-08	1.87E-10	1.08E-09	NA	DEg	down
DHRS7	5.46E-09	5.80E-10	0.106742	0.494845	3.07E+10	2.14E+10	1	1	0.106742	0.494845	0.215707	-0.66614	0.106742	0.494845	0.215707	48.22987	1.82E-05	0.000265	3.67E-05	0.000429	1.90E-10	1.09E-09	NA	DEg	down
POF1B	8.59E-08	5.91E-10	0.11236	0.505155	8.83E+08	2.4E+10	1	1	0.106742	0.505155	0.211305	-0.67509	0.11236	0.505155	0.222426	48.12561	2.09E-05	9.00E-09	4.21E-05	2.20E-08	2.00E-10	1.14E-09	NA	DEg	down
NCOR1	0.394216	5.65E-10	0.171567	0.484536	12350.47	1.86E+10	0.999986	1	0.095506	0.484536	0.197107	-0.7053	0.103933	0.484536	0.214499	48.12327	0.040304	0.002613	0.051024	0.003832	2.00E-10	1.14E-09	NA	DEg	down

CUTA	2.29E-09	2.50E-10	0.182584	0.649485	8.99E+13	1.29E+10	1	1	0.182584	0.649485	0.281122	-0.55111	0.182584	0.649485	0.281122	48.11704	2.47E-10	4.79E-10	7.52E-10	1.30E-09	2.01E-10	1.14E-09	NA	DEg	down
NOP10	1.35E-09	1.25E-10	0.376404	0.989691	1.21E+11	9.35E+09	1	1	0.365169	0.989691	0.368972	-0.43301	0.376404	0.989691	0.380325	47.94422	0	0	0	0	2.19E-10	1.24E-09	NA	DEg	down
UBE2L3	3.54E-09	3.05E-10	0.151685	0.587629	2.93E+12	4.14E+10	1	1	0.151685	0.587629	0.258131	-0.58816	0.151685	0.587629	0.258131	47.91683	1.15E-06	3.68E-12	2.64E-06	1.19E-11	2.22E-10	1.25E-09	NA	DEg	down
SPINT1	0.40463	2.04E-10	0.386881	0.71134	12564.18	5.78E+09	0.999969	1	0.191011	0.71134	0.268523	-0.57102	0.230337	0.71134	0.323807	47.80013	1.75E-06	6.14E-11	3.92E-06	1.79E-10	2.35E-10	1.32E-09	NA	DEg	down
METAP2	3.68E-09	1.17E-10	0.292135	0.845361	4.22E+12	3.24E+09	1	1	0.269663	0.845361	0.318992	-0.49622	0.292135	0.845361	0.345574	47.61894	6.55E-15	4.73E-09	3.67E-14	1.20E-08	2.57E-10	1.43E-09	NA	DEg	down
CTSS	7.98E-09	7.59E-10	0.092697	0.453608	4.33E+09	1.8E+09	1	1	0.092697	0.453608	0.204354	-0.68962	0.092697	0.453608	0.204354	47.40784	0.00475	2.63E-05	0.007165	4.52E-05	2.85E-10	1.58E-09	NA	DEg	down
HEBP2	1.65E-09	1.39E-10	0.317416	0.886598	2.29E+11	2.4E+09	1	1	0.300562	0.886598	0.339006	-0.46979	0.317416	0.886598	0.358015	47.34292	0	5.71E-11	0	1.67E-10	2.94E-10	1.63E-09	NA	DEg	down
CSNK1A1	0.095475	1.71E-10	0.273283	0.762887	15151.59	4.19E+09	0.999982	1	0.216292	0.762887	0.283518	-0.54742	0.247191	0.762887	0.324021	47.2611	8.05E-11	1.22E-13	2.57E-10	4.31E-13	3.06E-10	1.69E-09	NA	DEg	down
HBB	7.98E-09	6.63E-10	0.095506	0.463918	5.92E+09	3.72E+09	1	1	0.095506	0.463918	0.205868	-0.68641	0.095506	0.463918	0.205868	47.14368	2.68E-06	1.32E-07	5.87E-06	2.88E-07	3.24E-10	1.78E-09	NA	DEg	down
SLC44A4	4.33E-08	2.26E-10	0.27809	0.814433	5.45E+13	2.5E+09	1	1	0.244382	0.814433	0.300064	-0.52279	0.27809	0.814433	0.341452	46.82492	1.08E-09	5.55E-16	3.09E-09	2.51E-15	3.79E-10	2.08E-09	NA	DEg	down
MORF4L1	1.58E-09	1.25E-10	0.376404	0.979381	2.06E+12	2.12E+09	1	1	0.351124	0.979381	0.358516	-0.44549	0.376404	0.979381	0.384329	46.61766	0	1.11E-16	0	5.49E-16	4.19E-10	2.29E-09	NA	DEg	down
TMEM54	0.232681	1.14E-10	0.552778	1.030928	10776.25	3.3E+09	0.999949	1	0.367978	1.030928	0.356938	-0.44741	0.424157	1.030928	0.411433	46.5156	1.67E-14	7.69E-09	8.67E-14	1.90E-08	4.41E-10	2.40E-09	NA	DEg	down
TMEM50A	7.98E-09	8.46E-10	0.092697	0.443299	4.33E+09	7.39E+09	1	1	0.092697	0.443299	0.209106	-0.67963	0.092697	0.443299	0.209106	46.4658	7.48E-05	0.00036	0.00014	0.00057	4.51E-10	2.45E-09	NA	DEg	down
CDV3	2.55E-09	1.92E-10	0.244382	0.752577	2.21E+13	3.96E+09	1	1	0.233146	0.752577	0.309797	-0.50892	0.244382	0.752577	0.324727	46.43103	9.02E-13	2.69E-14	3.74E-12	1.01E-13	4.59E-10	2.49E-09	NA	DEg	down
HIGD2A	1.07E-09	1.26E-10	0.359551	0.948454	1.74E+11	3.3E+09	1	1	0.348315	0.948454	0.367245	-0.43504	0.359551	0.948454	0.379091	46.19541	0	3.11E-13	0	1.07E-12	5.15E-10	2.78E-09	NA	DEg	down
STAT6	5.46E-09	5.65E-10	0.106742	0.484536	3.07E+10	1.86E+10	1	1	0.106742	0.484536	0.220296	-0.65699	0.106742	0.484536	0.220296	46.17531	2.53E-05	0.00034	4.99E-05	0.00054	5.20E-10	2.80E-09	NA	DEg	down
TAF10	3.06E-08	1.40E-09	0.036517	0.309278	4283965	5.5E+09	1	1	0.036517	0.309278	0.118071	-0.92786	0.036517	0.309278	0.118071	46.033	0.017423	0.008442	0.023638	0.011777	5.58E-10	2.99E-09	NA	DEg	down
POLR1D	1.23E-09	1.25E-10	0.379213	0.979381	3.44E+10	2.12E+09	1	1	0.373596	0.979381	0.381461	-0.41855	0.379213	0.979381	0.387197	46.02845	0	0	0	0	5.59E-10	2.99E-09	NA	DEg	down
PSMB7	1.00E-08	6.98E-10	0.067416	0.391753	9.86E+08	1.64E+10	1	1	0.067416	0.391753	0.172088	-0.76425	0.067416	0.391753	0.172088	45.96621	0.000163	0.015852	0.000294	0.021672	5.77E-10	3.07E-09	NA	DEg	down
IGHG4	0.347696	0.775309	1.804322	1.51411	17792.19	16100.72	0.999899	0.999906	1.061798	0.340206	3.121042	0.4943	1.176966	0.340206	3.459568	45.7707	2.93E-07	0.002466	7.08E-07	0.003627	6.35E-10	3.37E-09	NA	DEg	up
SPINK4	0.428222	0.761541	2.638135	1.556385	15847.17	22.98063	0.999834	0.93657	1.401685	0.371134	3.776763	0.57712	1.508427	0.371134	4.064373	45.73554	5.79E-13	0.00033	2.45E-12	0.000524	6.46E-10	3.41E-09	NA	DEg	up
CEBPD	6.02E-07	1.92E-10	0.247192	0.752577	5.67E+11	3.96E+09	1	1	0.219101	0.752577	0.291134	-0.53591	0.247191	0.752577	0.328459	45.62856	3.39E-10	2.36E-06	1.02E-09	4.54E-06	6.80E-10	3.57E-09	NA	DEg	down
CD164	3.24E-09	1.17E-10	0.300562	0.845361	3.07E+11	3.24E+09	1	1	0.280899	0.845361	0.332283	-0.47849	0.300562	0.845361	0.355543	45.62651	9.76E-08	0	2.45E-07	0	6.81E-10	3.57E-09	NA	DEg	down
MAP1LC3B	9.61E-09	5.91E-10	0.117978	0.505155	3.06E+10	2.4E+10	1	1	0.115169	0.505155	0.227987	-0.64209	0.117978	0.505155	0.233547	45.62322	6.18E-06	1.68E-07	1.32E-05	3.61E-07	6.82E-10	3.57E-09	NA	DEg	down
EPS8L2	7.51E-09	7.41E-10	0.081461	0.42268	6.62E+09	8.68E+09	1	1	0.081461	0.42268	0.192724	-0.71506	0.081461	0.42268	0.192724	45.40451	0.024099	0.000237	0.031964	0.000385	7.59E-10	3.95E-09	NA	DEg	down
GSTO1	5.52E-09	5.59E-10	0.103933	0.474227	2.29E+10	1.54E+10	1	1	0.103933	0.474227	0.219162	-0.65923	0.103933	0.474227	0.219162	45.34937	0.002092	4.02E-06	0.003278	7.43E-06	7.80E-10	4.03E-09	NA	DEg	down
IFI27L2	5.52E-09	5.59E-10	0.103933	0.474227	2.29E+10	1.54E+10	1	1	0.103933	0.474227	0.219162	-0.65923	0.103933	0.474227	0.219162	45.34937	0.002092	4.02E-06	0.003278	7.43E-06	7.80E-10	4.03E-09	NA	DEg	down
EIF3A	5.85E-09	2.85E-10	0.154494	0.57732	1.93E+12	5.01E+10	1	1	0.151685	0.57732	0.262741	-0.58047	0.154494	0.57732	0.267606	45.32589	7.36E-07	6.23E-08	1.73E-06	1.38E-07	7.89E-10	4.07E-09	NA	DEg	down
ZNF706	1.43E-08	1.17E-10	0.303371	0.845361	9.26E+13	3.24E+09	1	1	0.266854	0.845361	0.315669	-0.50077	0.303371	0.845361	0.358865	45.17617	3.52E-14	8.88E-16	1.79E-13	3.91E-15	8.49E-10	4.35E-09	NA	DEg	down
PTGES3	6.89E-10	1.03E-10	0.379213	0.969072	2.02E+10	2.87E+09	1	1	0.376404	0.969072	0.388417	-0.4107	0.379213	0.969072	0.391316	44.72064	0	0	0	0	1.06E-09	5.42E-09	NA	DEg	down
NDUFB3	4.46E-09	5.91E-10	0.120787	0.505155	1.79E+11	2.4E+10	1	1	0.120787	0.505155	0.239108	-0.62141	0.120787	0.505155	0.239108	44.61104	1.41E-06	3.52E-05	3.19E-06	5.98E-05	1.12E-09	5.70E-09	NA	DEg	down
PKP2	5.46E-09	5.59E-10	0.106742	0.474227	3.07E+10	1.54E+10	1	1	0.106742	0.474227	0.225085	-0.64765	0.106742	0.474227	0.225085	44.50478	3.09E-05	6.77E-05	6.07E-05	0.000114	1.18E-09	5.98E-09	NA	DEg	down
DEK	1.76E-09	1.53E-10	0.264045	0.773196	5.46E+12	4.16E+09	1	1	0.255618	0.773196	0.330599	-0.4807	0.264045	0.773196	0.341498	44.33518	5.47E-13	0	2.35E-12	0	1.28E-09	6.48E-09	NA	DEg	down
C1QBP	3.31E-09	3.05E-10	0.162921	0.587629	6.20E+12	4.14E+10	1	1	0.162921	0.587629	0.277252	-0.55713	0.162921	0.587629	0.277252	44.21663	6.60E-08	2.42E-06	1.69E-07	4.65E-06	1.36E-09	6.85E-09	NA	DEg	down
HNRNPC	1.14E-09	1.39E-10	0.331461	0.886598	3.02E+11	2.4E+09	1	1	0.331461	0.886598	0.373857	-0.42729	0.331461	0.886598	0.373857	44.18467	0	0	0	0	1.38E-09	6.93E-09	NA	DEg	down
ITLN1	0.256355	0.795809	0.853677	0.60586	11639.8	14715.06	0.999927	0.999959	0.542135	0.123711	4.382257	0.641698	0.634831	0.123711	5.131554	44.15118	0.018343	6.91E-06	0.024811	1.25E-05	1.40E-09	7.03E-09	NA	DEg	up
MARCKSL1	1.59E-09	2.04E-10	0.230337	0.71134	1.85E+13	5.78E+09	1	1	0.227528	0.71134	0.319858	-0.49504	0.230337	0.71134	0.323807	43.94258	1.15E-12	1.09E-09	4.64E-12	2.93E-09	1.55E-09	7.76E-09	NA	DEg	down
HBEGF	1.81E-08	8.23E-10	0.058989	0.360825	92103425	2.83E+10	1	1	0.058989	0.360825	0.163483	-0.78653	0.058989	0.360825	0.163483	43.90283	0.000572	0.001939	0.000954	0.002871	1.58E-09	7.88E-09	NA	DEg	down
LAD1	5.80E-09	5.80E-10	0.117978	0.494845	9.61E+10	2.14E+10	1	1	0.117978	0.494845	0.238413	-0.62267	0.117978	0.494845	0.238413	43.83839	8.32E-06	8.04E-07	1.74E-05	1.63E-06	1.63E-09	8.11E-09	NA	DEg	down
CSDE1	1.00E-08	7.52E-10	0.067416	0.381443	9.86E+08	2.02E+10	1	1	0.067416	0.381443	0.176739	-0.75267	0.067416	0.381443	0.176739	43.82957	0.00029	0.010763	0.000503	0.014898	1.64E-09	8.12E-09	NA	DEg	down
DDX24	5.28E-09	5.44E-10	0.143258	0.546392	9.21E+11	3.15E+10	1	1	0.140449	0.546392	0.257049	-0.58998	0.143258	0.546392	0.26219	43.82152	1.32E-06	1.52E-07	3.02E-06	3.29E-07	1.65E-09	8.13E-09	NA	DEg	down
UFC1	3.06E-08	1.64E-09	0.036517	0.298969	4283965	3.02E+09	1	1	0.036517	0.298969	0.122143	-0.91313	0.036517	0.298969	0.122143	43.68101	0.007931	0.029359	0.011553	0.03854	1.76E-09	8.68E-09	NA	DEg	down
NDRG1	0.340112	4.37E-10	1.255745	1.536082	10602.63	1.42E+10	0.999882	1	0.606742	1.536082	0.394993	-0.40341	0.828652	1.536082	0.539458	43.60479	0	0	0	0	1.83E-09	8.98E-09	NA	DEg	down
YBX3	3.93E-09	1.49E-10	0.27809	0.793814	1.67E+14	4.31E+09	1	1	0.255618	0.793814	0.322012	-0.49213	0.27809	0.793814	0.350321	43.49165	7.76E-10	8.13E-07	2.26E-09	1.64E-06	1.94E-09	9.47E-09	NA	DEg	down
PDCD6IP	7.51E-09	6.60E-10	0.081461	0.412371	6.62E+09	1.08E+10	1	1	0.081461	0.412371	0.197542	-0.70434	0.081461	0.412371	0.197542	43.4548	5.76E-05	0.006478	0.000109	0.009151	1.97E-09	9.61E-09	NA	DEg	down
GSTK1	0.296203	2.12E-10	0.367189	0.742268	16413	3.92E+09	0.999978	1	0.216292	0.742268	0.291394	-0.53552	0.258427	0.742268	0.348159	43.40074	6.02E-07	8.94E-06	1.42E-06	1.60E-05	2.02E-09	9.84E-09	NA	DEg	down
MT1G	0.360981	5.91E-09	0.967074	0.103093	12797.25	781101.1	0.999924	1	0.396067	0.103093	3.841854	0.584541	0.617978	0.103093	5.994382	43.32248	3.32E-06	2.14E-07	7.24E-06	4.53E-07	2.10E-09	1.02E-08	NA	DEg	up
SERPINB6	7.85E-09	2.33E-10	0.297753	0.835052	4.29E+14	1.74E+09	1	1	0.266854	0.835052	0.319566	-0.49544	0.297753	0.835052	0.356568	43.30843	1.24E-13	1.11E-15	5.77E-13	4.77E-15	2.12E-09	1.02E-08	NA	DEg	down
C15orf48	0.306029	6.51E-10	1.679796	2.041237	14003.78	5.18E+10	0.99988	1	0.884831	2.041237	0.433478	-0.36303	1.16573	2.041237	0.57109	43.27966	0	0	0	0	2.15E-09	1.03E-08	NA	DEg	down
SDC4	7.91E-10	3.19E-09	0.676966	0.175258	4.59E+09	49600291	1	1	0.561798	0.175258	3.205552	0.505903	0.676966	0.175258	3.86269	43.2178	9.79E-13	5.57E-14	4.04E-12	2.03E-13	2.21E-09	1.06E-08	NA	DEg	up
LPP	6.19E-09	5.65E-10	0.115169	0.484536	6.66E+10	1.86E+10	1	1	0.115169	0.484536	0.237688	-0.62399	0.115169	0.484536	0.237688	43.02234	0.016461	0.000382	0.022574	0.000603	2.43E-09	1.17E-08	NA	DEg	down
ARPC1A	4.46E-09	5.80E-10	0.120787	0.494845	1.79E+11	2.14E+10	1	1	0.120787	0.494845	0.244089	-0.61245	0.120787	0.494845	0.244089	42.83373	1.56E-06	3.77E-08	3.53E-06	8.57E-08	2.67E-09	1.28E-08	NA	DEg	down
CTTN	5.46E-09	6.63E-10	0.106742	0.463918	3.07E+10	3.72E+09	1	1	0.106742	0.463918	0.230087	-0.63811	0.106742	0.463918	0.230087	42.36219	3.53E-06	0.000697	7.67E-06	0.001073	3.36E-09	1.60E-08	NA	DEg	down
HPGD	0.509416	2.25E-10	0.561129	0.690722	13813.16	6.89E+09	0.999959	1	0.210674	0.690722	0.305006	-0.51569	0.275281	0.690722	0.398541	42.35173	1.96E-09	0.002939	5.50E-09	0.004288	3.38E-09	1.60E-08	NA	DEg	down
LSM4	1.26E-09	1.53E-10	0.272472	0.773196	8.94E+10	4.16E+09	1	1	0.269663	0.773196	0.348764	-0.45747	0.272472	0.773196	0.352397	42.30248	3.00E-15	3.77E-09	1.76E-14	9.67E-09	3.46E-09	1.64E-08	NA	DEg	down
SKP1	1.53E-09	1.25E-10	0.404494	0.989691	1.59E+10	9.35E+09	1	1	0.376404	0.989691	0.380325	-0.41984	0.404494	0.989691	0.408708	42.25807	1.16E-11	0	4.16E-11	0	3.54E-09	1.67E-08	NA	DEg	down
FIS1	1.36E-09	1.59E-10	0.33427	0.876289	7.45E+11	1.94E+09	1	1	0.314607	0.876289	0.359022	-0.44488	0.33427	0.876289	0.381461	42.23652	0	0	0	0	3.57E-09	1.68E-08	NA	DEg	down
LY6E	8.21E-10	3.19E-09	0.66573	0.175258	9.47E+09	49600291	1	1	0.558989	0.175258	3.189524	0.503726	0.66573	0.175258	3.798579	41.83976	5.43E-13	4.44E-15	2.34E-12	1.78E-14	4.34E-09	2.04E-08	NA	DEg	up
CHURC1	1.81E-08	8.54E-10	0.058989	0.350515	92103425	2.9E+10	1	1	0.058989	0.350515	0.168291	-0.77394	0.058989	0.350515	0.168291	41.77855	0.000754	0.030002	0.00124	0.03921	4.47E-09	2.09E-08	NA	DEg	down
RNF145	2.46E-09	3.62E-10	0.199438	0.639175	7.09E+13	2.12E+10	1	1	0.196629	0.639175	0.30763	-0.51197	0.199438	0.639175	0.312024	41.57303	8.23E-10	1.71E-08	2.39E-09	4.07E-08	4.94E-09	2.31E-08	NA	DEg	down
UXT	2.15E-09	2.33E-10	0.22191	0.680412	4.36E+13	7.86E+09	1	1	0.216292	0.680412	0.317884	-0.49773	0.22191	0.680412	0.326141	41.56102	8.28E-12	3.41E-14	3.01E-11	1.26E-13	4.97E-09	2.31E-08	NA	DEg	down
TES	9.13E-09	2.05E-10	0.27809	0.804124	3.51E+14	2.81E+09	1	1	0.247191	0.804124	0.307404	-0.51229	0.27809	0.804124	0.34583	41.47441	7.74E-08	5.11E-15	1.97E-07	2.03E-14	5.19E-09	2.41E-08	NA	DEg	down
TMOD3	7.51E-09	6.91E-10	0.081461	0.402062	6.62E+09	1.24E+10	1	1	0.081461	0.402062	0.202607	-0.69334	0.081461	0.402062	0.202607	41.43603	0.000186	0.002307	0.000331	0.003399	5.28E-09	2.45E-08	NA	DEg	down
C12orf57	1.07E-09	1.03E-10	0.396067	0.969072	8.12E+10	2.87E+09	1	1	0.373596	0.969072	0.385519	-0.41395	0.396067	0.969072	0.408708	41.3776	5.65E-07	0	1.34E-06	0	5.44E-09	2.51E-08	NA	DEg	down
MGST3	4.30E-09	2.00E-10	0.502809	1.134021	8.97E+11	2.12E+09	1	1	0.47191	1.134021	0.416139	-0.38076	0.502809	1.134021	0.443386	41.31461	0	0	0	0	5.61E-09	2.58E-08	NA	DEg	down
SPTBN1	4.55E-09	1.36E-10	0.33427	0.865979	1.20E+14	2.27E+09	1	1	0.294944	0.865979	0.34059	-0.46777	0.33427	0.865979	0.386002	41.28757	6.77E-15	0	3.77E-14	0	5.68E-09	2.61E-08	NA	DEg	down
PI3	0.07141	2.33E-10	0.3388	0.835052	10060.57	1.74E+09	0.999966	1	0.266854	0.835052	0.319566	-0.49544	0.314607	0.835052	0.376751	41.20521	1.19E-12	4.64E-07	4.74E-12	9.49E-07	5.92E-09	2.71E-08	NA	DEg	down
UQCRFS1	5.46E-09	7.59E-10	0.106742	0.453608	3.07E+10	1.8E+09	1	1	0.106742	0.453608	0.235317	-0.62835	0.106742	0.453608	0.235317	40.86608	0.009693	7.12E-08	0.013892	1.57E-07	6.98E-09	3.18E-08	NA	DEg	down
HSPH1	7.96E-09	3.05E-10	0.174157	0.587629	2.75E+12	4.14E+10	1	1	0.16573	0.587629	0.282032	-0.5497	0.174157	0.587629	0.296373	40.80684	5.12E-07	1.59E-06	1.22E-06	3.10E-06	7.19E-09	3.27E-08	NA	DEg	down
ERBB3	0.52178	5.81E-10	0.323061	0.525773	11563.21	2.87E+10	0.999972	1	0.132022	0.525773	0.251102	-0.60015	0.154494	0.525773	0.293842	40.79001	4.51E-05	8.82E-07	8.63E-05	1.77E-06	7.25E-09	3.29E-08	NA	DEg	down
ERO1A	9.83E-09	6.98E-10	0.078652	0.391753	3.72E+09	1.64E+10	1	1	0.078652	0.391753	0.200769	-0.6973	0.078652	0.391753	0.200769	40.63489	0.000165	2.06E-06	0.000297	3.99E-06	7.82E-09	3.54E-08	NA	DEg	down
CXADR	7.33E-09	7.52E-10	0.075843	0.381443	3.99E+09	2.02E+10	1	1	0.075843	0.381443	0.198831	-0.70152	0.075843	0.381443	0.198831	39.8845	0.000178	2.74E-06	0.00032	5.21E-06	1.13E-08	5.09E-08	NA	DEg	down
KRT10	4.13E-09	5.80E-10	0.129213	0.494845	4.27E+11	2.14E+10	1	1	0.129213	0.494845	0.261119	-0.58316	0.129213	0.494845	0.261119	39.82659	1.59E-06	4.51E-10	3.59E-06	1.23E-09	1.16E-08	5.22E-08	NA	DEg	down
SPCS2	4.98E-09	5.91E-10	0.140449	0.515464	7.98E+11	2.65E+10	1	1	0.140449	0.515464	0.272472	-0.56468	0.140449	0.515464	0.272472	39.5776	1.94E-07	1.36E-10	4.72E-07	3.85E-10	1.31E-08	5.86E-08	NA	DEg	down
NDUFA12	5.68E-09	7.59E-10	0.109551	0.453608	3.81E+10	1.8E+09	1	1	0.109551	0.453608	0.241509	-0.61707	0.109551	0.453608	0.241509	39.47207	0.014672	6.09E-05	0.020372	0.000103	1.38E-08	6.15E-08	NA	DEg	down
SNRPF	7.20E-10	1.25E-10	0.412921	0.979381	5.22E+09	2.12E+09	1	1	0.407303	0.979381	0.415878	-0.38103	0.412921	0.979381	0.421614	39.44499	0.001615	4.44E-16	0.002563	2.06E-15	1.40E-08	6.21E-08	NA	DEg	down
RHOB	3.55E-09	1.25E-10	0.412921	0.979381	8.02E+11	2.12E+09	1	1	0.353933	0.979381	0.361384	-0.44203	0.412921	0.979381	0.421614	39.44198	1.74E-09	0	4.94E-09	0	1.40E-08	6.21E-08	NA	DEg	down
ASPH	1.62E-09	1.13E-10	0.36236	0.896907	1.60E+11	4.41E+09	1	1	0.33427	0.896907	0.372691	-0.42865	0.36236	0.896907	0.40401	39.11335	0	3.67E-10	0	1.00E-09	1.64E-08	7.27E-08	NA	DEg	down
HNRNPDL	7.19E-10	1.03E-10	0.410112	0.969072	7.88E+09	2.87E+09	1	1	0.401685	0.969072	0.414505	-0.38247	0.410112	0.969072	0.423201	38.74863	9.93E-08	5.55E-16	2.48E-07	2.51E-15	1.96E-08	8.66E-08	NA	DEg	down
KLF4	4.63E-10	6.60E-10	1.02809	0.412371	9.77E+09	1.08E+10	1	1	0.758427	0.412371	1.839185	0.264626	1.02809	0.412371	2.493118	38.35751	0	0	0	0	2.37E-08	1.04E-07	NA	DEg	up
TMC5	0.320374	1.48E-10	1.446597	0.938144	12372	2.3E+09	0.999883	1	0.730337	0.938144	0.778491	-0.10875	0.983146	0.938144	1.047969	38.35157	2.22E-16	3.93E-06	1.43E-15	7.31E-06	2.38E-08	1.05E-07	NA	DEg	up
ADIRF	0.289463	3.51E-10	1.091119	1.371134	17365.22	3.54E+09	0.999937	1	0.564607	1.371134	0.411781	-0.38533	0.775281	1.371134	0.56543	38.18218	1.01E-10	0	3.17E-10	0	2.59E-08	1.13E-07	NA	DEg	down
USP53	0.136118	2.33E-10	0.383687	0.835052	18046.5	1.74E+09	0.999979	1	0.275281	0.835052	0.329657	-0.48194	0.331461	0.835052	0.396934	38.09823	3.35E-12	1.53E-08	1.26E-11	3.67E-08	2.69E-08	1.17E-07	NA	DEg	down
TSPAN3	0.072436	1.66E-10	0.369459	0.85567	10099.29	1.95E+09	0.999963	1	0.286517	0.85567	0.334845	-0.47516	0.342697	0.85567	0.400501	38.04393	7.92E-08	0	2.01E-07	0	2.77E-08	1.20E-07	NA	DEg	down
GNB1	7.33E-09	7.94E-10	0.075843	0.371134	3.99E+09	2.47E+10	1	1	0.075843	0.371134	0.204354	-0.68962	0.075843	0.371134	0.204354	37.94866	0.000137	0.000447	0.000249	0.0007	2.90E-08	1.25E-07	NA	DEg	down
SPINK1	1.38E-10	0	2.353933	1.371135	7.27E+08	414.1735	1	0.9967	2.025281	1.371134	1.477085	0.169405	2.353933	1.371134	1.716778	37.9399	0	5.34E-12	0	1.72E-11	2.91E-08	1.25E-07	NA	DEg	up
ATP5PF	7.37E-10	1.25E-10	0.421348	0.979381	7.16E+09	2.12E+09	1	1	0.410112	0.979381	0.418746	-0.37805	0.421348	0.979381	0.430219	37.9235	0	0	0	0	2.93E-08	1.26E-07	NA	DEg	down
MDH2	3.54E-09	5.81E-10	0.151685	0.525773	2.93E+12	2.87E+10	1	1	0.151685	0.525773	0.2885	-0.53985	0.151685	0.525773	0.2885	37.74887	2.42E-07	1.25E-05	5.86E-07	2.21E-05	3.19E-08	1.37E-07	NA	DEg	down
MTDH	2.89E-09	1.25E-10	0.376404	0.907216	4.34E+11	3.42E+09	1	1	0.33427	0.907216	0.368456	-0.43361	0.376404	0.907216	0.4149	37.66951	1.15E-12	2.35E-14	4.65E-12	8.91E-14	3.32E-08	1.41E-07	NA	DEg	down
EPS8	0.620442	5.44E-10	0.777072	0.546392	16915.4	3.15E+10	0.999954	1	0.205056	0.546392	0.375291	-0.42563	0.294944	0.546392	0.539803	37.66491	0.000364	0.028985	0.000621	0.038104	3.33E-08	1.41E-07	NA	DEg	down
SRGN	8.23E-10	4.33E-09	0.592697	0.154639	8.34E+09	14066667	1	1	0.508427	0.154639	3.287828	0.516909	0.592697	0.154639	3.832772	37.56205	4.22E-10	3.33E-05	1.26E-09	5.67E-05	3.50E-08	1.48E-07	NA	DEg	up
ANAPC16	1.07E-09	2.12E-10	0.275281	0.742268	6.50E+11	3.92E+09	1	1	0.275281	0.742268	0.370865	-0.43078	0.275281	0.742268	0.370865	37.48111	3.84E-08	3.39E-08	1.01E-07	7.75E-08	3.64E-08	1.54E-07	NA	DEg	down
RPS4Y1	7.44E-10	3.43E-09	0.609551	0.164948	2.11E+10	30780812	1	1	0.514045	0.164948	3.116397	0.493653	0.609551	0.164948	3.6954	37.30947	5.08E-09	7.45E-14	1.37E-08	2.66E-13	3.96E-08	1.67E-07	NA	DEg	up
C19orf70	5.46E-09	4.95E-10	0.106742	0.43299	3.07E+10	1.28E+10	1	1	0.106742	0.43299	0.246522	-0.60814	0.106742	0.43299	0.246522	37.21452	5.94E-06	1.32E-08	1.27E-05	3.18E-08	4.14E-08	1.74E-07	NA	DEg	down
MGST1	1.18E-09	1.17E-10	0.339888	0.845361	2.41E+11	3.24E+09	1	1	0.339888	0.845361	0.402062	-0.39571	0.339888	0.845361	0.402062	37.1881	0	2.75E-12	0	8.95E-12	4.20E-08	1.76E-07	NA	DEg	down
MVP	9.98E-09	8.23E-10	0.073034	0.360825	2.03E+09	2.83E+10	1	1	0.073034	0.360825	0.202408	-0.69377	0.073034	0.360825	0.202408	37.16948	0.002387	1.03E-05	0.003728	1.83E-05	4.24E-08	1.77E-07	NA	DEg	down
CHMP5	6.88E-09	6.60E-10	0.098315	0.412371	9.78E+09	1.08E+10	1	1	0.098315	0.412371	0.238413	-0.62267	0.098315	0.412371	0.238413	37.00296	0.000132	1.09E-06	0.000241	2.19E-06	4.59E-08	1.91E-07	NA	DEg	down
PA2G4	1.08E-09	1.46E-10	0.41573	0.958763	9.75E+10	8.12E+09	1	1	0.393258	0.958763	0.410173	-0.38703	0.41573	0.958763	0.433611	36.55004	0.013859	0	0.019363	0	5.73E-08	2.37E-07	NA	DEg	down
MT-ND4L	1.00E-09	0	0.946629	1.721682	4.24E+09	282.1927	1	0.993936	0.803371	1.721649	0.466629	-0.33103	0.946629	1.721649	0.549839	36.42987	2.22E-16	1.23E-09	1.43E-15	3.30E-09	6.07E-08	2.50E-07	NA	DEg	down
FAM162A	7.32E-09	7.59E-10	0.120787	0.453608	5.64E+10	1.8E+09	1	1	0.117978	0.453608	0.260087	-0.58488	0.120787	0.453608	0.266279	35.70095	4.85E-06	1.15E-06	1.04E-05	2.30E-06	8.66E-08	3.55E-07	NA	DEg	down
ZFAND5	7.38E-09	2.76E-10	0.216292	0.628866	2.28E+12	3.14E+10	1	1	0.202247	0.628866	0.321606	-0.49268	0.216292	0.628866	0.34394	35.67714	3.16E-09	5.20E-09	8.66E-09	1.31E-08	8.76E-08	3.58E-07	NA	DEg	down
RBMX	2.41E-09	5.02E-10	0.179775	0.56701	9.61E+13	3.07E+10	1	1	0.179775	0.56701	0.317058	-0.49886	0.179775	0.56701	0.317058	35.63082	5.84E-08	1.60E-07	1.51E-07	3.44E-07	8.96E-08	3.65E-07	NA	DEg	down
B3GNT7	7.27E-10	3.43E-09	0.595506	0.164948	3.06E+10	30780812	1	1	0.505618	0.164948	3.065309	0.486474	0.595506	0.164948	3.610253	35.61943	5.58E-09	0.005853	1.50E-08	0.008348	9.01E-08	3.65E-07	NA	DEg	up
B4GALT1	7.60E-10	3.43E-09	0.595506	0.164948	1.25E+10	30780812	1	1	0.508427	0.164948	3.082338	0.48888	0.595506	0.164948	3.610253	35.61943	2.00E-15	0.003158	1.19E-14	0.004593	9.01E-08	3.65E-07	NA	DEg	up
AES	7.51E-09	7.94E-10	0.081461	0.371134	6.62E+09	2.47E+10	1	1	0.081461	0.371134	0.219491	-0.65858	0.081461	0.371134	0.219491	35.61186	0.000275	4.61E-06	0.000478	8.46E-06	9.05E-08	3.65E-07	NA	DEg	down
POLR2I	1.29E-09	1.73E-10	0.27809	0.731959	2.26E+11	5.77E+09	1	1	0.272472	0.731959	0.37225	-0.42916	0.27809	0.731959	0.379926	35.52019	2.55E-09	3.99E-11	7.08E-09	1.19E-10	9.46E-08	3.80E-07	NA	DEg	down
HSBP1	1.13E-09	1.53E-10	0.303371	0.773196	3.83E+11	4.16E+09	1	1	0.303371	0.773196	0.39236	-0.40632	0.303371	0.773196	0.39236	35.517	0	0	0	0	9.47E-08	3.80E-07	NA	DEg	down
DUOX2	0.448497	1.53E-10	0.743626	0.773196	12572.34	4.16E+09	0.999941	1	0.289326	0.773196	0.374195	-0.4269	0.410112	0.773196	0.530412	35.48381	6.64E-11	0.008035	2.14E-10	0.011244	9.63E-08	3.85E-07	NA	DEg	down
MAOA	5.46E-09	7.41E-10	0.106742	0.42268	3.07E+10	8.68E+09	1	1	0.106742	0.42268	0.252535	-0.59768	0.106742	0.42268	0.252535	35.31924	1.64E-05	0.001206	3.34E-05	0.001822	1.04E-07	4.14E-07	NA	DEg	down
SLC25A24	5.46E-09	7.41E-10	0.106742	0.42268	3.07E+10	8.68E+09	1	1	0.106742	0.42268	0.252535	-0.59768	0.106742	0.42268	0.252535	35.31924	1.64E-05	0.001206	3.34E-05	0.001822	1.04E-07	4.14E-07	NA	DEg	down
SPTAN1	5.46E-09	7.41E-10	0.106742	0.42268	3.07E+10	8.68E+09	1	1	0.106742	0.42268	0.252535	-0.59768	0.106742	0.42268	0.252535	35.31924	1.64E-05	0.001206	3.34E-05	0.001822	1.04E-07	4.14E-07	NA	DEg	down
TXNDC17	1.23E-09	1.14E-10	0.410112	0.938144	6.3E+10	3.5E+09	1	1	0.384831	0.938144	0.410205	-0.387	0.410112	0.938144	0.437153	35.18475	0	0	0	0	1.11E-07	4.40E-07	NA	DEg	down
VDAC2	0.060877	1.90E-10	0.430715	0.927835	15613.91	1.83E+09	0.999972	1	0.337079	0.927835	0.363296	-0.43974	0.404494	0.927835	0.435955	35.14294	1.39E-14	4.44E-16	7.35E-14	2.06E-15	1.14E-07	4.48E-07	NA	DEg	down
CTNNB1	1.22E-09	1.25E-10	0.390449	0.907216	7.77E+10	3.42E+09	1	1	0.379213	0.907216	0.417997	-0.37883	0.390449	0.907216	0.430382	35.10362	3.49E-05	0	6.76E-05	0	1.16E-07	4.55E-07	NA	DEg	down
ANAPC11	1.81E-09	2.25E-10	0.255618	0.690722	4.55E+12	6.89E+09	1	1	0.25	0.690722	0.36194	-0.44136	0.255618	0.690722	0.370074	35.00946	0.000729	3.33E-14	0.001202	1.24E-13	1.21E-07	4.76E-07	NA	DEg	down
UBE2B	7.98E-09	6.98E-10	0.092697	0.391753	4.33E+09	1.64E+10	1	1	0.092697	0.391753	0.23662	-0.62595	0.092697	0.391753	0.23662	34.89401	0.001023	1.46E-07	0.001668	3.16E-07	1.28E-07	5.02E-07	NA	DEg	down
PRR13	2.85E-09	1.35E-10	0.724719	1.391753	1.18E+11	3.07E+09	1	1	0.617978	1.391753	0.444028	-0.35259	0.724719	1.391753	0.520724	34.79165	0	0	0	0	1.35E-07	5.26E-07	NA	DEg	down
TCF7L2	1.16E-08	8.09E-10	0.070225	0.340206	1.24E+09	2.79E+10	1	1	0.070225	0.340206	0.206418	-0.68525	0.070225	0.340206	0.206418	34.4677	0.006849	0.003071	0.010159	0.004473	1.58E-07	6.15E-07	NA	DEg	down
RSRC2	0.09302	1.17E-10	0.393329	0.845361	10392.53	3.24E+09	0.999962	1	0.294944	0.845361	0.348897	-0.4573	0.356742	0.845361	0.421999	34.24607	5.60E-12	3.66E-15	2.07E-11	1.48E-14	1.76E-07	6.83E-07	NA	DEg	down
ARL6IP1	1.35E-09	1.59E-10	0.376404	0.876289	3.52E+11	1.94E+09	1	1	0.353933	0.876289	0.4039	-0.39373	0.376404	0.876289	0.429544	34.03623	0	8.81E-13	0	2.91E-12	1.95E-07	7.54E-07	NA	DEg	down
NDUFA13	7.55E-10	1.03E-10	0.438202	0.969072	6.64E+09	2.87E+09	1	1	0.421348	0.969072	0.434796	-0.36171	0.438202	0.969072	0.452187	33.88602	0	0	0	0	2.09E-07	8.08E-07	NA	DEg	down
TST	7.51E-09	8.23E-10	0.081461	0.360825	6.62E+09	2.83E+10	1	1	0.081461	0.360825	0.225762	-0.64635	0.081461	0.360825	0.225762	33.67794	0.000275	2.97E-06	0.000478	5.63E-06	2.32E-07	8.92E-07	NA	DEg	down
MINOS1	1.92E-09	2.94E-10	0.219101	0.618557	7.37E+12	3.23E+10	1	1	0.219101	0.618557	0.354213	-0.45073	0.219101	0.618557	0.354213	33.60201	6.62E-10	8.54E-08	1.95E-09	1.87E-07	2.40E-07	9.23E-07	NA	DEg	down
COPS9	8.20E-10	1.15E-10	0.412921	0.927835	4.52E+09	3.48E+09	1	1	0.412921	0.927835	0.445037	-0.3516	0.412921	0.927835	0.445037	33.54767	0	0	0	0	2.47E-07	9.45E-07	NA	DEg	down
DDIT4	0.137981	2.33E-10	0.413844	0.835052	17359.3	1.74E+09	0.999976	1	0.292135	0.835052	0.34984	-0.45613	0.356742	0.835052	0.427209	33.41901	1.12E-11	1.37E-14	4.02E-11	5.22E-14	2.63E-07	1.00E-06	NA	DEg	down
SOD2	0.21397	4.95E-10	0.150093	0.43299	18071.75	1.28E+10	0.999992	1	0.109551	0.43299	0.25301	-0.59686	0.117978	0.43299	0.272472	33.40607	0.001612	1.42E-06	0.002563	2.80E-06	2.64E-07	1.01E-06	NA	DEg	down
IGHM	4.23E-09	7.41E-09	0.446629	0.092784	4.52E+11	723921.6	1	1	0.376404	0.092784	4.056804	0.608184	0.446629	0.092784	4.81367	33.21039	0.002023	9.47E-06	0.003187	1.69E-05	2.91E-07	1.10E-06	NA	DEg	up
SFN	0.107009	2.16E-10	0.547334	1.030928	10412.54	2.28E+09	0.999947	1	0.393258	1.030928	0.381461	-0.41855	0.488764	1.030928	0.474101	33.16886	1.11E-16	1.69E-14	7.49E-16	6.42E-14	2.97E-07	1.12E-06	NA	DEg	down
CXCL3	5.93E-09	5.59E-10	0.140449	0.474227	4.09E+11	1.54E+10	1	1	0.13764	0.474227	0.290242	-0.53724	0.140449	0.474227	0.296165	32.98023	2.45E-06	2.52E-07	5.44E-06	5.30E-07	3.25E-07	1.23E-06	NA	DEg	down

PPP1R16A	1.24E-08	8.93E-10	0.064607	0.319588	5.51E+08	1.35E+10	1	1	0.064607	0.319588	0.202157	-0.69431	0.064607	0.319588	0.202157	32.96697	0.000442	0.014043	0.00075	0.019328	3.27E-07	1.23E-06	NA	DEg	down
PRPF4B	0.37537	2.26E-10	0.647575	0.814433	17330.04	2.5E+09	0.999963	1	0.297753	0.814433	0.365595	-0.437	0.404494	0.814433	0.496658	32.72651	2.29E-11	1.01E-08	7.89E-11	2.45E-08	3.68E-07	1.38E-06	NA	DEg	down
TRIR	7.02E-10	1.13E-10	0.398876	0.896907	7.88E+09	4.41E+09	1	1	0.393258	0.896907	0.438461	-0.35807	0.398876	0.896907	0.444724	32.47676	1.08E-08	1.11E-15	2.89E-08	4.77E-15	4.15E-07	1.55E-06	NA	DEg	down
CSKMT	0.176646	2.33E-10	0.443513	0.835052	11478.87	1.74E+09	0.999961	1	0.294944	0.835052	0.353204	-0.45197	0.365169	0.835052	0.437301	32.43905	0.000457	2.18E-13	0.000775	7.55E-13	4.23E-07	1.58E-06	NA	DEg	down
EFNB2	1.06E-08	1.40E-09	0.061798	0.309278	3.06E+08	5.5E+09	1	1	0.061798	0.309278	0.199813	-0.69938	0.061798	0.309278	0.199813	32.22399	0.016316	0.000192	0.02241	0.000313	4.69E-07	1.74E-06	NA	DEg	down
WDR1	1.06E-08	1.40E-09	0.061798	0.309278	3.06E+08	5.5E+09	1	1	0.061798	0.309278	0.199813	-0.69938	0.061798	0.309278	0.199813	32.22399	0.016316	0.000192	0.02241	0.000313	4.69E-07	1.74E-06	NA	DEg	down
SF3B6	6.48E-09	6.63E-10	0.13764	0.463918	2.90E+11	3.72E+09	1	1	0.134831	0.463918	0.290637	-0.53665	0.13764	0.463918	0.296692	32.1868	4.32E-05	5.17E-07	8.29E-05	1.05E-06	4.78E-07	1.77E-06	NA	DEg	down
TSTA3	3.35E-09	1.49E-10	0.33427	0.793814	4.60E+12	4.31E+09	1	1	0.297753	0.793814	0.375091	-0.42586	0.33427	0.793814	0.421093	32.04779	1.02E-14	2.11E-15	5.54E-14	8.86E-15	5.11E-07	1.89E-06	NA	DEg	down
NDUFS4	8.40E-08	2.26E-09	0.022472	0.206186	129229.8	7.19E+08	1	1	0.022472	0.206186	0.108989	-0.96262	0.022472	0.206186	0.108989	31.99953	0.06305	0.325016	0.077084	0.368489	5.23E-07	1.93E-06	NA	DEg	down
PCBP2	1.24E-09	1.25E-10	0.457865	0.979381	2.14E+10	2.12E+09	1	1	0.426966	0.979381	0.435955	-0.36056	0.457865	0.979381	0.467504	31.84964	0	0	0	0	5.63E-07	2.07E-06	NA	DEg	down
AHNAK	0.186918	2.33E-10	0.456026	0.835052	14490.2	1.74E+09	0.999969	1	0.297753	0.835052	0.356568	-0.44786	0.370787	0.835052	0.444028	31.68221	4.23E-09	3.96E-11	1.15E-08	1.18E-10	6.11E-07	2.24E-06	NA	DEg	down
DNAJB6	3.50E-09	7.59E-10	0.134831	0.453608	8.49E+11	1.8E+09	1	1	0.134831	0.453608	0.297242	-0.52689	0.134831	0.453608	0.297242	31.39238	1.41E-06	0.000149	3.19E-06	0.000245	7.03E-07	2.58E-06	NA	DEg	down
NDUFAB1	9.98E-09	8.74E-10	0.073034	0.329897	2.03E+09	1.99E+10	1	1	0.073034	0.329897	0.221383	-0.65485	0.073034	0.329897	0.221383	31.38207	0.003229	0.025156	0.004989	0.033517	7.06E-07	2.58E-06	NA	DEg	down
EIF4E2	5.80E-09	7.41E-10	0.117978	0.42268	9.61E+10	8.68E+09	1	1	0.117978	0.42268	0.279118	-0.55421	0.117978	0.42268	0.279118	31.38124	0.019056	6.14E-08	0.025619	1.36E-07	7.07E-07	2.58E-06	NA	DEg	down
SFT2D1	2.64E-09	3.43E-09	0.011236	0.164948	12287.61	30780812	0.999999	1	0.011236	0.164948	0.068118	-1.16674	0.011236	0.164948	0.068118	31.22939	0.071944	0.476306	0.087116	0.531885	7.61E-07	2.77E-06	NA	DEg	down
RHOC	1.07E-08	1.36E-10	0.426966	0.927835	4.37E+13	2.65E+09	1	1	0.353933	0.927835	0.381461	-0.41855	0.426966	0.927835	0.460175	31.21302	4.21E-11	0	1.37E-10	0	7.67E-07	2.79E-06	NA	DEg	down
SPCS1	1.32E-08	7.52E-10	0.098315	0.381443	1.77E+09	2.02E+10	1	1	0.095506	0.381443	0.25038	-0.6014	0.098315	0.381443	0.257744	31.18175	0.002393	0.010481	0.00373	0.014529	7.78E-07	2.82E-06	NA	DEg	down
S100A16	1.11E-09	0	0.766854	1.412372	9.28E+09	332.98	1	0.995776	0.660112	1.412371	0.467379	-0.33033	0.766854	1.412371	0.542955	31.10571	0	4.73E-08	0	1.06E-07	8.08E-07	2.92E-06	NA	DEg	down
TM9SF3	0.102554	1.59E-10	0.441327	0.876289	10417.95	1.94E+09	0.999958	1	0.320225	0.876289	0.365433	-0.43719	0.396067	0.876289	0.451983	31.06997	3.25E-06	3.33E-16	7.11E-06	1.57E-15	8.22E-07	2.97E-06	NA	DEg	down
PLEC	0.272346	1.49E-10	0.494124	0.793814	10019.5	4.31E+09	0.999951	1	0.283708	0.793814	0.357398	-0.44685	0.359551	0.793814	0.45294	30.84427	1.22E-06	1.70E-05	2.81E-06	2.98E-05	9.17E-07	3.30E-06	NA	DEg	down
SLPI	9.07E-09	2.05E-10	0.325843	0.804124	1.34E+15	2.81E+09	1	1	0.283708	0.804124	0.352816	-0.45245	0.325843	0.804124	0.405215	30.77859	4.27E-12	3.35E-06	1.58E-11	6.30E-06	9.46E-07	3.40E-06	NA	DEg	down
CD151	4.19E-09	1.66E-10	0.382022	0.85567	5.78E+12	1.95E+09	1	1	0.328652	0.85567	0.384087	-0.41557	0.382022	0.85567	0.44646	30.75686	9.44E-15	1.34E-07	5.15E-14	2.91E-07	9.56E-07	3.43E-06	NA	DEg	down
ACTR3	1.16E-08	8.93E-10	0.070225	0.319588	1.24E+09	1.35E+10	1	1	0.070225	0.319588	0.219735	-0.6581	0.070225	0.319588	0.219735	30.6078	0.01577	0.000134	0.02176	0.00022	1.03E-06	3.67E-06	NA	DEg	down
CEACAM1	1.16E-08	8.93E-10	0.070225	0.319588	1.24E+09	1.35E+10	1	1	0.070225	0.319588	0.219735	-0.6581	0.070225	0.319588	0.219735	30.6078	0.01577	0.000134	0.02176	0.00022	1.03E-06	3.67E-06	NA	DEg	down
HIST1H1C	0.436887	8.09E-10	0.144661	0.340206	13523.56	2.79E+10	0.999989	1	0.075843	0.340206	0.222932	-0.65183	0.081461	0.340206	0.239445	30.58382	0.010381	0.000578	0.014751	0.000899	1.04E-06	3.70E-06	NA	DEg	down
FUS	0.593599	1.10E-07	1.175017	0.072165	12572.14	18421.9	0.999907	0.999996	0.280899	0.072165	3.892456	0.590224	0.477528	0.072165	6.617175	30.42532	0.000343	0.003892	0.000587	0.005642	1.12E-06	3.99E-06	NA	DEg	up
EIF5	0.686484	6.60E-10	0.627176	0.412371	10073.95	1.08E+10	0.999938	1	0.146067	0.412371	0.354213	-0.45073	0.196629	0.412371	0.476826	30.05796	0.00011	0.00544	0.000202	0.007784	1.34E-06	4.76E-06	NA	DEg	down
MRPL57	1.27E-09	2.39E-10	0.266854	0.670103	1.33E+11	9.14E+09	1	1	0.264045	0.670103	0.394036	-0.40446	0.266854	0.670103	0.398228	29.98802	2.27E-05	3.44E-15	4.53E-05	1.41E-14	1.39E-06	4.91E-06	NA	DEg	down
BLVRB	3.93E-09	2.25E-10	0.27809	0.690722	1.67E+14	6.89E+09	1	1	0.255618	0.690722	0.370074	-0.43171	0.27809	0.690722	0.402608	29.98129	4.64E-11	1.53E-12	1.50E-10	5.00E-12	1.39E-06	4.92E-06	NA	DEg	down
ATP5F1A	4.18E-09	7.41E-10	0.123596	0.42268	2.55E+11	8.68E+09	1	1	0.123596	0.42268	0.292409	-0.53401	0.123596	0.42268	0.292409	29.72418	0.000101	4.92E-08	0.000188	1.09E-07	1.58E-06	5.54E-06	NA	DEg	down
TMED9	1.24E-08	1.64E-09	0.064607	0.298969	5.51E+08	3.02E+09	1	1	0.064607	0.298969	0.216098	-0.66535	0.064607	0.298969	0.216098	29.08173	0.001558	0.000254	0.002485	0.000412	2.15E-06	7.49E-06	NA	DEg	down
SELENOH	7.87E-10	1.26E-10	0.455056	0.948454	5.73E+09	3.3E+09	1	1	0.432584	0.948454	0.456094	-0.34095	0.455056	0.948454	0.479787	29.07329	0.03951	0	0.050229	0	2.16E-06	7.50E-06	NA	DEg	down
SNX9	0.908207	2.36E-09	0.734429	0.216495	14854.69	7.89E+08	0.999951	1	0.047753	0.216495	0.220572	-0.65645	0.067416	0.216495	0.311396	29.02668	0.00758	0.023429	0.011077	0.031355	2.21E-06	7.65E-06	NA	DEg	down
SPINT2	6.97E-09	1.05E-10	0.542135	1.072165	1.18E+13	2.37E+09	1	1	0.435393	1.072165	0.406088	-0.39138	0.542135	1.072165	0.505645	28.95117	0	0	0	0	2.29E-06	7.92E-06	NA	DEg	down
CD2AP	0.567174	5.65E-10	0.992952	0.484536	15104	1.86E+10	0.999934	1	0.272472	0.484536	0.562336	-0.25	0.429775	0.484536	0.886983	28.94655	5.95E-08	7.22E-08	1.53E-07	1.59E-07	2.30E-06	7.92E-06	NA	DEg	down
KRT7	0.661726	7.04E-08	1.195759	0.041237	12541.78	14475.98	0.999905	0.999997	0.25	0.041237	6.0625	0.782652	0.404494	0.041237	9.808989	28.67707	0.025452	0.024386	0.033609	0.032588	2.62E-06	8.95E-06	NA	DEg	up
YWHAG	2.64E-09	4.33E-09	0.011236	0.154639	12287.61	14066667	0.999999	1	0.011236	0.154639	0.072659	-1.13871	0.011236	0.154639	0.072659	28.6063	0.134503	0.492036	0.15648	0.548762	2.71E-06	9.24E-06	NA	DEg	down
SQLE	0.789658	2.39E-09	0.240379	0.247423	14335.4	9E+08	0.999983	1	0.044944	0.247423	0.181648	-0.74077	0.050562	0.247423	0.204354	28.55373	0.050255	0.401711	0.062555	0.451988	2.78E-06	9.46E-06	NA	DEg	down
FCGBP	0.073574	0.270112	0.924782	0.480232	14794.38	16773.71	0.999937	0.999971	0.755618	0.350515	2.155734	0.333595	0.856742	0.350515	2.444233	28.51695	9.30E-06	1.97E-06	1.94E-05	3.81E-06	2.83E-06	9.61E-06	NA	DEg	up
ELOC	1.06E-08	1.59E-09	0.061798	0.28866	3.06E+08	1.87E+09	1	1	0.061798	0.28866	0.214085	-0.66941	0.061798	0.28866	0.214085	28.31705	0.017132	0.045686	0.023315	0.057674	3.12E-06	1.05E-05	NA	DEg	down
BPIFB1	8.30E-09	1.13E-10	0.446629	0.927835	9.22E+12	3.78E+09	1	1	0.373596	0.927835	0.402653	-0.39507	0.446629	0.927835	0.481367	28.25621	0	0	0	0	3.21E-06	1.08E-05	NA	DEg	down
EIF4G2	1.59E-09	1.46E-10	0.469101	0.958763	8.92E+12	8.12E+09	1	1	0.401685	0.958763	0.418962	-0.37783	0.469101	0.958763	0.489278	28.0681	0	1.36E-10	0	3.85E-10	3.51E-06	1.18E-05	NA	DEg	down
MIDN	1.29E-09	1.46E-10	0.469101	0.958763	2.54E+10	8.12E+09	1	1	0.410112	0.958763	0.427752	-0.36881	0.469101	0.958763	0.489278	28.05965	0	0	0	0	3.53E-06	1.18E-05	NA	DEg	down
ATOX1	3.66E-09	2.04E-10	0.303371	0.71134	3.39E+12	5.78E+09	1	1	0.27809	0.71134	0.390938	-0.40789	0.303371	0.71134	0.426478	28.02422	5.59E-13	6.25E-11	2.38E-12	1.82E-10	3.59E-06	1.20E-05	NA	DEg	down
FKBP2	0.055817	1.39E-10	0.446257	0.886598	18202.51	2.4E+09	0.999975	1	0.339888	0.886598	0.383362	-0.41639	0.421348	0.886598	0.475242	27.90561	1.11E-16	8.88E-16	7.49E-16	3.91E-15	3.80E-06	1.26E-05	NA	DEg	down
CDKN1A	0.15667	2.40E-10	0.456324	0.824742	17694.03	2.3E+09	0.999974	1	0.308989	0.824742	0.374649	-0.42638	0.384831	0.824742	0.466608	27.90268	4.04E-06	0.000276	8.74E-06	0.000444	3.81E-06	1.26E-05	NA	DEg	down
SYPL1	3.06E-08	1.74E-09	0.036517	0.226804	4283965	1.21E+09	1	1	0.036517	0.226804	0.161006	-0.79316	0.036517	0.226804	0.161006	27.89713	0.027779	0.021	0.036411	0.028383	3.82E-06	1.26E-05	NA	DEg	down
MYO6	0.519437	5.81E-10	0.981993	0.525773	17908.86	2.87E+10	0.999945	1	0.300562	0.525773	0.571657	-0.24286	0.47191	0.525773	0.897555	27.76601	2.12E-10	3.51E-07	6.48E-10	7.26E-07	4.07E-06	1.34E-05	NA	DEg	down
ACTN4	0.057878	1.13E-10	0.471086	0.917526	19896.23	3.74E+09	0.999976	1	0.353933	0.917526	0.385747	-0.4137	0.44382	0.917526	0.483714	27.74333	0	1.11E-16	0	5.49E-16	4.11E-06	1.35E-05	NA	DEg	down
MZT2A	8.78E-10	1.16E-09	0.696629	0.268041	6.21E+10	1.5E+09	1	1	0.578652	0.268041	2.158818	0.334216	0.696629	0.268041	2.598963	27.74572	5.44E-15	1.21E-10	3.06E-14	3.47E-10	4.11E-06	1.35E-05	NA	DEg	up
FOSL2	1.81E-08	2.00E-09	0.058989	0.278351	92103425	9.49E+08	1	1	0.058989	0.278351	0.211923	-0.67382	0.058989	0.278351	0.211923	27.55473	0.005209	0.048005	0.007805	0.060176	4.50E-06	1.47E-05	NA	DEg	down
HILPDA	1.81E-08	2.00E-09	0.058989	0.278351	92103425	9.49E+08	1	1	0.058989	0.278351	0.211923	-0.67382	0.058989	0.278351	0.211923	27.55473	0.005209	0.048005	0.007805	0.060176	4.50E-06	1.47E-05	NA	DEg	down
ZFP36L1	0.116239	2.33E-10	0.444983	0.835052	10070.95	1.74E+09	0.999956	1	0.317416	0.835052	0.380115	-0.42008	0.393258	0.835052	0.470939	27.23027	3.36E-05	0.000288	6.53E-05	0.000461	5.27E-06	1.71E-05	NA	DEg	down
SYF2	0.07683	2.33E-10	0.425987	0.835052	10939.23	1.74E+09	0.999961	1	0.320225	0.835052	0.383479	-0.41626	0.393258	0.835052	0.470939	26.92389	5.33E-14	3.11E-15	2.66E-13	1.27E-14	6.11E-06	1.98E-05	NA	DEg	down
LAMB3	0.22849	1.17E-10	0.542493	0.845361	12472.37	3.24E+09	0.999957	1	0.323034	0.845361	0.382125	-0.41779	0.418539	0.845361	0.495101	26.86171	1.87E-14	1.24E-13	9.65E-14	4.37E-13	6.29E-06	2.04E-05	NA	DEg	down
EPN1	9.00E-07	3.19E-09	0.019663	0.175258	15371.94	49600291	0.999999	1	0.019663	0.175258	0.112194	-0.95003	0.019663	0.175258	0.112194	26.79902	0.122853	0.424385	0.143867	0.476897	6.49E-06	2.10E-05	NA	DEg	down
S100A14	0.059633	2.54E-10	1.350181	1.979381	18888.49	1.31E+09	0.999929	1	1.005618	1.979381	0.508047	-0.2941	1.269663	1.979381	0.641444	26.60621	0	0	0	0	7.12E-06	2.29E-05	NA	DEg	down
KHDRBS1	1.88E-08	2.39E-09	0.047753	0.247423	27413284	9E+08	1	1	0.047753	0.247423	0.193001	-0.71444	0.047753	0.247423	0.193001	26.5323	0.352938	0.147293	0.383534	0.173631	7.38E-06	2.37E-05	NA	DEg	down
HK2	1.06E-08	2.00E-09	0.061798	0.278351	3.06E+08	9.49E+08	1	1	0.061798	0.278351	0.222014	-0.65362	0.061798	0.278351	0.222014	26.40815	0.007566	0.000554	0.011075	0.000863	7.83E-06	2.50E-05	NA	DEg	down
SNRNP27	1.06E-08	2.00E-09	0.061798	0.278351	3.06E+08	9.49E+08	1	1	0.061798	0.278351	0.222014	-0.65362	0.061798	0.278351	0.222014	26.40815	0.007566	0.000554	0.011075	0.000863	7.83E-06	2.50E-05	NA	DEg	down
LGALS3BP	7.97E-09	1.26E-10	0.474719	0.948454	1.83E+13	3.3E+09	1	1	0.384831	0.948454	0.405746	-0.39175	0.474719	0.948454	0.500519	26.19182	0	0	0	0	8.69E-06	2.77E-05	NA	DEg	down
ZBTB7A	3.95E-08	4.33E-09	0.014045	0.154639	16271.31	14066667	0.999999	1	0.014045	0.154639	0.090824	-1.0418	0.014045	0.154639	0.090824	26.15164	0.320117	0.586326	0.350006	0.643441	8.86E-06	2.82E-05	NA	DEg	down
TRMT112	1.00E-08	1.59E-09	0.067416	0.28866	9.86E+08	1.87E+09	1	1	0.067416	0.28866	0.233547	-0.63163	0.067416	0.28866	0.233547	26.09544	0.013321	0.005714	0.018641	0.008163	9.11E-06	2.89E-05	NA	DEg	down
HSPA5	9.98E-09	1.64E-09	0.073034	0.298969	2.03E+09	3.02E+09	1	1	0.073034	0.298969	0.244285	-0.6121	0.073034	0.298969	0.244285	25.83808	0.009446	0.0002	0.013559	0.000326	1.03E-05	3.26E-05	NA	DEg	down
SARAF	2.16E-09	2.12E-10	0.337079	0.742268	7.10E+11	3.92E+09	1	1	0.308989	0.742268	0.416277	-0.38062	0.337079	0.742268	0.45412	25.7199	1.15E-13	1.08E-14	5.41E-13	4.17E-14	1.09E-05	3.45E-05	NA	DEg	down
GTF2A2	9.83E-09	1.40E-09	0.078652	0.309278	3.72E+09	5.5E+09	1	1	0.078652	0.309278	0.254307	-0.59464	0.078652	0.309278	0.254307	25.62836	0.001925	0.035352	0.003037	0.045533	1.14E-05	3.60E-05	NA	DEg	down
SELENOK	0.079177	1.36E-10	0.460629	0.865979	12242.94	2.27E+09	0.999962	1	0.339888	0.865979	0.392489	-0.40617	0.424157	0.865979	0.489801	25.54339	0.000293	6.33E-15	0.000507	2.50E-14	1.19E-05	3.74E-05	NA	DEg	down
ATP5IF1	7.44E-09	9.68E-11	0.561798	1.061856	1.44E+13	1.74E+09	1	1	0.457865	1.061856	0.431193	-0.36533	0.561798	1.061856	0.529072	25.37812	0	0	0	0	1.29E-05	4.04E-05	NA	DEg	down
LYZ	9.79E-10	0	2.157303	1.381444	2.27E+09	412.3717	1	0.996661	1.910112	1.381443	1.382693	0.140726	2.157303	1.381443	1.56163	24.9382	0	0	0	0	1.59E-05	4.98E-05	NA	DEg	up
ACTR2	1.06E-08	1.16E-09	0.061798	0.268041	3.06E+08	1.5E+09	1	1	0.061798	0.268041	0.230553	-0.63723	0.061798	0.268041	0.230553	24.53605	0.007489	0.021409	0.011017	0.028826	1.93E-05	6.02E-05	NA	DEg	down
RALA	1.06E-08	1.16E-09	0.061798	0.268041	3.06E+08	1.5E+09	1	1	0.061798	0.268041	0.230553	-0.63723	0.061798	0.268041	0.230553	24.53605	0.007489	0.021409	0.011017	0.028826	1.93E-05	6.02E-05	NA	DEg	down
ABCC3	0.520567	5.65E-10	0.955014	0.484536	13688.87	1.86E+10	0.99993	1	0.294944	0.484536	0.608714	-0.21559	0.457865	0.484536	0.944956	24.49814	3.96E-07	1.12E-07	9.50E-07	2.45E-07	1.97E-05	6.12E-05	NA	DEg	down
COMTD1	0.508354	5.59E-10	0.428507	0.474227	13895.93	1.54E+10	0.999969	1	0.171348	0.474227	0.361321	-0.44211	0.210674	0.474227	0.444248	24.49246	0.000335	2.41E-05	0.000575	4.17E-05	1.97E-05	6.12E-05	NA	DEg	down
UBE2I	9.00E-07	3.43E-09	0.019663	0.164948	15371.94	30780812	0.999999	1	0.019663	0.164948	0.119206	-0.9237	0.019663	0.164948	0.119206	24.42409	0.108773	0.108295	0.128563	0.130423	2.04E-05	6.30E-05	NA	DEg	down
NDUFA1	9.34E-10	1.19E-10	0.646067	1.164948	1.05E+10	6.82E+08	1	1	0.598315	1.164948	0.513597	-0.28938	0.646067	1.164948	0.554589	24.37203	0	1.51E-11	0	4.61E-11	2.09E-05	6.45E-05	NA	DEg	down
SRP9	5.52E-09	8.54E-10	0.103933	0.350515	2.29E+10	2.9E+10	1	1	0.103933	0.350515	0.296514	-0.52796	0.103933	0.350515	0.296514	24.26659	0.000351	0.001341	0.000599	0.002002	2.20E-05	6.76E-05	NA	DEg	down
TRIM15	1.00E-08	2.00E-09	0.067416	0.278351	9.86E+08	9.49E+08	1	1	0.067416	0.278351	0.242197	-0.61583	0.067416	0.278351	0.242197	24.26577	0.018821	0.00061	0.025341	0.000945	2.20E-05	6.76E-05	NA	DEg	down
SLIRP	6.16E-10	1.03E-10	0.505618	0.969072	8.18E+09	2.87E+09	1	1	0.491573	0.969072	0.507262	-0.29477	0.505618	0.969072	0.521755	24.09783	0	1.34E-13	0	4.70E-13	2.38E-05	7.32E-05	NA	DEg	down
NDUFB8	2.78E-09	5.65E-10	0.213483	0.536082	7.64E+12	3.05E+10	1	1	0.213483	0.536082	0.398228	-0.39987	0.213483	0.536082	0.398228	24.01834	3.04E-09	1.87E-09	8.36E-09	4.93E-09	2.48E-05	7.57E-05	NA	DEg	down
CCDC107	4.37E-07	4.33E-09	0.016854	0.154639	12677.25	14066667	0.999999	1	0.016854	0.154639	0.108989	-0.96262	0.016854	0.154639	0.108989	23.99964	0.132975	0.531651	0.154905	0.588027	2.50E-05	7.63E-05	NA	DEg	down
SLC38A2	0.255974	2.33E-10	0.59651	0.835052	15626.52	1.74E+09	0.999962	1	0.33427	0.835052	0.400298	-0.39762	0.44382	0.835052	0.531488	23.98203	7.33E-14	4.79E-13	3.53E-13	1.63E-12	2.52E-05	7.68E-05	NA	DEg	down
SOD1	1.14E-09	1.24E-10	0.853933	1.43299	2.6E+10	2.38E+09	1	1	0.738764	1.43299	0.51554	-0.28774	0.853933	1.43299	0.59591	23.78572	0	0	0	0	2.77E-05	8.39E-05	NA	DEg	down
NAA38	4.98E-09	6.60E-10	0.140449	0.412371	7.98E+11	1.08E+10	1	1	0.140449	0.412371	0.34059	-0.46777	0.140449	0.412371	0.34059	23.74786	5.10E-06	4.10E-07	1.09E-05	8.41E-07	2.82E-05	8.53E-05	NA	DEg	down
MRPL14	3.04E-12	5.93E-09	0.005618	0.113402	17050.59	1203645	1	1	0.005618	0.113402	0.04954	-1.30504	0.005618	0.113402	0.04954	23.70743	0.331246	0.74919	0.361729	0.804318	2.88E-05	8.66E-05	NA	DEg	down
NME2	8.72E-09	8.93E-10	0.089888	0.319588	2.82E+09	1.35E+10	1	1	0.089888	0.319588	0.281261	-0.55089	0.089888	0.319588	0.281261	23.67218	0.001019	0.014051	0.001665	0.019328	2.92E-05	8.76E-05	NA	DEg	down
ARL5B	3.95E-08	4.80E-09	0.014045	0.14433	16271.31	7298606	0.999999	1	0.014045	0.14433	0.097311	-1.01184	0.014045	0.14433	0.097311	23.66198	0.302382	0.532528	0.331021	0.588027	2.94E-05	8.76E-05	NA	DEg	down
SEC11C	3.95E-08	4.80E-09	0.014045	0.14433	16271.31	7298606	0.999999	1	0.014045	0.14433	0.097311	-1.01184	0.014045	0.14433	0.097311	23.66198	0.302382	0.532528	0.331021	0.588027	2.94E-05	8.76E-05	NA	DEg	down
SLC20A1	3.95E-08	4.80E-09	0.014045	0.14433	16271.31	7298606	0.999999	1	0.014045	0.14433	0.097311	-1.01184	0.014045	0.14433	0.097311	23.66198	0.302382	0.532528	0.331021	0.588027	2.94E-05	8.76E-05	NA	DEg	down
AKR7A3	3.06E-08	2.26E-09	0.036517	0.206186	4283965	7.19E+08	1	1	0.036517	0.206186	0.177107	-0.75176	0.036517	0.206186	0.177107	23.66072	0.031759	0.026382	0.041128	0.034889	2.94E-05	8.76E-05	NA	DEg	down
SH3KBP1	3.06E-08	2.26E-09	0.036517	0.206186	4283965	7.19E+08	1	1	0.036517	0.206186	0.177107	-0.75176	0.036517	0.206186	0.177107	23.66072	0.031759	0.026382	0.041128	0.034889	2.94E-05	8.76E-05	NA	DEg	down
AKAP9	0.153179	1.17E-10	0.507513	0.845361	10000.07	3.24E+09	0.999949	1	0.337079	0.845361	0.398739	-0.39931	0.429775	0.845361	0.508393	23.65499	9.33E-15	7.74E-14	5.12E-14	2.74E-13	2.95E-05	8.77E-05	NA	DEg	down
COPE	0.315739	1.53E-10	0.615771	0.773196	15931.08	4.16E+09	0.999961	1	0.314607	0.773196	0.406891	-0.39052	0.421348	0.773196	0.544944	23.59111	3.38E-13	7.81E-12	1.51E-12	2.43E-11	3.04E-05	9.03E-05	NA	DEg	down
NDUFB2	7.07E-10	1.25E-10	0.525281	0.989691	1.52E+10	9.35E+09	1	1	0.5	0.989691	0.505208	-0.29653	0.525281	0.989691	0.530753	23.4929	0	0	0	0	3.19E-05	9.43E-05	NA	DEg	down
GALNT1	2.64E-09	3.38E-09	0.011236	0.134021	12287.61	5280704	0.999999	1	0.011236	0.134021	0.083838	-1.07656	0.011236	0.134021	0.083838	23.44818	0.27269	0.547971	0.304127	0.60433	3.26E-05	9.62E-05	NA	DEg	down
RAB25	1.16E-08	2.00E-09	0.070225	0.278351	1.24E+09	9.49E+08	1	1	0.070225	0.278351	0.252289	-0.5981	0.070225	0.278351	0.252289	23.26394	0.001241	0.021554	0.002009	0.028978	3.56E-05	0.000105	NA	DEg	down
SERPINB5	2.58E-08	2.36E-09	0.042135	0.216495	10877582	7.89E+08	1	1	0.042135	0.216495	0.194623	-0.71081	0.042135	0.216495	0.194623	23.05649	0.418817	0.190316	0.44587	0.222285	3.93E-05	0.000115	NA	DEg	down
BSG	6.82E-10	0	0.536517	1.000015	3.86E+09	419.4838	1	0.997622	0.502809	1	0.502809	-0.2986	0.536517	1	0.536517	22.80252	0.000117	0	0.000214	0	4.44E-05	0.00013	NA	DEg	down
YME1L1	0.694596	1.74E-09	1.296866	0.226804	18234.9	1.21E+09	0.999929	1	0.22191	0.226804	0.978422	-0.00947	0.396067	0.226804	1.746297	22.64903	3.18E-05	3.85E-06	6.22E-05	7.18E-06	4.78E-05	0.000139	NA	DEg	up
SOX4	0.448356	8.23E-10	1.084602	0.360825	11186.25	2.83E+10	0.999903	1	0.365169	0.360825	1.012039	0.005197	0.598315	0.360825	1.658186	22.36624	2.93E-10	2.73E-06	8.84E-10	5.21E-06	5.47E-05	0.000159	NA	DEg	up
WSB1	4.33E-08	2.94E-10	0.27809	0.618557	5.45E+13	3.23E+10	1	1	0.244382	0.618557	0.395084	-0.40331	0.27809	0.618557	0.449579	22.01004	8.05E-05	0.000281	0.000151	0.000451	6.49E-05	0.000187	NA	DEg	down
ANKRD11	0.593599	3.19E-09	1.175017	0.175258	12572.14	49600291	0.999907	1	0.280899	0.175258	1.602776	0.204873	0.477528	0.175258	2.724719	21.87852	0.000148	0.000109	0.000268	0.000181	6.91E-05	0.000198	NA	DEg	up
NEDD8	4.39E-10	1.46E-10	0.516854	0.958763	2.47E+09	8.12E+09	1	1	0.516854	0.958763	0.539084	-0.26834	0.516854	0.958763	0.539084	21.78759	1.73E-07	0	4.24E-07	0	7.22E-05	0.000206	NA	DEg	down
SDCBP	1.48E-09	1.25E-10	0.502809	0.938144	2.43E+10	3.3E+09	1	1	0.429775	0.938144	0.458112	-0.33903	0.502809	0.938144	0.535961	21.67243	0	0	0	0	7.63E-05	0.000218	NA	DEg	down
RHEB	4.37E-07	4.80E-09	0.016854	0.14433	12677.25	7298606	0.999999	1	0.016854	0.14433	0.116774	-0.93266	0.016854	0.14433	0.116774	21.61003	0.227474	0.160859	0.255944	0.189121	7.86E-05	0.000224	NA	DEg	down
SYNGR2	4.37E-07	4.80E-09	0.016854	0.14433	12677.25	7298606	0.999999	1	0.016854	0.14433	0.116774	-0.93266	0.016854	0.14433	0.116774	21.61003	0.227474	0.160859	0.255944	0.189121	7.86E-05	0.000224	NA	DEg	down
RBX1	1.16E-08	1.16E-09	0.070225	0.268041	1.24E+09	1.5E+09	1	1	0.070225	0.268041	0.261992	-0.58171	0.070225	0.268041	0.261992	21.50836	0.001339	0.000831	0.002155	0.001275	8.25E-05	0.000234	NA	DEg	down
ISG20	7.33E-09	2.00E-09	0.075843	0.278351	3.99E+09	9.49E+08	1	1	0.075843	0.278351	0.272472	-0.56468	0.075843	0.278351	0.272472	21.37567	0.004241	0.010195	0.006419	0.014155	8.80E-05	0.000249	NA	DEg	down
TUBA1C	4.28E-09	1.25E-10	0.505618	0.938144	5.42E+12	3.3E+09	1	1	0.407303	0.938144	0.434159	-0.36235	0.505618	0.938144	0.538955	21.31099	0	0	0	0	9.07E-05	0.000257	NA	DEg	down
RNF213	0.314386	1.73E-10	1.089813	0.731959	19030.7	5.77E+09	0.999943	1	0.5	0.731959	0.683099	-0.16552	0.747191	0.731959	1.02081	21.28583	1.09E-14	1.80E-09	5.87E-14	4.75E-09	9.18E-05	0.000259	NA	DEg	up
ALDH1A1	1.26E-09	3.19E-09	0.485955	0.175258	2.3E+10	49600291	1	1	0.44382	0.175258	2.532386	0.40353	0.485955	0.175258	2.772802	21.27191	6.94E-11	1.60E-11	2.23E-10	4.86E-11	9.24E-05	0.00026	NA	DEg	up
TPMT	3.95E-08	3.38E-09	0.014045	0.134021	16271.31	5280704	0.999999	1	0.014045	0.134021	0.104797	-0.97965	0.014045	0.134021	0.104797	21.21	0.253387	0.210637	0.284382	0.244416	9.52E-05	0.000268	NA	DEg	down
JTB	1.65E-09	2.39E-10	0.317416	0.670103	2.29E+11	9.14E+09	1	1	0.300562	0.670103	0.448531	-0.34821	0.317416	0.670103	0.473682	21.1567	7.17E-14	1.07E-12	3.47E-13	3.48E-12	9.77E-05	0.000274	NA	DEg	down
NT5C	0.761788	1.16E-09	0.919775	0.268041	13910.5	1.5E+09	0.999934	1	0.143258	0.268041	0.534464	-0.27208	0.219101	0.268041	0.817416	21.02482	3.81E-05	0.000625	7.34E-05	0.000967	0.000104	0.000291	NA	DEg	down
N4BP2L2	0.61851	4.33E-09	1.178111	0.154639	12275.64	14066667	0.999904	1	0.264045	0.154639	1.707491	0.232358	0.449438	0.154639	2.906367	20.98677	0.008102	0.000187	0.011783	0.000307	0.000106	0.000296	NA	DEg	up
CAST	0.248676	1.86E-10	1.009454	1.072165	17268.48	3.39E+09	0.999942	1	0.533708	1.072165	0.497785	-0.30296	0.758427	1.072165	0.707379	20.9401	0	1.22E-15	0	5.23E-15	0.000108	0.000302	NA	DEg	down
TMED2	4.14E-10	1.25E-10	0.539326	0.979381	9.31E+09	2.12E+09	1	1	0.522472	0.979381	0.533471	-0.27289	0.539326	0.979381	0.55068	20.92551	7.06E-10	1.33E-10	2.07E-09	3.81E-10	0.000109	0.000303	NA	DEg	down
SDF2L1	3.17E-11	5.93E-09	0.008427	0.113402	14060.96	1203645	0.999999	1	0.008427	0.113402	0.074311	-1.12895	0.008427	0.113402	0.074311	20.80352	0.281029	0.582108	0.311865	0.6396	0.000116	0.000321	NA	DEg	down
UPF2	0.503253	6.60E-10	0.35625	0.412371	12937.82	1.08E+10	0.999972	1	0.148876	0.412371	0.361025	-0.44246	0.176966	0.412371	0.429143	20.7391	0.000267	0.001305	0.000466	0.001955	0.000119	0.000331	NA	DEg	down
SAP18	1.29E-09	1.03E-10	0.533708	0.969072	3.32E+10	2.87E+09	1	1	0.469101	0.969072	0.484072	-0.31509	0.533708	0.969072	0.550741	20.69878	0	7.55E-15	0	2.96E-14	0.000122	0.000336	NA	DEg	down
NMT1	9.98E-09	1.16E-09	0.073034	0.268041	2.03E+09	1.5E+09	1	1	0.073034	0.268041	0.272472	-0.56468	0.073034	0.268041	0.272472	20.58656	0.001327	0.000586	0.00214	0.000909	0.000128	0.000354	NA	DEg	down
ZC3H15	8.72E-09	1.64E-09	0.089888	0.298969	2.82E+09	3.02E+09	1	1	0.089888	0.298969	0.300659	-0.52193	0.089888	0.298969	0.300659	20.38177	0.030108	0.002528	0.039348	0.003713	0.000141	0.000389	NA	DEg	down
PSAP	0.062544	1.13E-10	0.518377	0.896907	18094.44	4.41E+09	0.999971	1	0.379213	0.896907	0.422801	-0.37386	0.485955	0.896907	0.541812	20.30516	1.57E-13	1.64E-13	7.19E-13	5.73E-13	0.000147	0.000402	NA	DEg	down
DDT	4.68E-10	1.25E-10	0.544944	0.979381	1.84E+09	2.12E+09	1	1	0.530899	0.979381	0.542076	-0.26594	0.544944	0.979381	0.556416	20.28843	2.59E-06	2.13E-11	5.70E-06	6.41E-11	0.000148	0.000404	NA	DEg	down
NDUFA11	4.68E-10	1.25E-10	0.544944	0.979381	1.84E+09	2.12E+09	1	1	0.530899	0.979381	0.542076	-0.26594	0.544944	0.979381	0.556416	20.28843	2.59E-06	2.13E-11	5.70E-06	6.41E-11	0.000148	0.000404	NA	DEg	down
SERPINB1	6.20E-10	1.11E-10	0.615169	1.072165	2.22E+10	8.68E+09	1	1	0.561798	1.072165	0.523984	-0.28068	0.615169	1.072165	0.573763	20.19013	0	0	0	0	0.000155	0.000422	NA	DEg	down
GDI2	1.81E-08	1.41E-09	0.058989	0.237113	92103425	1.72E+09	1	1	0.058989	0.237113	0.248779	-0.60419	0.058989	0.237113	0.248779	20.10898	0.008301	0.032466	0.012052	0.042182	0.000161	0.000438	NA	DEg	down
CYB5A	1.24E-08	2.39E-09	0.064607	0.247423	5.51E+08	9E+08	1	1	0.064607	0.247423	0.261119	-0.58316	0.064607	0.247423	0.261119	19.92561	0.009384	0.02817	0.013492	0.037198	0.000176	0.000478	NA	DEg	down
PSMD8	9.00E-07	4.80E-09	0.019663	0.14433	15371.94	7298606	0.999999	1	0.019663	0.14433	0.136236	-0.86571	0.019663	0.14433	0.136236	19.79293	0.370298	0.1284	0.399957	0.152572	0.000187	0.000508	NA	DEg	down
SFPQ	3.10E-09	8.46E-10	0.86236	0.443299	2.44E+11	7.39E+09	1	1	0.601124	0.443299	1.356023	0.132267	0.86236	0.443299	1.945323	19.68622	0	4.66E-15	0	1.86E-14	0.000197	0.000534	NA	DEg	up

RGS2	0.867073	2.78E-09	0.845275	0.195876	12001.96	4.98E+08	0.99993	1	0.075843	0.195876	0.387197	-0.41207	0.11236	0.195876	0.573625	19.63456	0.006281	0.007611	0.009379	0.010684	0.000202	0.000546	NA	DEg	down
PLA2G16	0.76461	2.26E-09	0.226733	0.206186	12542.11	7.19E+08	0.999982	1	0.047753	0.206186	0.231601	-0.63526	0.053371	0.206186	0.258848	19.5784	0.06536	0.191505	0.079795	0.223381	0.000208	0.00056	NA	DEg	down
HSPD1	7.58E-10	1.46E-10	0.536517	0.958763	6.64E+09	8.12E+09	1	1	0.491573	0.958763	0.512716	-0.29012	0.536517	0.958763	0.559593	19.52151	0	0	0	0	0.000213	0.000574	NA	DEg	down
KLF3	0.307489	2.26E-10	0.766629	0.814433	10846	2.5E+09	0.999929	1	0.370787	0.814433	0.45527	-0.34173	0.530899	0.814433	0.651863	19.51605	3.16E-13	5.62E-12	1.42E-12	1.80E-11	0.000214	0.000575	NA	DEg	down
COL16A1	0.267221	1.15E-08	0.440834	0.082474	12280	445025.3	0.999964	1	0.261236	0.082474	3.167486	0.500715	0.323034	0.082474	3.916784	19.40048	0.110267	0.096332	0.130156	0.116647	0.000226	0.000605	NA	DEg	up
HNRNPAB	4.18E-09	8.54E-10	0.123596	0.350515	2.55E+11	2.9E+10	1	1	0.123596	0.350515	0.352611	-0.4527	0.123596	0.350515	0.352611	19.3648	9.62E-06	0.00039	2.00E-05	0.000614	0.00023	0.000615	NA	DEg	down
LGALS1	7.63E-10	1.25E-10	0.561798	0.989691	6.74E+09	9.35E+09	1	1	0.505618	0.989691	0.510885	-0.29168	0.561798	0.989691	0.56765	19.28048	0	0	0	0	0.000239	0.000638	NA	DEg	down
KDELR2	4.31E-10	1.25E-10	0.561798	0.989691	8.88E+08	9.35E+09	1	1	0.55618	0.989691	0.561973	-0.25028	0.561798	0.989691	0.56765	19.28011	0	5.43E-14	0	1.99E-13	0.000239	0.000638	NA	DEg	down
NR4A1	0.227416	1.17E-10	0.639907	0.845361	13370.63	3.24E+09	0.999952	1	0.365169	0.845361	0.431968	-0.36455	0.494382	0.845361	0.584818	18.93042	3.28E-05	6.98E-12	6.41E-05	2.19E-11	0.000283	0.000752	NA	DEg	down
ALDOA	1.88E-08	2.26E-09	0.047753	0.206186	27413284	7.19E+08	1	1	0.047753	0.206186	0.231601	-0.63526	0.047753	0.206186	0.231601	18.79055	0.030244	0.01757	0.039425	0.023838	0.000302	0.000803	NA	DEg	down
LINC0113:	0.539742	8.54E-10	0.958183	0.350515	17473.65	2.9E+10	0.999945	1	0.283708	0.350515	0.809402	-0.09184	0.441011	0.350515	1.258179	18.49647	4.17E-07	0.00013	9.97E-07	0.000215	0.000347	0.000922	NA	DEg	up
SLC25A37	0.727914	1.16E-09	0.887857	0.268041	10988.26	1.5E+09	0.999919	1	0.160112	0.268041	0.597342	-0.22378	0.241573	0.268041	0.901253	18.43354	0.000211	0.001233	0.000373	0.001859	0.000358	0.000948	NA	DEg	down
SRSF11	0.641576	8.74E-10	0.830728	0.329897	10625.72	1.99E+10	0.999922	1	0.202247	0.329897	0.613062	-0.2125	0.297753	0.329897	0.902563	18.36018	0.000148	0.001103	0.000268	0.00168	0.000371	0.00098	NA	DEg	down
SDC1	3.04E-12	7.41E-09	0.005618	0.092784	17050.59	723921.6	1	1	0.005618	0.092784	0.060549	-1.21789	0.005618	0.092784	0.060549	18.27404	0.398046	0.776467	0.425795	0.829598	0.000386	0.00102	NA	DEg	down
RBM3	4.44E-10	1.03E-10	0.55618	0.969072	1.42E+09	2.87E+09	1	1	0.542135	0.969072	0.559437	-0.25225	0.55618	0.969072	0.57393	18.23198	0	5.45E-13	0	1.85E-12	0.000394	0.001039	NA	DEg	down
PSMB6	3.17E-11	5.91E-09	0.008427	0.103093	14060.96	781101.1	0.999999	1	0.008427	0.103093	0.081742	-1.08756	0.008427	0.103093	0.081742	18.22408	0.337188	0.384111	0.367766	0.43383	0.000395	0.001041	NA	DEg	down
CDC42SE2	1.24E-08	1.41E-09	0.064607	0.237113	5.51E+08	1.72E+09	1	1	0.064607	0.237113	0.272472	-0.56468	0.064607	0.237113	0.272472	18.20847	0.038633	0.047881	0.04926	0.060176	0.000398	0.001045	NA	DEg	down
NARS	1.24E-08	1.41E-09	0.064607	0.237113	5.51E+08	1.72E+09	1	1	0.064607	0.237113	0.272472	-0.56468	0.064607	0.237113	0.272472	18.20847	0.038633	0.047881	0.04926	0.060176	0.000398	0.001045	NA	DEg	down
PFDN2	8.40E-08	4.80E-09	0.022472	0.14433	129229.8	7298606	1	1	0.022472	0.14433	0.155698	-0.80772	0.022472	0.14433	0.155698	18.16725	0.088091	0.092788	0.105096	0.112816	0.000406	0.001062	NA	DEg	down
RAB5C	8.40E-08	4.80E-09	0.022472	0.14433	129229.8	7298606	1	1	0.022472	0.14433	0.155698	-0.80772	0.022472	0.14433	0.155698	18.16725	0.088091	0.092788	0.105096	0.112816	0.000406	0.001062	NA	DEg	down
BANF1	1.16E-08	2.39E-09	0.070225	0.247423	1.24E+09	9E+08	1	1	0.070225	0.247423	0.283825	-0.54695	0.070225	0.247423	0.283825	18.11614	0.008398	0.005283	0.012172	0.007595	0.000416	0.001085	NA	DEg	down
RPS27L	5.03E-10	0	1.044944	1.597971	6.33E+10	293.856	1	0.994591	0.845506	1.597938	0.529123	-0.27644	1.044944	1.597938	0.653933	18.08391	0	4.66E-06	0	8.54E-06	0.000423	0.0011	NA	DEg	down
CHCHD5	9.35E-09	2.00E-09	0.087079	0.278351	5.56E+09	9.49E+08	1	1	0.087079	0.278351	0.312838	-0.50468	0.087079	0.278351	0.312838	18.03416	0.000924	0.036948	0.001518	0.047382	0.000433	0.001125	NA	DEg	down
TIMM13	7.82E-10	1.46E-10	0.550562	0.958763	7.65E+09	8.12E+09	1	1	0.5	0.958763	0.521505	-0.28274	0.550562	0.958763	0.574242	18.00638	0	0	0	0	0.000439	0.001138	NA	DEg	down
PNRC1	1.59E-09	1.13E-10	0.519663	0.917526	4.9E+10	3.74E+09	1	1	0.438202	0.917526	0.477591	-0.32094	0.519663	0.917526	0.566374	18.00096	8.80E-13	0	3.66E-12	0	0.00044	0.001139	NA	DEg	down
ATP6V0E1	5.85E-09	6.98E-10	0.154494	0.391753	1.93E+12	1.64E+10	1	1	0.151685	0.391753	0.387197	-0.41207	0.154494	0.391753	0.394367	17.87061	1.06E-05	1.48E-06	2.19E-05	2.93E-06	0.000468	0.00121	NA	DEg	down
BTG2	0.660277	1.74E-09	1.083171	0.226804	11763.64	1.21E+09	0.999908	1	0.224719	0.226804	0.990807	-0.00401	0.367978	0.226804	1.622446	17.67	1.87E-07	0.000101	4.56E-07	0.000168	0.000514	0.001328	NA	DEg	up
ADAM9	9.00E-07	3.38E-09	0.019663	0.134021	15371.94	5280704	0.999999	1	0.019663	0.134021	0.146716	-0.83352	0.019663	0.134021	0.146716	17.54629	0.203929	0.387493	0.231501	0.437096	0.000546	0.001404	NA	DEg	down
TYMP	0.748692	4.33E-09	1.296589	0.154639	15268.01	14066667	0.999915	1	0.182584	0.154639	1.180712	0.072144	0.325843	0.154639	2.107116	17.45397	0.00204	0.000104	0.003208	0.000174	0.00057	0.001465	NA	DEg	up
ETFB	6.00E-09	5.22E-10	0.264045	0.556701	6.18E+13	3.16E+10	1	1	0.241573	0.556701	0.433937	-0.36257	0.264045	0.556701	0.474303	17.4135	4.60E-06	1.39E-05	9.92E-06	2.45E-05	0.000581	0.001491	NA	DEg	down
CHMP1B	3.43E-08	4.33E-09	0.02809	0.154639	889536.3	14066667	1	1	0.02809	0.154639	0.181648	-0.74077	0.02809	0.154639	0.181648	17.40408	0.100415	0.253232	0.119477	0.291939	0.000584	0.001495	NA	DEg	down
NFKBIZ	0.787754	2.26E-09	1.045535	0.206186	16788.11	7.19E+08	0.999938	1	0.13764	0.206186	0.667556	-0.17551	0.22191	0.206186	1.076264	17.39185	3.49E-05	0.000397	6.76E-05	0.000624	0.000587	0.001501	NA	DEg	up
QSOX1	1.00E-08	1.41E-09	0.067416	0.237113	9.86E+08	1.72E+09	1	1	0.067416	0.237113	0.284319	-0.54619	0.067416	0.237113	0.284319	17.32517	0.004806	0.002099	0.007238	0.003103	0.000606	0.001545	NA	DEg	down
EFHD2	0.17133	1.17E-10	0.593207	0.845361	10002.07	3.24E+09	0.999941	1	0.370787	0.845361	0.438613	-0.35792	0.491573	0.845361	0.581495	17.24081	3.79E-13	1.88E-06	1.69E-12	3.65E-06	0.000631	0.001605	NA	DEg	down
COX17	6.10E-10	1.03E-10	0.567416	0.969072	1.16E+11	2.87E+09	1	1	0.525281	0.969072	0.542045	-0.26596	0.567416	0.969072	0.585525	17.07813	0	0	0	0	0.000681	0.001731	NA	DEg	down
GRN	4.37E-07	4.46E-09	0.016854	0.123711	12677.25	2419338	0.999999	1	0.016854	0.123711	0.136236	-0.86571	0.016854	0.123711	0.136236	16.96537	0.210168	0.522733	0.237674	0.580091	0.000718	0.00182	NA	DEg	down
RAB7A	4.37E-07	4.46E-09	0.016854	0.123711	12677.25	2419338	0.999999	1	0.016854	0.123711	0.136236	-0.86571	0.016854	0.123711	0.136236	16.96537	0.210168	0.522733	0.237674	0.580091	0.000718	0.00182	NA	DEg	down
LAMTOR4	2.07E-09	1.13E-10	0.530899	0.917526	7.21E+10	3.74E+09	1	1	0.435393	0.917526	0.47453	-0.32374	0.530899	0.917526	0.57862	16.81273	0	3.77E-09	0	9.67E-09	0.000772	0.001944	NA	DEg	down
MAL2	1.43E-09	1.03E-10	0.570225	0.969072	1.13E+10	2.87E+09	1	1	0.477528	0.969072	0.492768	-0.30736	0.570225	0.969072	0.588423	16.79613	0	0	0	0	0.000778	0.001957	NA	DEg	down
LIMA1	0.274334	2.26E-10	0.739341	0.814433	13358.78	2.5E+09	0.999945	1	0.379213	0.814433	0.465617	-0.33197	0.536517	0.814433	0.658761	16.70621	5.60E-14	6.13E-12	2.78E-13	1.94E-11	0.000812	0.002039	NA	DEg	down
SDCBP2	0.299393	2.05E-10	0.890079	0.804124	14074.18	2.81E+09	0.999937	1	0.412921	0.804124	0.513505	-0.28946	0.623596	0.804124	0.775497	16.5646	4.44E-16	6.68E-12	2.78E-15	2.11E-11	0.000868	0.002176	NA	DEg	down
NQO1	6.74E-10	1.13E-10	0.533708	0.917526	4.69E+09	3.74E+09	1	1	0.5	0.917526	0.544944	-0.26365	0.533708	0.917526	0.581682	16.5258	0	5.39E-10	0	1.45E-09	0.000885	0.002213	NA	DEg	down
TAX1BP1	0.27025	2.12E-10	0.619729	0.742268	11464.09	3.92E+09	0.999946	1	0.337079	0.742268	0.45412	-0.34283	0.452247	0.742268	0.609277	16.44876	4.25E-12	1.36E-10	1.58E-11	3.85E-10	0.000917	0.002288	NA	DEg	down
CHMP2A	4.32E-09	1.66E-10	0.488764	0.85567	3.79E+11	1.95E+09	1	1	0.404494	0.85567	0.472722	-0.32539	0.488764	0.85567	0.571206	16.34528	0	3.33E-16	0	1.57E-15	0.000963	0.002399	NA	DEg	down
DNAJB1	3.58E-09	1.91E-10	0.966292	1.463918	1.90E+12	2.42E+09	1	1	0.741573	1.463918	0.506567	-0.29536	0.966292	1.463918	0.660073	16.30246	0	0	0	0	0.000983	0.002445	NA	DEg	down
KLF5	5.54E-09	1.15E-10	0.544944	0.927835	9.05E+12	3.48E+09	1	1	0.429775	0.927835	0.463202	-0.33423	0.544944	0.927835	0.587328	16.17244	0	0	0	0	0.001045	0.002596	NA	DEg	down
ISG15	1.64E-10	2.26E-10	1.294944	0.814433	3.36E+09	2.5E+09	1	1	0.966292	0.814433	1.18646	0.074253	1.294944	0.814433	1.589994	16.14415	0	0	0	0	0.001059	0.002623	NA	DEg	up
NT5C3A	8.40E-08	3.38E-09	0.022472	0.134021	129229.8	5280704	1	1	0.022472	0.134021	0.167675	-0.77553	0.022472	0.134021	0.167675	16.0159	0.052229	0.143706	0.064741	0.169851	0.001126	0.002778	NA	DEg	down
CAPN1	2.64E-09	5.91E-09	0.011236	0.103093	12287.61	781101.1	0.999999	1	0.011236	0.103093	0.108989	-0.96262	0.011236	0.103093	0.108989	15.99976	0.274601	0.644869	0.305494	0.699067	0.001134	0.002791	NA	DEg	down
TUBB2A	2.64E-09	5.91E-09	0.011236	0.103093	12287.61	781101.1	0.999999	1	0.011236	0.103093	0.108989	-0.96262	0.011236	0.103093	0.108989	15.99976	0.274601	0.644869	0.305494	0.699067	0.001134	0.002791	NA	DEg	down
ANXA11	0.221026	2.33E-10	0.667112	0.835052	12399.19	1.74E+09	0.999946	1	0.379213	0.835052	0.45412	-0.34283	0.519663	0.835052	0.622312	15.9636	1.67E-14	4.77E-08	8.67E-14	1.06E-07	0.001154	0.002834	NA	DEg	down
FAM133B	0.13411	7.59E-10	0.236816	0.453608	10128.18	1.8E+09	0.999977	1	0.182584	0.453608	0.402515	-0.39522	0.205056	0.453608	0.452056	15.85902	0.000676	0.000195	0.001115	0.000318	0.001212	0.002973	NA	DEg	down
CALM2	2.63E-10	0	1.235955	1.793854	4.62E+09	275.414	1	0.993529	1.044944	1.793814	0.582526	-0.23468	1.235955	1.793814	0.689009	15.76359	0	0	0	0	0.001268	0.003101	NA	DEg	down
HSPB1	0.011161	2.23E-10	1.295354	1.835052	10002.03	1.38E+09	0.999871	1	0.983146	1.835052	0.535759	-0.27103	1.280899	1.835052	0.698018	15.7425	0	0	0	0	0.00128	0.003127	NA	DEg	down
RPS17	1.31E-10	0	1.747191	2.401934	1.56E+09	284.8135	1	0.991637	1.426966	2.402062	0.594059	-0.22617	1.747191	2.402062	0.727371	15.63237	0	1.42E-07	0	3.09E-07	0.001349	0.003284	NA	DEg	down
CDK2AP2	3.04E-12	1.15E-08	0.005618	0.082474	17050.59	445025.3	1	1	0.005618	0.082474	0.068118	-1.16674	0.005618	0.082474	0.068118	15.61469	0.590892	0.772468	0.615081	0.826318	0.00136	0.003306	NA	DEg	down
DSP	0.163258	2.33E-10	0.597555	0.835052	17402.53	1.74E+09	0.999966	1	0.376404	0.835052	0.450756	-0.34606	0.5	0.835052	0.598765	15.48464	0.0001	5.02E-13	0.000186	1.71E-12	0.001446	0.003505	NA	DEg	down
SERBP1	1.78E-09	1.03E-10	0.58427	0.969072	2.23E+11	2.87E+09	1	1	0.483146	0.969072	0.498566	-0.30228	0.58427	0.969072	0.602917	15.4378	0	1.70E-07	0	3.64E-07	0.001478	0.003578	NA	DEg	down
SNHG8	0.009838	1.13E-10	0.550376	0.917526	10000.09	3.74E+09	0.999945	1	0.418539	0.917526	0.456161	-0.34088	0.544944	0.917526	0.593928	15.41787	3.50E-11	3.33E-16	1.16E-10	1.57E-15	0.001492	0.003606	NA	DEg	down
AP2S1	6.17E-10	1.13E-10	0.544944	0.917526	1.01E+11	3.74E+09	1	1	0.514045	0.917526	0.560251	-0.25162	0.544944	0.917526	0.593928	15.40711	0	8.32E-12	0	2.58E-11	0.0015	0.003614	NA	DEg	down
PDCD5	2.58E-08	3.19E-09	0.042135	0.175258	10877582	49600291	1	1	0.042135	0.175258	0.240416	-0.61904	0.042135	0.175258	0.240416	15.39268	0.085795	0.090348	0.103185	0.110301	0.00151	0.003633	NA	DEg	down
SF3B5	3.47E-09	8.23E-10	0.148876	0.360825	2.49E+12	2.83E+10	1	1	0.148876	0.360825	0.4126	-0.38447	0.148876	0.360825	0.4126	15.11954	0.000113	1.28E-06	0.000208	2.54E-06	0.001717	0.004119	NA	DEg	down
EIF3F	3.87E-09	4.95E-10	0.196629	0.43299	3.91E+13	1.28E+10	1	1	0.19382	0.43299	0.447632	-0.34908	0.196629	0.43299	0.45412	15.04501	0.000526	1.12E-08	0.000886	2.72E-08	0.001779	0.00426	NA	DEg	down
SSBP1	9.35E-09	1.54E-09	0.087079	0.257732	5.56E+09	9.74E+08	1	1	0.087079	0.257732	0.337865	-0.47126	0.087079	0.257732	0.337865	15.00518	0.001489	0.034982	0.002384	0.045122	0.001812	0.004322	NA	DEg	down
ID2	0.749858	2.78E-09	1.021889	0.195876	10848.84	4.98E+08	0.999906	1	0.160112	0.195876	0.817416	-0.08756	0.255618	0.195876	1.304997	14.99327	5.86E-05	0.000318	0.000111	0.000507	0.001822	0.00434	NA	DEg	up
NDUFB11	5.08E-10	1.46E-10	0.581461	0.958763	1.87E+10	8.12E+09	1	1	0.542135	0.958763	0.565452	-0.2476	0.581461	0.958763	0.60647	14.95696	0	3.82E-09	0	9.77E-09	0.001854	0.004406	NA	DEg	down
FAM83H	7.51E-09	2.39E-09	0.081461	0.247423	6.62E+09	9E+08	1	1	0.081461	0.247423	0.329237	-0.48249	0.081461	0.247423	0.329237	14.95459	0.001709	0.032609	0.002706	0.042183	0.001856	0.004406	NA	DEg	down
PKM	8.41E-10	9.87E-11	0.654494	1.051546	5.39E+10	2.73E+09	1	1	0.567416	1.051546	0.539601	-0.26793	0.654494	1.051546	0.622411	14.89982	0	0	0	0	0.001904	0.004515	NA	DEg	down
ERH	4.01E-09	1.64E-09	0.11236	0.298969	9.07E+10	3.02E+09	1	1	0.11236	0.298969	0.375823	-0.42502	0.11236	0.298969	0.375823	14.84995	0.000266	0.000259	0.000466	0.000418	0.001949	0.004615	NA	DEg	down
TMED10	1.38E-09	5.81E-10	0.261236	0.525773	2.98E+11	2.87E+10	1	1	0.261236	0.525773	0.496861	-0.30377	0.261236	0.525773	0.496861	14.82345	0.000551	1.54E-05	0.000922	2.70E-05	0.001974	0.004659	NA	DEg	down
GLUL	3.31E-08	3.38E-09	0.025281	0.134021	378924	5280704	1	1	0.025281	0.134021	0.188634	-0.72438	0.025281	0.134021	0.188634	14.64087	0.459505	0.106772	0.486857	0.128763	0.002151	0.005069	NA	DEg	down
TSPAN1	3.48E-09	1.43E-10	1.115169	1.628866	2.26E+14	1.8E+09	1	1	0.800562	1.628866	0.491484	-0.30849	1.115169	1.628866	0.684629	14.609	0	0	0	0	0.002183	0.005138	NA	DEg	down
LMAN2	3.95E-08	5.91E-09	0.014045	0.103093	16271.31	781101.1	0.999999	1	0.014045	0.103093	0.136236	-0.86571	0.014045	0.103093	0.136236	14.13781	0.366506	0.709528	0.396343	0.763579	0.002723	0.006382	NA	DEg	down
MTPN	3.95E-08	5.91E-09	0.014045	0.103093	16271.31	781101.1	0.999999	1	0.014045	0.103093	0.136236	-0.86571	0.014045	0.103093	0.136236	14.13781	0.366506	0.709528	0.396343	0.763579	0.002723	0.006382	NA	DEg	down
SNRPD1	2.67E-09	7.59E-10	0.219101	0.453608	2.63E+14	1.8E+09	1	1	0.210674	0.453608	0.464441	-0.33307	0.219101	0.453608	0.483018	13.86559	2.47E-06	2.94E-07	5.46E-06	6.13E-07	0.003094	0.00723	NA	DEg	down
TAF1D	0.677597	4.33E-09	1.01067	0.154639	19506.75	14066667	0.999948	1	0.205056	0.154639	1.32603	0.122553	0.325843	0.154639	2.107116	13.85512	2.23E-05	0.0013	4.47E-05	0.001951	0.003109	0.007255	NA	DEg	up
PSMD14	3.06E-08	4.33E-09	0.036517	0.154639	4283965	14066667	1	1	0.036517	0.154639	0.236142	-0.62683	0.036517	0.154639	0.236142	13.82716	0.101531	0.296515	0.120425	0.337898	0.00315	0.007329	NA	DEg	down
SEC11A	3.06E-08	4.33E-09	0.036517	0.154639	4283965	14066667	1	1	0.036517	0.154639	0.236142	-0.62683	0.036517	0.154639	0.236142	13.82716	0.101531	0.296515	0.120425	0.337898	0.00315	0.007329	NA	DEg	down
MRPL41	3.06E-09	2.33E-10	0.382023	0.680412	2.32E+11	7.86E+09	1	1	0.339888	0.680412	0.499532	-0.30144	0.382022	0.680412	0.561457	13.71326	2.99E-14	1.69E-06	1.54E-13	3.29E-06	0.003323	0.007708	NA	DEg	down
MAP2K3	2.64E-09	7.41E-09	0.011236	0.092784	12287.61	723921.6	0.999999	1	0.011236	0.092784	0.121099	-0.91686	0.011236	0.092784	0.121099	13.62066	0.414228	0.800408	0.442042	0.85313	0.00347	0.008027	NA	DEg	down
TBL1XR1	2.64E-09	7.41E-09	0.011236	0.092784	12287.61	723921.6	0.999999	1	0.011236	0.092784	0.121099	-0.91686	0.011236	0.092784	0.121099	13.62066	0.414228	0.800408	0.442042	0.85313	0.00347	0.008027	NA	DEg	down
SMIM22	7.87E-10	2.21E-10	1.134831	1.618557	4.26E+10	1.7E+09	1	1	0.86236	1.618557	0.532795	-0.27344	1.134831	1.618557	0.701138	13.48553	0	0	0	0	0.003696	0.008538	NA	DEg	down
ATP6V1F	0.774976	4.80E-09	0.162279	0.14433	18710.46	7298606	0.999991	1	0.033708	0.14433	0.233547	-0.63163	0.036517	0.14433	0.25301	13.46594	0.267514	0.81992	0.299104	0.872881	0.00373	0.008592	NA	DEg	down
VPS29	3.10E-13	4.34E-07	0.002809	0.061856	15789.74	19084.96	1	0.999997	0.002809	0.061856	0.045412	-1.34283	0.002809	0.061856	0.045412	13.23446	0.611885	0.77214	0.634706	0.826318	0.004156	0.009547	NA	DEg	down
REG4	6.87E-10	0	1.485955	2.041145	2.24E+09	271.732	1	0.992544	1.311798	2.041237	0.642648	-0.19203	1.485955	2.041237	0.727968	13.18808	0	0	0	0	0.004247	0.009742	NA	DEg	down
BRD2	0.677473	2.36E-09	0.897058	0.216495	12927.2	7.89E+08	0.999931	1	0.191011	0.216495	0.88229	-0.05439	0.289326	0.216495	1.33641	13.14334	3.79E-05	0.00119	7.31E-05	0.001808	0.004337	0.009919	NA	DEg	up
ABHD11	2.06E-08	4.80E-09	0.033708	0.14433	228982.1	7298606	1	1	0.033708	0.14433	0.233547	-0.63163	0.033708	0.14433	0.233547	13.04649	0.27847	0.074165	0.309411	0.09155	0.004537	0.010363	NA	DEg	down
ATP5PB	4.07E-09	1.64E-09	0.132022	0.298969	5.51E+11	3.02E+09	1	1	0.132022	0.298969	0.441592	-0.35498	0.132022	0.298969	0.441592	13.04297	0.000235	0.006003	0.000413	0.008534	0.004545	0.010366	NA	DEg	down
ORMDL2	3.04E-12	1.10E-07	0.005618	0.072165	17050.59	18421.9	1	0.999996	0.005618	0.072165	0.077849	-1.10875	0.005618	0.072165	0.077849	13.00565	0.63484	0.918806	0.652434	0.946455	0.004624	0.010518	NA	DEg	down
WASF2	3.04E-12	1.10E-07	0.005618	0.072165	17050.59	18421.9	1	0.999996	0.005618	0.072165	0.077849	-1.10875	0.005618	0.072165	0.077849	13.00565	0.63484	0.918806	0.652434	0.946455	0.004624	0.010518	NA	DEg	down
EPS8L1	0.981455	1.10E-07	0.60586	0.072165	15706.13	18421.9	0.999961	0.999996	0.008427	0.072165	0.116774	-0.93266	0.011236	0.072165	0.155698	12.9478	0.448844	0.392639	0.476128	0.442341	0.004751	0.010776	NA	DEg	down
PPP1R12A	2.23E-08	3.43E-09	0.044944	0.164948	17729617	30780812	1	1	0.044944	0.164948	0.272472	-0.56468	0.044944	0.164948	0.272472	12.66716	0.156335	0.316892	0.179533	0.360196	0.005415	0.012247	NA	DEg	down
PSME2	7.13E-09	1.13E-10	0.575843	0.917526	1.34E+13	3.74E+09	1	1	0.446629	0.917526	0.486776	-0.31267	0.575843	0.917526	0.627604	12.65649	0	1.11E-16	0	5.49E-16	0.005442	0.012291	NA	DEg	down
PIM3	1.18E-08	3.00E-09	0.05618	0.185567	93941155	2.23E+08	1	1	0.05618	0.185567	0.302747	-0.51892	0.05618	0.185567	0.302747	12.54704	0.022235	0.017039	0.029714	0.023259	0.005726	0.012897	NA	DEg	down
SNRPB	6.64E-10	7.94E-10	0.668539	0.371134	7.72E+09	2.47E+10	1	1	0.578652	0.371134	1.559145	0.192886	0.668539	0.371134	1.801342	12.48234	1.11E-16	9.02E-13	7.49E-16	2.96E-12	0.005901	0.013273	NA	DEg	up
EIF6	1.45E-09	2.25E-10	0.401685	0.690722	9.26E+12	6.89E+09	1	1	0.370787	0.690722	0.53681	-0.27018	0.401685	0.690722	0.581545	12.39543	0.001487	1.15E-10	0.002384	3.32E-10	0.006144	0.013801	NA	DEg	down
AKR1B10	9.13E-09	3.00E-09	0.421348	0.185567	2.35E+13	2.23E+08	1	1	0.376404	0.185567	2.028402	0.307154	0.421348	0.185567	2.270599	12.37003	8.91E-07	0.022274	2.07E-06	0.0299	0.006217	0.013946	NA	DEg	up
ANXA1	0.558518	2.26E-09	0.820779	0.206186	17350.04	7.19E+08	0.999953	1	0.247191	0.206186	1.198876	0.078774	0.36236	0.206186	1.757444	12.15578	0.002963	0.001104	0.004594	0.00168	0.006868	0.015342	NA	DEg	up
FUT2	7.51E-09	1.74E-09	0.081461	0.226804	6.62E+09	1.21E+09	1	1	0.081461	0.226804	0.359168	-0.4447	0.081461	0.226804	0.359168	12.05082	0.007654	0.021016	0.011167	0.028383	0.007211	0.016086	NA	DEg	down
ACADVL	0.251646	2.26E-10	0.908361	0.814433	13181.84	2.5E+09	0.999931	1	0.446629	0.814433	0.548393	-0.26091	0.679775	0.814433	0.834661	11.90362	0	1.08E-11	0	3.32E-11	0.007721	0.017144	NA	DEg	down
CPNE3	3.95E-08	7.41E-09	0.014045	0.092784	16271.31	723921.6	0.999999	1	0.014045	0.092784	0.151373	-0.81995	0.014045	0.092784	0.151373	11.90169	0.358958	0.660989	0.389126	0.7148	0.007728	0.017144	NA	DEg	down
SQOR	3.95E-08	7.41E-09	0.014045	0.092784	16271.31	723921.6	0.999999	1	0.014045	0.092784	0.151373	-0.81995	0.014045	0.092784	0.151373	11.90169	0.358958	0.660989	0.389126	0.7148	0.007728	0.017144	NA	DEg	down
EIF3I	2.58E-08	4.33E-09	0.042135	0.154639	10877582	14066667	1	1	0.042135	0.154639	0.272472	-0.56468	0.042135	0.154639	0.272472	11.87545	0.173149	0.061186	0.197315	0.075949	0.007822	0.017331	NA	DEg	down
ESRRA	1.16E-08	2.26E-09	0.070225	0.206186	1.24E+09	7.19E+08	1	1	0.070225	0.206186	0.34059	-0.46777	0.070225	0.206186	0.34059	11.86907	0.013071	0.012216	0.018321	0.016857	0.007845	0.017358	NA	DEg	down
TIMM8B	1.88E-08	3.43E-09	0.047753	0.164948	27413284	30780812	1	1	0.047753	0.164948	0.289501	-0.53835	0.047753	0.164948	0.289501	11.79308	0.268535	0.131606	0.299869	0.156172	0.008127	0.017956	NA	DEg	down
ZFP36	1.88E-09	1.25E-10	0.643258	0.989691	2.96E+12	9.35E+09	1	1	0.497191	0.989691	0.50237	-0.29898	0.643258	0.989691	0.649959	11.78681	0	2.69E-08	0	6.26E-08	0.00815	0.017984	NA	DEg	down
RNF181	1.81E-08	3.00E-09	0.058989	0.185567	92103425	2.23E+08	1	1	0.058989	0.185567	0.317884	-0.49773	0.058989	0.185567	0.317884	11.76849	0.023413	0.037392	0.031101	0.047884	0.00822	0.018088	NA	DEg	down
PSMB3	1.71E-09	1.54E-09	0.505618	0.257732	9.95E+10	9.74E+08	1	1	0.426966	0.257732	1.656629	0.219225	0.505618	0.257732	1.961798	11.77041	4.70E-10	3.65E-07	1.39E-09	7.52E-07	0.008212	0.018088	NA	DEg	up
CLCN3	1.11E-08	3.19E-09	0.053371	0.175258	68654511	49600291	1	1	0.053371	0.175258	0.304527	-0.51637	0.053371	0.175258	0.304527	11.76122	0.039562	0.043533	0.050229	0.055035	0.008248	0.0181	NA	DEg	down
FUOM	1.11E-08	3.19E-09	0.053371	0.175258	68654511	49600291	1	1	0.053371	0.175258	0.304527	-0.51637	0.053371	0.175258	0.304527	11.76122	0.039562	0.043533	0.050229	0.055035	0.008248	0.0181	NA	DEg	down
CLIC1	2.17E-10	0	1.174157	1.639187	4.72E+10	290.9724	1	0.994398	0.985955	1.639175	0.601495	-0.22077	1.174157	1.639175	0.71631	11.69449	0	0	0	0	0.008506	0.018631	NA	DEg	down
PDIA6	7.98E-09	2.39E-09	0.095506	0.247423	5.92E+09	9E+08	1	1	0.095506	0.247423	0.386002	-0.41341	0.095506	0.247423	0.386002	11.68953	0.006413	0.011196	0.009561	0.015473	0.008526	0.018636	NA	DEg	down
MRPL51	4.74E-09	8.93E-10	0.143258	0.319588	1.05E+12	1.35E+10	1	1	0.143258	0.319588	0.44826	-0.34847	0.143258	0.319588	0.44826	11.54772	0.000567	0.002622	0.000948	0.003838	0.009105	0.019847	NA	DEg	down
OST4	2.90E-10	0	1.280899	1.762891	9.61E+08	278.0052	1	0.993699	1.05618	1.762887	0.59912	-0.22249	1.280899	1.762887	0.726592	11.5055	0	0	0	0	0.009284	0.020211	NA	DEg	down
CSTB	2.62E-10	0	1.404494	1.907243	9.35E+08	273.1132	1	0.993065	1.117978	1.907216	0.586183	-0.23197	1.404494	1.907216	0.736411	11.46153	0	6.66E-11	0	1.93E-10	0.009475	0.020599	NA	DEg	down
TMEM59	7.80E-10	1.25E-10	0.581461	0.907216	3.62E+09	3.42E+09	1	1	0.514045	0.907216	0.566618	-0.24671	0.581461	0.907216	0.640928	11.44727	0	0	0	0	0.009538	0.020707	NA	DEg	down
ARL6IP5	2.64E-09	1.15E-08	0.011236	0.082474	12287.61	445025.3	0.999999	1	0.011236	0.082474	0.136236	-0.86571	0.011236	0.082474	0.136236	11.31025	0.505997	0.601838	0.533575	0.656516	0.010161	0.021973	NA	DEg	down
CAPN2	2.64E-09	1.15E-08	0.011236	0.082474	12287.61	445025.3	0.999999	1	0.011236	0.082474	0.136236	-0.86571	0.011236	0.082474	0.136236	11.31025	0.505997	0.601838	0.533575	0.656516	0.010161	0.021973	NA	DEg	down
TPM1	0.009864	1.97E-10	0.964572	1.360825	10000.27	2.15E+09	0.999904	1	0.699438	1.360825	0.513981	-0.28905	0.955056	1.360825	0.701822	11.29973	0	1.23E-14	0	4.73E-14	0.010211	0.022051	NA	DEg	down
ASL	2.06E-08	3.38E-09	0.033708	0.134021	228982.1	5280704	1	1	0.033708	0.134021	0.251513	-0.59944	0.033708	0.134021	0.251513	11.23643	0.599085	0.161199	0.622881	0.189271	0.010514	0.022675	NA	DEg	down
ST13	0.109685	1.39E-10	0.653096	0.886598	12301.36	2.4E+09	0.999947	1	0.426966	0.886598	0.481578	-0.31733	0.581461	0.886598	0.655834	11.21125	0	2.69E-14	0	1.01E-13	0.010637	0.02291	NA	DEg	down
GMDS	9.00E-07	5.91E-09	0.019663	0.103093	15371.94	781101.1	0.999999	1	0.019663	0.103093	0.19073	-0.71958	0.019663	0.103093	0.19073	11.16226	0.297556	0.593251	0.328975	0.650238	0.01088	0.023403	NA	DEg	down
LDHA	2.83E-10	0	1.143258	1.587653	5.05E+09	294.2976	1	0.994634	0.969101	1.587629	0.610408	-0.21438	1.143258	1.587629	0.720104	10.99227	0	0	0	0	0.011768	0.025245	NA	DEg	down
VDAC1	5.38E-10	0	0.660112	1.000015	3.1E+09	419.4838	1	0.997622	0.601124	1	0.601124	-0.22104	0.660112	1	0.660112	10.89952	0	1.98E-05	0	3.45E-05	0.012282	0.02621	NA	DEg	down
TCF25	0.259879	2.12E-10	0.869128	0.742268	14480.17	3.92E+09	0.99994	1	0.429775	0.742268	0.579003	-0.23732	0.643258	0.742268	0.866612	10.83641	3.77E-15	3.17E-09	2.15E-14	8.17E-09	0.012644	0.026948	NA	DEg	down
FLOT1	3.17E-11	1.10E-07	0.008427	0.072165	14060.96	18421.9	0.999999	0.999996	0.008427	0.072165	0.116774	-0.93266	0.008427	0.072165	0.116774	10.80499	0.54957	0.558522	0.57747	0.614443	0.012828	0.027269	NA	DEg	down
RPN2	3.17E-11	1.10E-07	0.008427	0.072165	14060.96	18421.9	0.999999	0.999996	0.008427	0.072165	0.116774	-0.93266	0.008427	0.072165	0.116774	10.80499	0.54957	0.558522	0.57747	0.614443	0.012828	0.027269	NA	DEg	down
UQCRB	1.98E-10	0	1.764045	1.288663	2.47E+09	456.057	1	0.997182	1.466292	1.28866	1.137843	0.056082	1.764045	1.28866	1.368899	10.77924	0	5.87E-09	0	1.47E-08	0.012982	0.027558	NA	DEg	up
PPIG	3.31E-08	5.93E-09	0.025281	0.113402	378924	1203645	1	1	0.025281	0.113402	0.222932	-0.65183	0.025281	0.113402	0.222932	10.71774	0.210499	0.60282	0.237746	0.656516	0.013354	0.028313	NA	DEg	down

COMMD6	2.24E-10	0	1.160112	1.597971	9.64E+10	293.856	1	0.994591	0.977528	1.597938	0.611743	-0.21343	1.160112	1.597938	0.726006	10.53235	0	2.68E-07	0	5.60E-07	0.014543	0.030769	NA	DEg	down
MUCL3	0.761788	4.34E-07	0.919775	0.061856	13910.5	19084.96	0.999934	0.999997	0.143258	0.061856	2.316011	0.364741	0.219101	0.061856	3.542135	10.53118	0.007284	0.049248	0.01075	0.061647	0.014551	0.030769	NA	DEg	up
MRPS33	3.10E-13	1.82E-07	0.002809	0.051546	15789.74	15943.04	1	0.999997	0.002809	0.051546	0.054494	-1.26365	0.002809	0.051546	0.054494	10.48699	0.42959	0.891832	0.456247	0.926734	0.014849	0.031319	NA	DEg	down
YTHDC1	3.10E-13	1.82E-07	0.002809	0.051546	15789.74	15943.04	1	0.999997	0.002809	0.051546	0.054494	-1.26365	0.002809	0.051546	0.054494	10.48699	0.42959	0.891832	0.456247	0.926734	0.014849	0.031319	NA	DEg	down
AUP1	3.04E-12	4.34E-07	0.005618	0.061856	17050.59	19084.96	1	0.999997	0.005618	0.061856	0.090824	-1.0418	0.005618	0.061856	0.090824	10.46065	0.628698	0.887441	0.647617	0.925935	0.01503	0.031577	NA	DEg	down
GTF3C6	3.04E-12	4.34E-07	0.005618	0.061856	17050.59	19084.96	1	0.999997	0.005618	0.061856	0.090824	-1.0418	0.005618	0.061856	0.090824	10.46065	0.628698	0.887441	0.647617	0.925935	0.01503	0.031577	NA	DEg	down
VTI1B	3.04E-12	4.34E-07	0.005618	0.061856	17050.59	19084.96	1	0.999997	0.005618	0.061856	0.090824	-1.0418	0.005618	0.061856	0.090824	10.46065	0.628698	0.887441	0.647617	0.925935	0.01503	0.031577	NA	DEg	down
NDUFB1	4.22E-10	0	0.97191	1.371134	4.57E+09	337.7711	1	0.995957	0.86236	1.371134	0.628939	-0.20139	0.97191	1.371134	0.708837	10.45248	0	1.95E-08	0	4.61E-08	0.015087	0.031621	NA	DEg	down
IDH2	3.41E-08	4.46E-09	0.030899	0.123711	1314645	2419338	1	1	0.030899	0.123711	0.249766	-0.60247	0.030899	0.123711	0.249766	10.44822	0.157735	0.431459	0.180907	0.483625	0.015116	0.031635	NA	DEg	down
MUC3A	3.06E-08	3.38E-09	0.036517	0.134021	4283965	5280704	1	1	0.036517	0.134021	0.272472	-0.56468	0.036517	0.134021	0.272472	10.29205	0.544486	0.074935	0.573483	0.092372	0.01624	0.033812	NA	DEg	down
IL32	0.106659	1.43E-10	1.034495	1.237113	12092.1	3.09E+09	0.999914	1	0.643258	1.237113	0.519967	-0.28402	0.924157	1.237113	0.747027	9.943943	0	0	0	0	0.019049	0.03961	NA	DEg	down
AURKAIP1	1.65E-09	1.03E-10	0.651685	0.969072	4.45E+10	2.87E+09	1	1	0.511236	0.969072	0.527552	-0.27773	0.651685	0.969072	0.672484	9.916378	0	2.92E-08	0	6.78E-08	0.01929	0.040061	NA	DEg	down
CIRBP	1.67E-09	2.12E-10	0.469101	0.742268	2.26E+11	3.92E+09	1	1	0.407303	0.742268	0.548728	-0.26064	0.469101	0.742268	0.631983	9.91171	1.55E-15	6.87E-14	9.41E-15	2.49E-13	0.019332	0.040095	NA	DEg	down
ANKRD36(	0.881582	5.04E-11	1.423262	0.020619	17062.07	13501.04	0.999917	0.999998	0.089888	0.020619	4.359551	0.639442	0.168539	0.020619	8.174157	9.810809	0.155631	0.110768	0.179187	0.133041	0.020245	0.041882	NA	DEg	up
AL121761.	3.95E-08	1.15E-08	0.014045	0.082474	16271.31	445025.3	0.999999	1	0.014045	0.082474	0.170295	-0.7688	0.014045	0.082474	0.170295	9.745267	0.382474	0.603281	0.41161	0.656516	0.02086	0.042991	NA	DEg	down
MAP2K2	3.95E-08	1.15E-08	0.014045	0.082474	16271.31	445025.3	0.999999	1	0.014045	0.082474	0.170295	-0.7688	0.014045	0.082474	0.170295	9.745267	0.382474	0.603281	0.41161	0.656516	0.02086	0.042991	NA	DEg	down
H1F0	0.857442	2.36E-13	0.847276	0.010309	12205.85	10885.52	0.999931	0.999999	0.081461	0.010309	7.901685	0.89772	0.120787	0.010309	11.71629	9.578661	0.149634	0.453113	0.17273	0.506621	0.022509	0.046213	NA	DEg	up
BLOC1S1	5.00E-10	1.46E-10	0.648876	0.958763	2.46E+09	8.12E+09	1	1	0.601124	0.958763	0.626978	-0.20275	0.648876	0.958763	0.676785	9.522409	0	2.51E-10	0	6.97E-10	0.023094	0.047355	NA	DEg	down
RPL22L1	3.72E-10	0	0.955056	1.329898	5.56E+09	342.6162	1	0.996133	0.839888	1.329897	0.631543	-0.1996	0.955056	1.329897	0.718143	9.414271	0	1.05E-08	0	2.54E-08	0.024261	0.049685	NA	DEg	down
