# Supplementary figures and images for: Single Cell Analysis of Gastric Cancer Reveals Non-Defined Telomere Maintenance Mechanism
Source: Cells. 2022 Oct 23;11(21):3342. doi: 10.3390/cells11213342 (PMC9657924; doi:10.3390/cells11213342)

## Slide 1
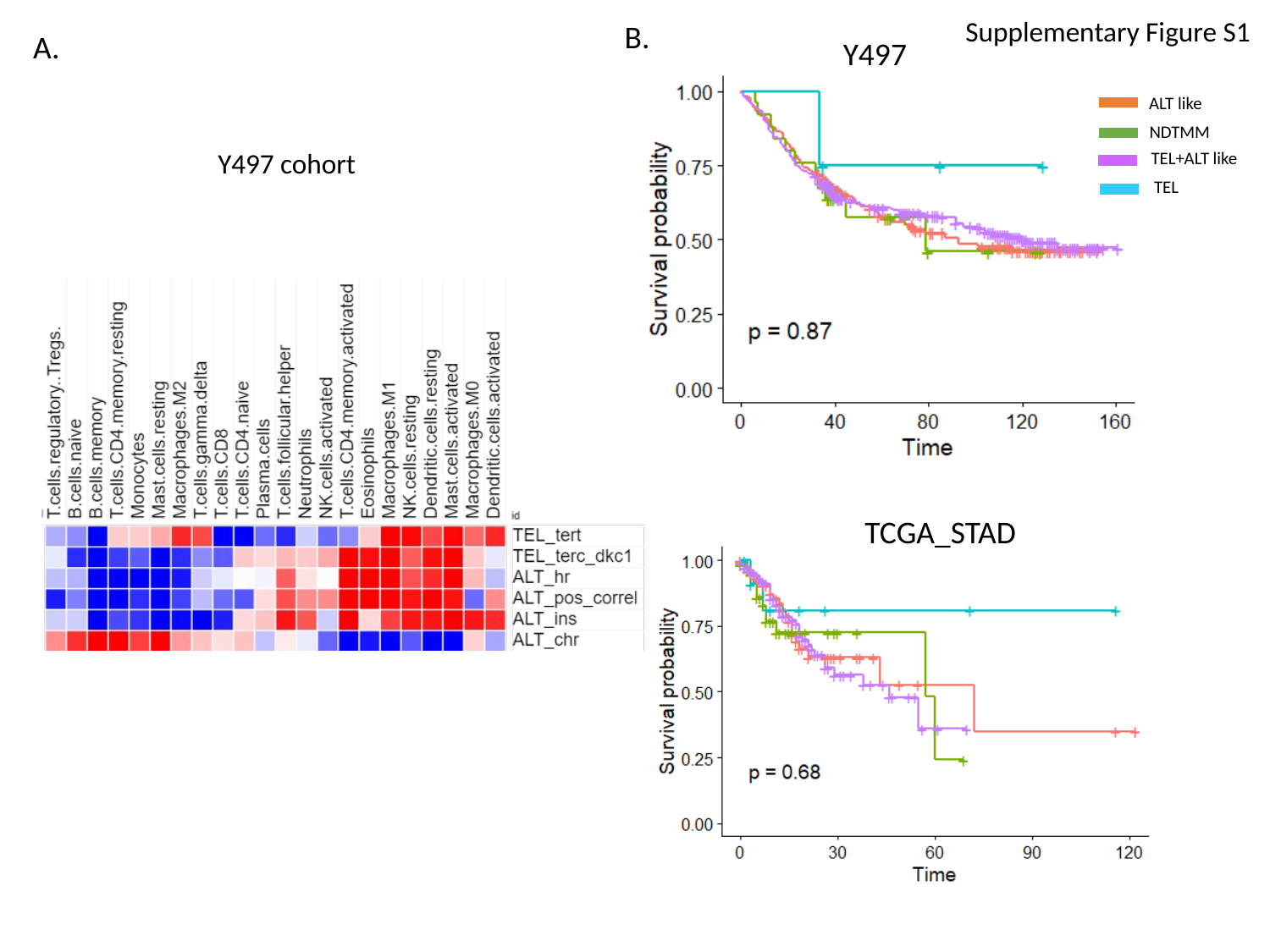

Supplementary Figure S1
B.
A.
Y497
ALT like
NDTMM
Y497 cohort
TEL+ALT like
TEL
TCGA_STAD

Supplement: Supplementary file 1 [file cells-11-03342-s001.zip › supplementary Figure S1.pptx]
